# Site-Selective C6-β-Aminoalkylation of Tetrahydroquinolines with *N*‑Arylsulfonyl Aryl Aziridines in Hexafluoroisopropanol: A Modular Approach to C6-Alkylated Quinolines

**DOI:** 10.1021/acs.joc.5c02643

**Published:** 2026-01-09

**Authors:** Yunus Taskesenligil, Murat Aslan, Nurullah Saracoglu

**Affiliations:** † Department of Chemistry, Faculty of Sciences, 37504 Atatürk University, Erzurum 25240, Türkiye; ‡ Biotechnology Institute, Ankara University, Ankara 06135, Türkiye

## Abstract

We report a metal-free method for the site-selective C6-β-aminoalkylation of tetrahydroquinolines using *N*-arylsulfonyl aziridines in HFIP at room temperature. HFIP activates aziridines through hydrogen bonding, promoting a regioselective S_N_2-type ring opening. The method features broad functional group tolerance, high yields (up to 89%), and full stereochemical fidelity with chiral aziridines. Gram-scale synthesis with solvent recovery highlights its practicality, while postsynthetic oxidation provides direct access to C6-alkylated quinolines.

## Graphical abstract

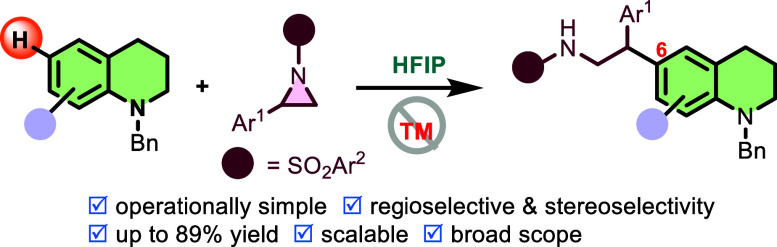

## Introduction

Tetrahydroquinolines (THQs) are privileged scaffolds with diverse biological activities and a wide occurrence in natural products and pharmaceuticals.^1^ Their derivatives display antiviral,^2^ antibacterial,^3^ antimalarial,^4^ anticancer,^5^ and neuroprotective properties,^6^ while also finding use in catalysis,^7^ materials science,^8^ and energy technologies.^9^

Aziridines represent powerful synthetic intermediates whose ring strain enables diverse reactivity patterns, including cycloadditions,^10^ ring enlargements,^11^ and regioselective ring-opening reactions,^12^ serving as versatile building blocks for complex nitrogen-containing compounds (Scheme 1a).^13^ These heterocycles provide efficient access to β-phenethylamine derivatives found in numerous pharmaceutically active compounds.^14^ Unlike conventional transition metal-catalyzed approaches, aziridine-based strategies offer direct ring-opening pathways under milder conditions.^13^

**Scheme 1:**
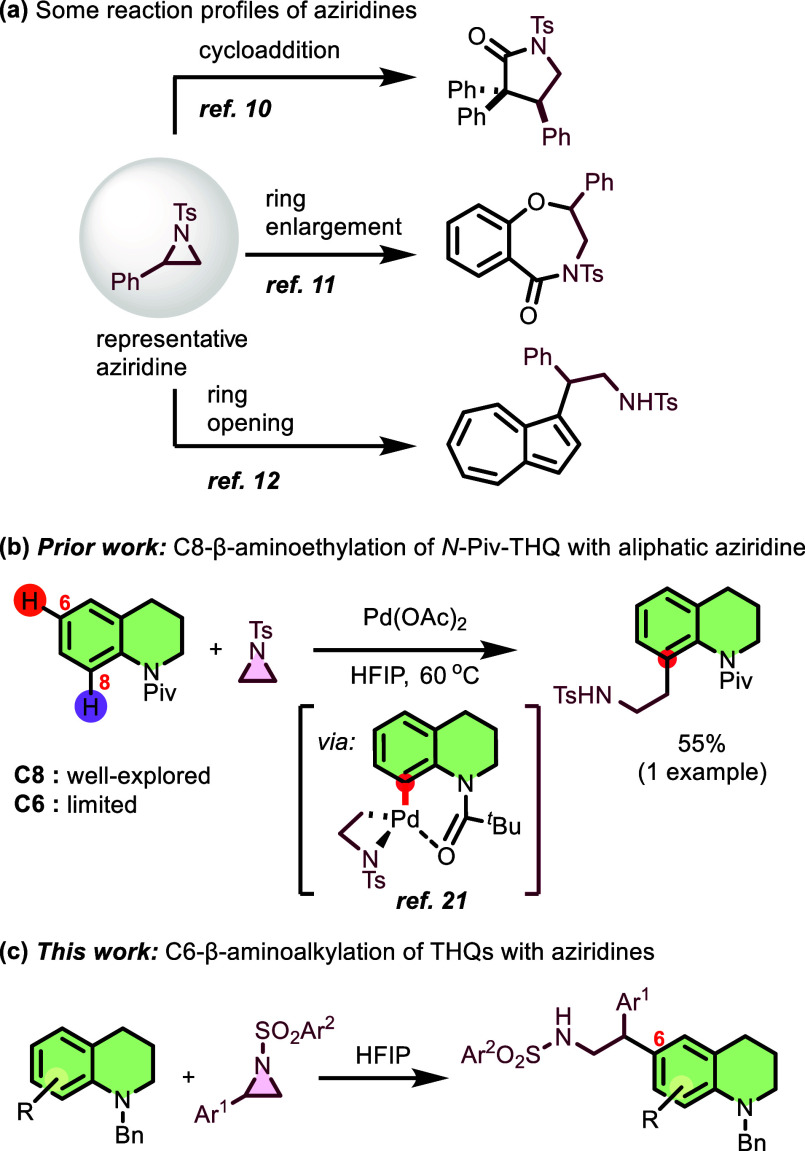
Applications Involving Aziridines

Over the past decade, significant progress has been made in transition metal-catalyzed C–H functionalization of THQs, particularly at the C8 position, including chlorination,^15^ acylation,^16^ amination,^17^ hydroxylation,^18^ arylation,^19^ and alkylation reactions.^20^ These palladium-, rhodium-, or ruthenium-catalyzed transformations have expanded the synthetic utility of THQs through nitrogen-based directing groups. In 2022, Ahmad et al. reported a palladium-catalyzed C–H activation strategy for β-arylethylamine synthesis, establishing cross-coupling between aliphatic aziridines and nitrogen-containing heterocycles, including THQs (Scheme 1b).^21^ However, the substrate scope for THQs remained limited, with primarily focusing on C8-modifications. Despite recent advancements, regioselective functionalization at the C6-position remains largely unexplored, with only sporadic examples such as the β-diketiminate-supported aluminum complex-catalyzed Michael addition by Liu and Vidović^22^ and Ru­(II)-catalyzed *para*-selective C–H difluoromethylation reported by Yuan et al.^23^ Additionally, in 2021, Prusty et al. introduced Bi­(III)-catalyzed regioselective C6 alkylation of THQs toward bioactive core-biaryl oxindoles,^24^ while Zong et al. recently explored tunable electrochemical selenization, achieving selective C3 and C6 substitution.^25^ Despite these advancements, existing C6-functionalization methods remain limited in both substrate scope and reaction efficiency, often requiring harsh reaction conditions or expensive catalysts. Unlike these methods that show limited substrate scope, our protocol offers a metal-free approach under mild conditions, making it highly desirable for the development of new, broadly applicable methodologies. Considering the importance of β-phenethylamine and THQ derivatives in medicinal chemistry, we focused on selective β-aminoalkylation at the C6 position of the THQs. Building upon our recent studies on solvent-controlled, regioselective C6-alkylation via *para*-quinone methides under metal-free conditions,^26^ we report regioselective β-aminoalkylation of THQs at the C6-position using *N*-arylsulfonyl aryl aziridines (Scheme 1c). This metal-free protocol offers significant advantages: (1) catalyst-free, site-selective C6-functionalization with stereochemical fidelity; (2) broad substrate scope and functional group tolerance; (3) gram-scale synthesis with recoverable solvent; and (4) access to diverse polycyclic scaffolds via postsynthetic elaboration, with hexafluoroisopropanol (HFIP) serving dual roles as solvent and activator through its exceptional hydrogen-bond-donating capability and high ionizing power.^27^

## Results and Discussion

As part of our continuous efforts on the metal-free alkylation of tetrahydroquinolines,^26^ initially, we selected *N*-benzyltetrahydroquinoline (**1a**) and *N*-tosylphenylaziridine (**2a**) as the model substrates to optimize the C6-β-aminoalkylation conditions (Table 1). Gratifyingly, the reaction proceeding in HFIP at room temperature for 16 h gave the C6-β-aminoalkylated product **3aa** in 80% yield (entry 1). Subsequently, other solvents were explored. However, the replacement of HFIP with other solvents, such as trifluoroethanol (TFE) and DCE, resulted in a significant loss of reactivity, giving only 65% and no detectable yields, respectively (entries 2 and 3). Protic solvents such as MeOH (entry 4) and *i*PrOH (entry 5) were also tested to assess the effect of different hydrogen-bond donors but showed no reactivity, proving inferior to HFIP due to their weaker hydrogen-bonding ability and lower capacity to activate the aziridine substrate. Using an HFIP-containing mixed solvent (HFIP/DCE = 1:1) provided a lower yield of 68% (entry 6). The reaction using TfOH (5 and 10 mol %) as a catalyst in HFIP furnished the desired product **3aa** in 76% and 72% yields, respectively (entries 7 and 8). Other Lewis acid catalysts, such as B­(C_6_F_5_)_3_, Zn­(OTf)_2_, and Ca­(NTf_2_)_2_ (10 mol % each), gave the product in 73%, 71%, and 75% yields, respectively (entries 9–11). Increasing the temperature to 80 °C, with TfOH as a catalyst, decreased the yield to 63% (entry 12), while running the reaction at 80 °C without a catalyst resulted in a 78% yield (entry 13). Additional optimization studies examining substrate stoichiometry (entry 14, 67% yield), reaction time (entries 15–16, 66–75% yield), and HFIP volume (entries 17–18, 78–76% yield) confirmed the optimality of the initial conditions. When equimolar amounts of substrates were used (entry 14), unreacted aziridine remained and was difficult to separate from the product, whereas the 2:1 ratio (THQ:aziridine) facilitated easier chromatographic separation due to the lower polarity of excess THQ (entries 1 and 14). It should be noted that yields are reported based on the limiting reagent (aziridine). Under the optimized 2:1 stoichiometry (entry 1), approximately 40% of the initial THQ is converted to the desired product, while unreacted THQ is easily recovered. When equimolar substrates were used (entry 14), approximately 67% of THQ was converted; however, the 2:1 ratio was preferred due to easier purification. Finally, the optimal C6-β-aminoalkylation conditions were established using HFIP (2.0 mL) as the solvent at room temperature for 16 h, employing *N*-benzyltetrahydroquinoline (1 mmol, 2.0 equiv) and *N*-tosylphenylaziridine (0.5 mmol, 1.0 equiv) (entry 1).

**Table 1:**
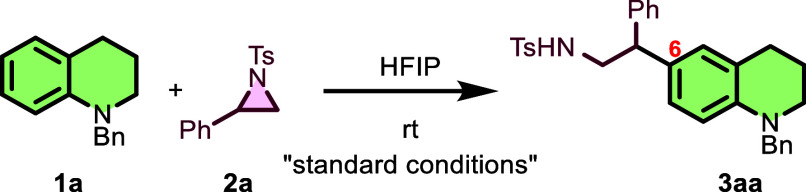
Optimization of the C6-β-Aminoalkylation Conditions^a^

Entry	Variation from the standard conditions	Yield (%)^b^
1	none	80
2	TFE as a solvent	65
3	DCE as a solvent	-
4	MeOH as a solvent	-
5	*i*PrOH as a solvent	-
6	HFIP/DCE (1:1) as a solvent	68
7	TfOH (5 mol %) in HFIP	76
8	TfOH (10 mol %) in HFIP	72
9	B(C_6_F_5_)_3_ (10 mol %) in HFIP	73
10	Zn(OTf_2_)_2_ (10 mol %) in HFIP	71
11	Ca(NTf_2_)_2_ (10 mol %) in HFIP	75
12	TfOH (10 mol %) in HFIP at 80 °C	63
13^c^	at 80 °C	78
14	**1a** (0.5 mmol, 1.0 equiv), **2a** (0.5 mmol, 1.0 equiv)	67
15	6 h reaction time	66
16	12 h reaction time	75
17	HFIP (1 mL)	78
18	HFIP (3 mL)	76

a Reaction conditions: **1a** (1 mmol, 2.0 equiv), **2a** (0.5 mmol, 1.0 equiv) in solvent (2.0 mL) at room temperature for 16 h.

b Isolated yields. Unless otherwise stated, all yields reported in this study were calculated based on aziridine as the limiting reagent.

c In a sealed tube.

As presented in Scheme 2, with the optimal reaction conditions established, the substrate scope of the C6-β-aminoalkylation of *N*-benzyltetrahydroquinoline (**1a**) with various *N*-protected aryl aziridines (**2a–2q**) was explored. Initially, the effect of substituents on the phenyl ring in *N*-tosyl aziridines (**2b–2m**) was investigated (Scheme 2a). Tetrahydroquinolines (**1a–1e**) afforded the desired C6-β-aminoalkylated products (**3ab–3ae**) in excellent yields (89%, 81%, 78%, and 75%, respectively). Notably, both electron-donating (Me) and electron-withdrawing (F, Cl, and Br) groups at the *ortho* position on the phenyl ring were well tolerated. The reaction of *meta*-substituted phenylaziridines bearing electron-withdrawing and electron-donating groups, such as Br and Me, smoothly furnished products **3af** and **3ag** in 76% and 80% yields, respectively.

**Scheme 2:**
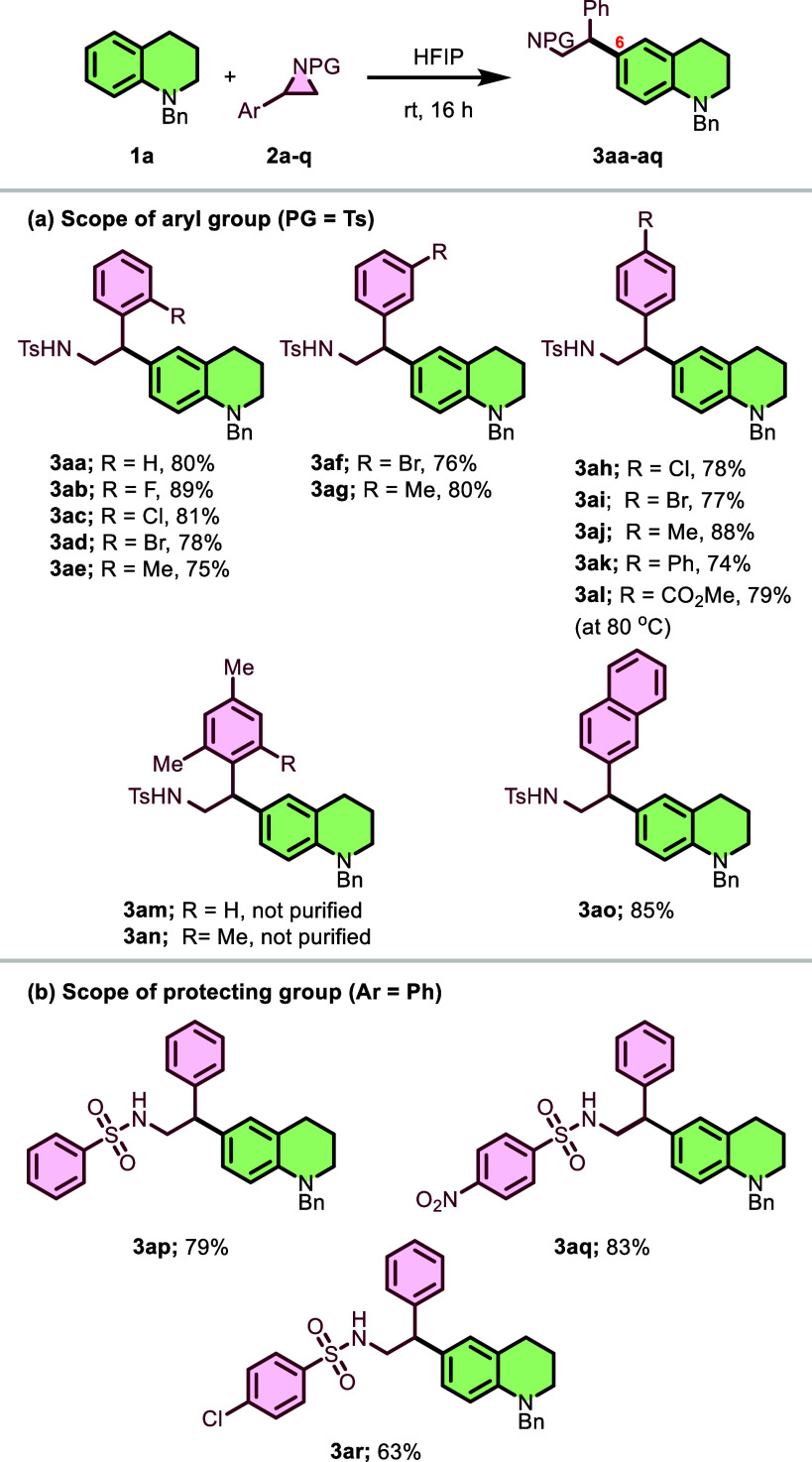
Substrate Scope of *N*-Protected Aziridines

The *para*-substituted phenylaziridines (Cl, Br, Me, and Ph) were also compatible with the reaction, affording products **3ah–3al** in 78–88% yields. For the 2,4-dimethyl- and 2,4,6-trimethyl-substituted phenylaziridines (**2m** and **2n**), the corresponding products, **3am** and **3an**, were formed, but they could not be purified (Scheme 2a). Notably, the reaction with naphthyl aziridine proceeded smoothly to give **3ao** in 85% yield. Furthermore, variations (**2p–2r**) of the protecting group on the aziridine nitrogen while keeping the phenyl group constant were examined (Scheme 2b). *N*-Benzenesulfonyl-, *N*-*para*-nitrobenzenesulfonyl-, and *N*-*para*-chlorobenzenesulfonyl-protected phenylaziridines were compatible with the optimal reaction conditions, furnishing the corresponding products **3ap**, **3aq**, and **3ar** in favorable yields of 79%, 83%, and 63%, respectively.

Furthermore, we explored the substrate scope of the reaction between various *N*-protected tetrahydroquinones **1b**–**1p** and *N*-tosylphenylaziridine **2a**, as illustrated in Scheme 3. The variation (**1b–1n**) of substituents on the *N*-benzyltetrahydroquinone backbone was examined (Scheme 3a). First, tetrahydroquinolines (**1b** and **1c**) bearing electron-withdrawing groups such as Cl and Br at the 5-position reacted efficiently to provide **3ba** and **3ca** in favorable yields (78% and 71%). The 5-methyl- and 5-methoxy-substituted derivatives **1d** and **1e** also performed well, providing products **3da** and **3ea** in 74% and 63% yields, respectively. Tetrahydroquinolines (**1f–1i**) with substituents at the 7-position were also investigated. The reaction with 7-Cl-, 7-Br-, 7-Me-, and 7-OMe-substituted tetrahydroquinolines (**2f–2i**) proceeded smoothly to produce the corresponding alkylation products **3fa**, **3ga**, **3ha**, and **3ia** in yields ranging from 68% to 82%.

**Scheme 3:**
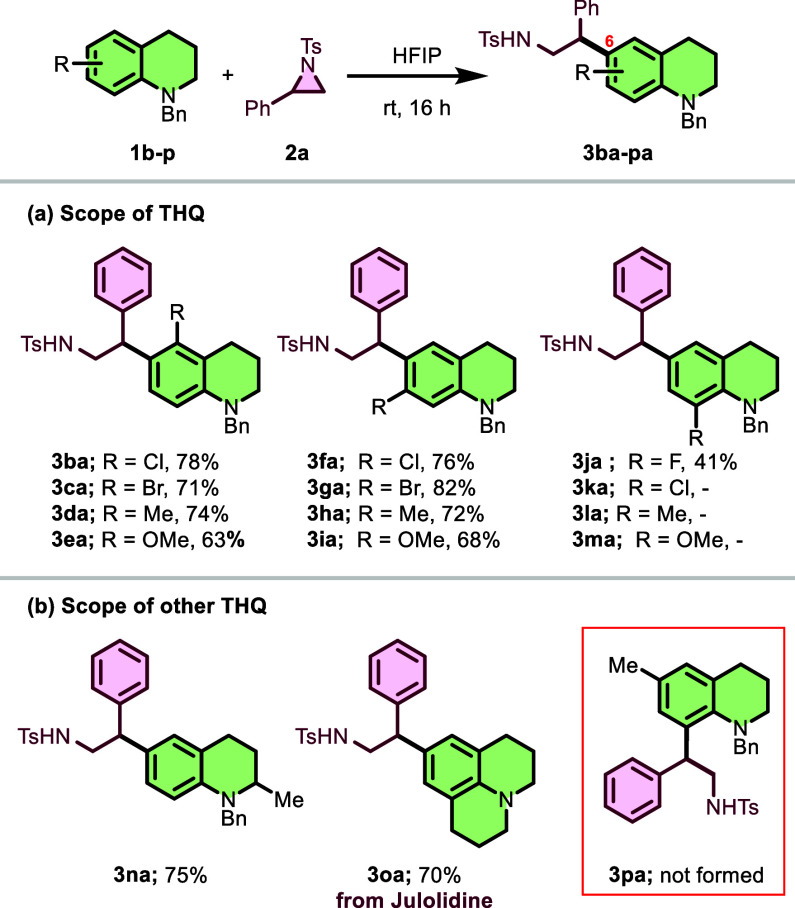
Substrate Scope of *N*-Protected Tetrahydroquinones

When tetrahydroquinolines with eight-position substituents were tested, 8-F-substituted tetrahydroquinoline **1j** gave product **3ja** in 41% yield (Scheme 3a). However, reaction products from 8-Cl, 8-Me, and 8-OMe derivatives (**1k–1m**) could not be purified, despite their successful formation. The reduced efficiency with 8-position substituents may be attributed to competing directing effects as both the amine group and 8-position substituents are *ortho*–*para* directors. Additionally, other structural variations of the tetrahydroquinoline core were explored (Scheme 3b). The reaction with *N*-benzyl-2-methyltetrahydroquinoline (**1n**) proceeded well to give **3na** in 75% yield. Notably, functionalization was also successful on tricyclic analogs such as julolidine (**1o**), furnishing product **3oa** with 70% yield. These results demonstrate the versatility of this methodology and its tolerance toward various functional groups, offering a valuable approach for accessing structurally diverse *N*-protected tetrahydroquinone derivatives. When the 6-position of the tetrahydroquinoline was presubstituted (blocked), as in substrate **1p**, no product **3pa** formation was observed, confirming that the C6-position is essential for this functionalization reaction (Scheme 3b).

To demonstrate the practical utility of this protocol, a gram-scale reaction was successfully performed by subjecting substrate **2a** (3.7 mmol, 1.0 g) to the optimized conditions with **1a**, delivering product **3aa** in 78% yield (Scheme 4a). Notably, 83% of HFIP was successfully recovered from the crude mixture via simple distillation. The synthetic utility of the obtained product was further showcased through functional group manipulations. Subsequent debenzylation of **3aa** under standard hydrogenolysis conditions delivered NH-free tetrahydroquinoline **4** in 95% yield, which could be oxidized to the corresponding quinoline **5** in 85% yield (Scheme 4a). This two-step postsynthetic modification highlights both the robustness and the scalability of the method while enabling access to structurally diverse heterocyclic scaffolds. Additionally, the potential for further molecular elaboration was explored through C–Br to N–C bond conversion reactions using brominated intermediates **3ad** and **3ga** (Scheme 4b). Copper-catalyzed intramolecular C–N coupling provided indoline-substituted **6** and indoline-fused **7** in 82% and 86% yields, respectively, both representing polycyclic motifs typical of bioactive alkaloids and natural products. These transformations underscore the synthetic flexibility of the products and the broader applicability of the method for accessing complex nitrogen-containing heterocycles. The synthetic utility was further exemplified through late-stage functionalization, where borneol and *L*-menthol derivatives **3as** and **3at** were successfully obtained in 83% and 87% yields, respectively, demonstrating the method’s tolerance toward complex natural product scaffolds and its potential for molecular diversification (Scheme 4c).

**Scheme 4:**
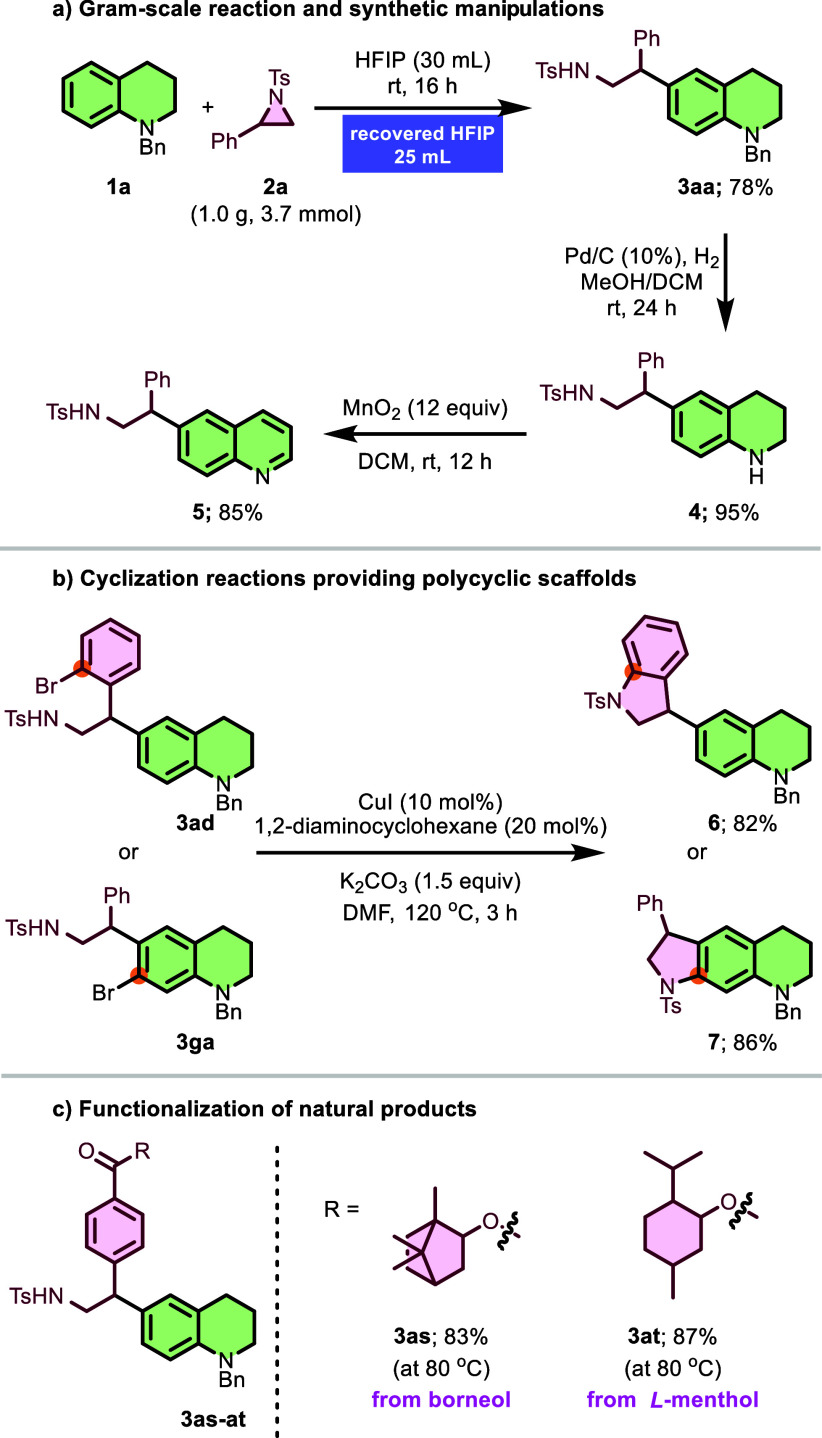
Scaling-up and Derivatizations

To gain deeper insights into the reaction pathway of C6-β-aminoalkylation, a series of control experiments was conducted. When the reaction was conducted in the presence of radical scavengers TEMPO or BHT, the desired product was obtained in 55% and 80% yield, respectively (Scheme 5a). This suggests that the transformation does not proceed through a radical pathway. To explore the stereospecific nature of this transformation, we investigated the reaction between tetrahydroquinoline **1a** and enantiomerically enriched aziridine **(** *R* **)-2a** (98:2 er) (Scheme 5b). Under standard conditions, the reaction proceeded with complete stereochemical fidelity, delivering **(** *R* **)-3aa** in 80% yield, while maintaining the original enantiomeric ratio (98:2 er). This complete retention of stereochemistry strongly supports an S_N_2-type ring-opening mechanism, as any competing S_N_1 pathway involving carbocation intermediates would result in significant racemization. The stereospecific nature confirms that nucleophilic attack occurs with configuration inversion at the benzylic carbon, consistent with the proposed concerted ring-opening process.

**Scheme 5:**
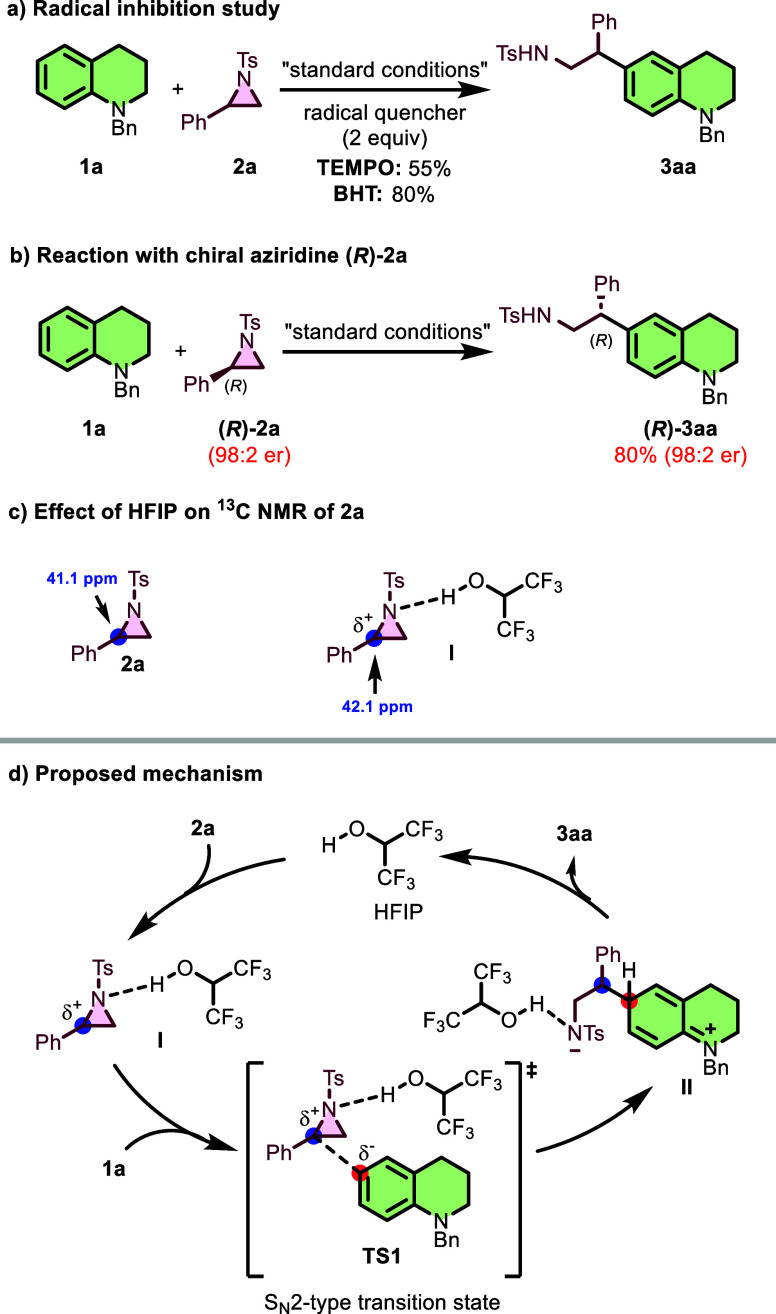
Mechanistic Experiments and Proposed Mechanism

To further probe the mechanism, aziridine **2a** was analyzed by ^13^C and ^1^H NMR spectroscopy in CDCl_3_, both in the absence of and in the presence of 10 equiv of HFIP (Scheme 5c). In the ^13^C NMR spectrum, the benzylic carbon bearing the phenyl group resonated at δ 41.1 and shifted downfield to δ 42.1 ppm upon HFIP addition. This 1 ppm downfield shift indicates hydrogen bond formation between HFIP and the tosyl-protected nitrogen, reducing the electron density on nitrogen and decreasing the electron density of the benzylic carbon through C–N bond polarization. In the ^1^H NMR spectrum, the benzylic proton signal exhibited a slight shift from δ 3.77 to δ 3.76 ppm. More notably, one diastereotopic proton of the CH_2_ group shifted from δ 2.39 to δ 2.52 ppm, while the other remained nearly unchanged. This selective downfield shift results from the asymmetric magnetic environment created by the specific orientation of HFIP upon hydrogen bonding. Based on control experiments and literature precedent,^26^ a plausible mechanism was proposed (Scheme 5d). Initially, HFIP activates the aziridine through hydrogen bonding (intermediate **I**), facilitating ring-opening via an S_N_2-type transition state (**TS1**) upon nucleophilic attack by **1a**. The resulting intermediate **II** undergoes rearomatization to afford the desired product **3aa**.

## Conclusion

In summary, we have developed a metal-free, catalyst-free protocol for the regioselective C6-β-aminoalkylation of tetrahydroquinolines using *N*-arylsulfonyl aziridines. The reaction proceeds under mild conditions at room temperature, with HFIP serving as both solvent and activator, providing products in good to excellent yields. The method exhibits a broad substrate scope and maintains stereochemical integrity when using chiral aziridines. Control experiments support a mechanism involving the HFIP-mediated activation of aziridines through hydrogen bonding. The practical utility was demonstrated through gram-scale synthesis and successful derivatization of products, enabling access to diverse heterocyclic scaffolds and quinoline derivatives. This approach offers significant advantages, including operational simplicity, environmental friendliness through solvent recyclability, and the absence of expensive metal catalysts, providing a practical alternative to the existing harsh C6-functionalization methods.

## Supplementary Material



## Data Availability

The data underlying this study are available in the published article and its online Supporting Information.

## Notes

Experimental procedures, characterization data (^1^H, ^13^C­{^1^H} NMR, HRMS), and NMR and HRMS spectra for compounds (PDF)
