# Supplementary material for: Site-Selective C6-β-Aminoalkylation of Tetrahydroquinolines with N‑Arylsulfonyl Aryl Aziridines in Hexafluoroisopropanol: A Modular Approach to C6-Alkylated Quinolines
Source: J Org Chem. 2026 Jan 9;91(3):1405–11. doi: 10.1021/acs.joc.5c02643 (PMC12836323; doi:10.1021/acs.joc.5c02643)
Supplement: Supplementary file 1 [file jo5c02643_si_001.pdf]

Supporting Information

Site-Selective C6-β-Aminoalkylation of Tetrahydroquinolines with *N*-Arylsulfonyl Aryl Aziridines in Hexafluoroisopropanol: A Modular Approach to C6-Alkylated Quinolines

Yunus Taskesenligil,[†] Murat Aslan,[†] Nurullah Saracoglu*[†,‡]

[†]Department of Chemistry, Faculty of Sciences, Atatürk University, Erzurum, 25240, Türkiye; [‡]Biotechnology Institute, Ankara University, Ankara, 06135, Türkiye

E-mail: saracoglu@ankara.edu.tr

Table of Contents

1. General Information **S3**

2. General Procedures and Synthesis **S3-S6**

3. Characterization Data for Compounds **S6-S18**

4. Gram-scale synthesis of 3aa and recovery of HFIP **S19**

5. Mechanistic experiments **S19-S20**

6. References **S21**

7. NMR Spectra of Compounds **S22-S56**

EXPERIMENTAL SECTION

1. General Information

Proton NMR (^{1}H NMR) measurements were conducted using 400 MHz Varian and Bruker spectrometers. Chemical shifts are expressed in parts per million (δ) downfield from tetramethylsilane, using the residual solvent peak as internal reference (δ 7.26 ppm for $CHCl_3$ in $CDCl_3$). Multiplicities are designated as: s (singlet), d (doublet), dd (doublet of doublets), t (triplet), q (quartet), and m (multiplet). Coupling constants (*J* values) are reported in Hz. Carbon NMR ($^{13}C\{^1H\}$ NMR) spectra were acquired at 101 MHz on the same instruments, with chemical shifts referenced to the solvent carbon signal (δ 77.16 ppm for the central line of $CDCl_3$). High-resolution mass spectrometry (HRMS) and atmospheric pressure chemical ionization mass spectrometry (APCI-MS) were carried out on an Agilent 6530 Accurate-Mass Q-TOF LC/MS system.

Commercial reagents were used as received unless specified otherwise. Analytical thin-layer chromatography was performed on silica gel 60 F254 pre-coated aluminum plates. Flash column chromatography used silica gel (60 Å pore size, 70-230 mesh). Melting point determinations were made using a Büchi 539 melting point apparatus and are reported uncorrected.

2. General Procedures and Synthesis

General Procedure A: Synthesis of Tetrahydroquinolines (Sa–m)[1,2]

According to literature-known procedures,[1] which were also employed in our recent study,[2] tetrahydroquinolines (**2Sa–n**) were synthesized and the structures of the obtained compounds were confirmed by spectroscopic analyses (Scheme S1). The spectroscopic data for tetrahydroquinoline **2Sp** were in agreement with previously reported literature.[3] The tetrahydroquinolines **1n** and **1o** were commercially obtained and used in the subsequent step.

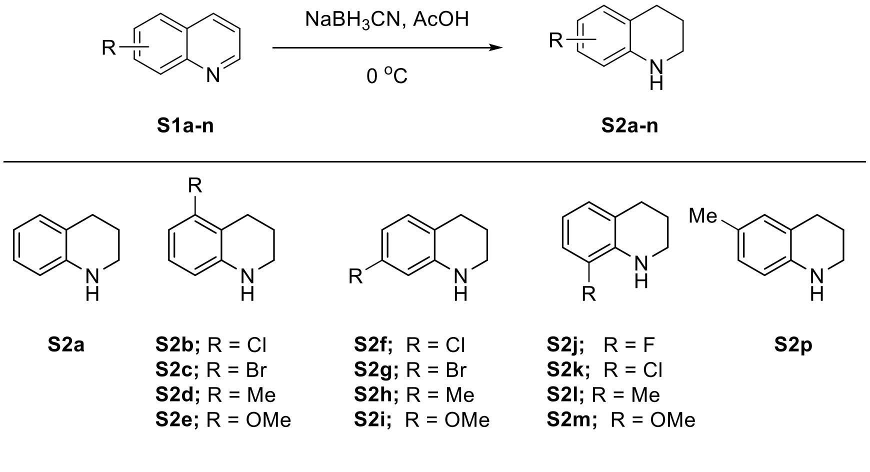

Scheme S1

General Procedure B: Synthesis of *N*-Benzyltetrahydroquinolines (1a–n)[1,2]

N-Benzyltetrahydroquinolines (**1a–p**) were prepared from tetrahydroquinolines (**2Sa–n**) following literature procedures[1] and the methodology reported in our recent study,[2] with their structures confirmed by spectroscopic analysis (Scheme S2). All spectroscopic data for tetrahydroquinoline **1p** were in agreement with previously reported literature.[4]

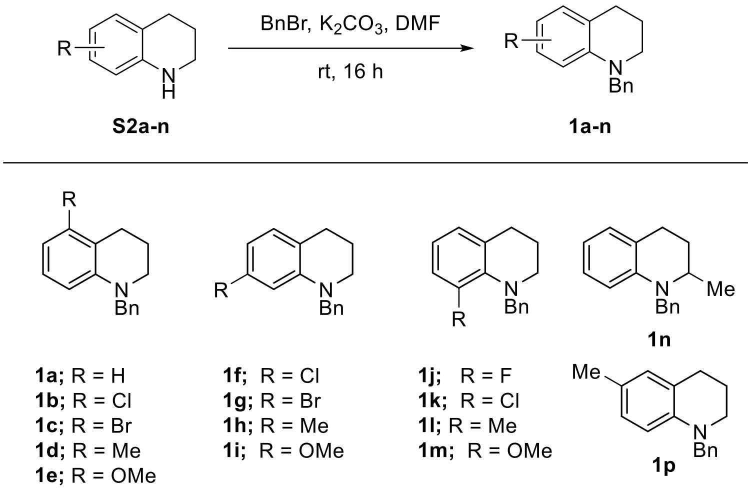

Scheme S2

General procedure C: Synthesis of Aziridines (2a–t)[5]

To a stirred solution of alkene (**S3a–t**; 2.0 mmol, 1.0 equiv) and benzyltriethylammonium chloride (23 mg, 0.1 mmol, 0.05 equiv) in CH_2Cl_2/H_2O (2:1, 15 mL), chloramine-T (**S4**; 501 mg, 2.2 mmol, 1.1 equiv) and iodine (51 mg, 0.2 mmol, 0.1 equiv) were added at room temperature (Scheme S3). The reaction mixture was stirred at room temperature for 48 h. Upon completion, the reaction was quenched with saturated aqueous $Na_2S_2O_3$ (5 mL). The mixture was then extracted with CH_2Cl_2 (3 × 10 mL). The combined organic layers were dried over Na_2SO_4, filtered, and concentrated under reduced pressure. The crude product was purified by silica gel column chromatography using EtOAc/petroleum ether (5:95) as eluent to afford the corresponding aziridines **2a–t**. Compounds **2a**,[5] **2b**,[5] **2c**,[5] **2d**,[5] **2e**,[5] **2f**,[5] **2g**,[5] **2h**,[5] **2i**,[5] **2k**,[5] **2l**,[6] **2m**,[5] **2n**,[5] **2o**,[5] **2p**,[5] **2q**,[5] **2r**[5] and **2t**[6] were prepared according to the above procedure, and all spectroscopic data were in agreement with previously reported literature.

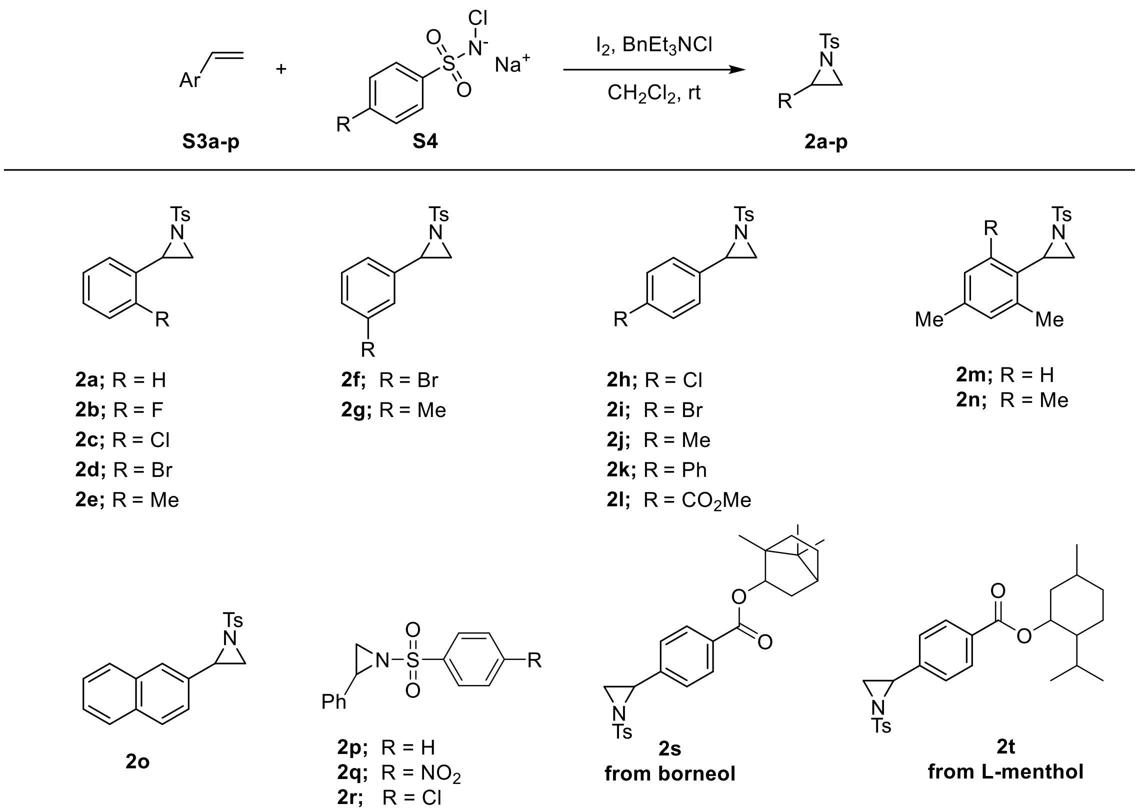

Scheme S3

1,7,7-Trimethylbicyclo[2.2.1]heptan-2-yl 4-(1-tosylaziridin-2-yl)benzoate (2s). Following the general procedure C, (1,7,7-trimethylbicyclo[2.2.1]heptan-2-yl 4-vinylbenzoate (570 mg, 2.0 mmol), benzyltriethylammonium chloride (23 mg, 0.1 mmol, 0.05 equiv), chloramine-T (**S4**; 501 mg, 2.2 mmol, 1.1 equiv), and iodine (51 mg, 0.2 mmol, 0.1 equiv) were used in CH_2Cl_2/H_2O (2:1, 15 mL), affording the title compound as a colorless oil (336 mg, 37%) by using hexane/EtOAc (9:1) as eluent. **^{1}H NMR (400 MHz, CDCl$_3$) δ** 7.99 – 7.94 (m, AA'BB' system, AA' part, 2H), 7.89 – 7.82 (m, AA'BB' system, AA' part, 2H), 7.36 – 7.31 (m, AA'BB' system, BB' part, 2H), 7.31 – 7.26 (m, AA'BB' system, BB' part, 2H), 5.12 – 5.05 (m, 1H), 3.79 (dd, *J* = 7.2, 4.3 Hz, 1H), 3.02 (d, *J* = 7.2 Hz, 1H), 2.51 – 2.43 (m, 1H), 2.42 (s, 3H), 2.38 (d, *J* = 4.3 Hz, 1H), 2.13 – 2.02 (m, 1H), 1.85 – 1.74 (m, 1H), 1.72 (t, *J* = 4.5 Hz, 1H), 1.44 – 1.34 (m, 1H), 1.33 – 1.21 (m, 2H), 1.08 (dd, *J* = 13.8, 3.4 Hz, 1H), 0.95 (s, 3H), 0.90 (s, 3H), 0.88 (s, 3H). **^{13}C{^{1}H} NMR (101 MHz, CDCl$_3$) δ** 166.4, 144.9, 140.1, 134.7, 130.9, 129.9, 129.8, 128.0, 126.6, 80.8, 80.7, 49.2, 47.9, 45.0, 40.6, 37.0, 36.9, 36.2, 28.4, 28.2, 28.1, 27.4, 21.8, 21.7, 19.81, 19.78, 19.0, 13.7, 13.7. **HRMS** (ESI-TOF) *m/z*: $[M + H]^+$ calcd for $C_{26}H_{32}NO_4S$: 454.2047; found: 454.2057.

(*R*)-2-Phenyl-1-tosylaziridine ((*R*)-2a). Tosyl chloride (765 mg, 4.0 mmol) was added slowly to a solution of (*R*)-2-phenylglycinol (**S5**; 250 mg, 1.82 mmol) and K_2CO_3 (1.0 g, 7.3 mmol) in MeCN (17 mL, 0.1 M) and the solution stirred at room temperature overnight (Scheme S4). The reaction mixture

was filtered through Celite and the filtrate was concentrated in vacuo to give a crude residue, which was purified by silica gel column chromatography using EtOAc/petroleum ether (5:95) as eluent to yield (*R*)-2-phenyl-1-tosylaziridine (**(*R*)-2a**; 302 mg, 61%) as a white solid. Spectroscopic data were in agreement with previously reported literature.[7]

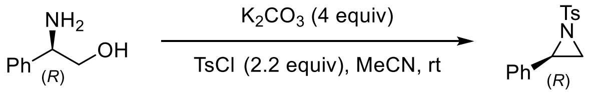

S5 **(*R*)-2a**

Scheme S4

General Procedure D: Reaction of *N*-Benzyltetrahydroquinolines with Aziridines

N-Benzyl tetrahydroquinoline derivative (**1**; 224 mg, 1.0 mmol, 2.0 equiv) and aryl aziridine (**2**; 0.5 mmol, 1.0 equiv) were dissolved in HFIP (2 mL), and the reaction mixture was stirred at room temperature for 16 h. The solvent was then removed under reduced pressure, and the crude product was purified by silica gel column chromatography using EtOAc/petroleum ether (1:9) as eluent.

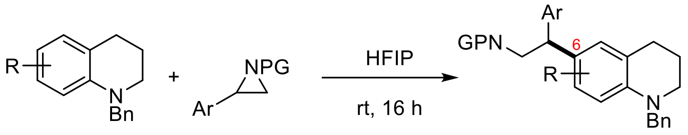

1 **2** **3**

Scheme S5

3. Characterization Data for Compounds

(±)-*N*-(2-(1-Benzyl-1,2,3,4-tetrahydroquinolin-6-yl)-2-phenylethyl)-4-methylbenzenesulfonamide (3aa). Synthesized according to General Procedure D and purified by silica gel chromatography with EtOAc/hexane (1:9). White solid (199 mg, 80%; mp = 140–141 °C). **^{1}H NMR** (400 MHz, $CDCl_3$) δ 7.73–7.67 (m, AA'BB' system, AA' part, 2H), 7.38–7.32 (m, 2H), 7.32–7.24 (m, 7H), 7.22–7.19 (m, 1H), 7.13–7.09 (m, AA'BB' system, BB' part, 2H), 6.71 (d, *J* = 2.0 Hz, 1H), 6.65 (dd, *J* = 8.4, 2.0 Hz, AB system, A part, 1H), 6.39 (d, *J* = 8.4 Hz, AB system, B part, 1H), 4.50–4.40 (m, 3H), 3.91 (t, *J* = 8.0 Hz, 1H), 3.48 (tdd, *J* = 12.6, 10.5, 6.2 Hz, 2H), 3.41–3.32 (m, 2H), 2.75 (t, *J* = 6.3 Hz, 2H), 2.45 (s, 3H), 2.06–1.96 (m, 2H). **^{13}C{^{1}H} NMR (101 MHz, $CDCl_3$) δ** 144.7, 143.4, 141.7, 138.9, 136.8, 129.7, 128.74, 128.71, 128.68, 127.9, 127.3 (2C), 126.9, 126.8, 126.6, 126.5, 122.6, 111.2, 55.3, 50.0, 49.6, 47.5, 28.3, 22.3, 21.6. **HRMS** (ESI-TOF) *m/z*: $[M + H]^+$ calcd for $C_{31}H_{33}N_2O_2S$: 497.2257; found: 497.2271.

(±)-*N*-(2-(1-Benzyl-1,2,3,4-tetrahydroquinolin-6-yl)-2-(2-fluorophenyl)ethyl)-4-methylbenzenesulfonamide (3ab). Synthesized according to General Procedure D and then purified by silica gel chromatography with EtOAc/hexane (1:9). White solid (229 mg, 89%; mp = 84–85 °C). **^{1}H NMR** (400 MHz, $CDCl_3$) δ 7.75–7.66 (m, AA'BB' system, BB' part, 2H), 7.37–7.23 (m, 7H), 7.22–7.13 (m, 1H), 7.09 (t, *J* = 7.3 Hz, 1H), 7.05–6.94 (m, 2H), 6.72 (s, 1H), 6.67 (d, *J* = 8.4 Hz, AB system, A part, 1H), 6.38 (d, *J* = 8.4 Hz, AB system, B part, 1H), 4.52–4.38 (m, 3H), 4.19 (t, *J* = 8.0 Hz, 1H), 3.60–3.50 (m, 1H), 3.50–3.39 (m, 1H), 3.39–3.32 (m, 2H), 2.74 (t, *J* = 6.0 Hz, 2H), 2.44 (s, 3H), 2.06–1.94 (m, 2H). **^{13}C{^{1}H} NMR** (101 MHz, $CDCl_3$) δ 160.6 (d, *J* = 246.0 Hz), 144.8, 143.4, 138.8, 136.8, 129.8, 129.7, 128.9, 128.7, 128.6, 128.3 (d, *J* = 8.2 Hz), 127.2, 126.9, 126.5, 126.1 (d, *J* = 3.4 Hz), 124.3 (d, *J* = 3.2 Hz), 122.5, 115.7 (d, *J* = 22.5 Hz), 111.2, 55.2, 49.9, 46.4, 42.8, 28.2, 22.2, 21.3 (1 signal overlapped in the olefinic region). **^{19}F NMR** (376 MHz, $CDCl_3$) δ -117.07. **HRMS** (ESI-TOF) *m/z*: $[M + H]^+$ calcd for $C_{31}H_{32}FN_2O_2S$: 515.2163; found: 515.2178.

(±)-*N*-(2-(1-Benzyl-1,2,3,4-tetrahydroquinolin-6-yl)-2-(2-chlorophenyl)ethyl)-4-methylbenzenesulfonamide (3ac). Synthesized according to General Procedure D and then purified by silica gel chromatography with EtOAc/hexane (1:9). White solid (215 mg, 81%; mp = 84–85 °C). **^{1}H NMR** (400 MHz, $CDCl_3$) δ 7.77–7.68 (m, AA'BB' system, AA' part, 2H), 7.40–7.25 (m, 8H), 7.21–7.09 (m, 3H), 6.75 (s, 1H), 6.71 (dd, *J* = 8.4, 2.2 Hz, AB system, A part, 1H), 6.42 (d, *J* = 8.4 Hz, AB system, B part, 1H), 4.76–4.63 (m, 1H), 4.55–4.39 (m, 3H), 3.63–3.53 (m, 1H), 3.48–3.40 (m, 1H), 3.40–3.34 (m, 2H), 2.76 (t, *J* = 6.2 Hz, 2H), 2.45 (s, 3H), 2.06–1.97 (m, 2H). **^{13}C{^{1}H} NMR** (101 MHz, $CDCl_3$) δ 145.0, 143.6, 139.4, 139.1, 137.0, 134.5, 130.2, 130.0, 129.2, 128.9, 128.5, 128.1, 127.9, 127.3, 127.1, 127.0, 126.8, 126.1, 122.7, 111.4, 55.5, 50.1, 46.8, 45.8, 28.5, 22.5, 21.8. **HRMS** (ESI-TOF) *m/z*: $[M + H]^+$ calcd for $C_{31}H_{32}ClN_2O_2S$: 531.1868; found: 531.1885.

(±)-*N*-(2-(1-Benzyl-1,2,3,4-tetrahydroquinolin-6-yl)-2-(2-bromophenyl)ethyl)-4-methylbenzenesulfonamide (3ad). Synthesized according to General Procedure D and then purified by silica gel chromatography with EtOAc/hexane (1:9). White solid (225 mg, 78%; mp = 82–83 °C). **^{1}H NMR** (400 MHz, $CDCl_3$) δ 7.78–7.61 (m, AA'BB' system, AA' part, 2H), 7.50 (d, *J* = 7.7 Hz, 1H), 7.37–7.30 (m, 2H), 7.30–7.24 (m, 5H), 7.20 (t, *J* = 7.7 Hz, 1H), 7.12 (dd, *J* = 7.7, 1.7 Hz, 1H), 7.03 (t, *J* = 7.7 Hz, 1H), 6.71 (s, 1H), 6.67 (dd, *J* = 8.4, 2.0 Hz, AB system, A part, 1H), 6.38 (d, *J* = 8.4 Hz, AB system, B part, 1H), 4.53–4.48 (m, 1H), 4.44 (s, 2H), 4.39 (t, *J* = 7.9 Hz, 1H), 3.57–3.49 (m, 1H), 3.43–3.38 (m, 1H), 3.38–3.33 (m, 2H), 2.74 (t, *J* = 6.2 Hz, 2H), 2.43 (s, 3H), 2.03–1.96 (m, 2H). **^{13}C{^{1}H} NMR** (101 MHz, $CDCl_3$) δ 145.1, 144.4, 143.7, 139.0, 136.9, 131.0, 130.4, 130.1, 130.0, 128.9, 128.8, 127.4, 127.1, 126.7, 126.6, 126.55, 126.53, 123.0, 122.9, 111.4, 55.4, 50.1, 49.6, 47.4, 28.4, 22.4, 21.8. **HRMS** (ESI-TOF) *m/z*: $[M + H]^+$ calcd for $C_{31}H_{32}BrN_2O_2S$: 575.1362; found: 575.1385.

(±)-*N*-(2-(1-Benzyl-1,2,3,4-tetrahydroquinolin-6-yl)-2-(*o*-tolyl)ethyl)-4-methylbenzenesulfonamide (3ae). Synthesized according to General Procedure D and then purified by silica gel chromatography with EtOAc/hexane (1:9). Colorless viscous material (192 mg, 75%). **^{1}H NMR** (400 MHz, $CDCl_3$) δ 7.72–7.65 (m, AA'BB' system, AA' part, 2H), 7.35–7.22 (m, 7H), 7.15–7.08 (m, 3H), 7.06–6.99 (m, 1H), 6.65 (d, *J* = 1.7 Hz, 1H), 6.60 (dd, *J* = 8.4, 1.7 Hz, AB system, A part, 1H), 6.35 (d, *J* = 8.4 Hz, AB system, B part, 1H), 4.51–4.26 (m, 3H), 4.07 (t, *J* = 7.9 Hz, 1H), 3.48–3.39 (m, 2H), 3.37–3.29 (m, 2H), 2.71 (t, *J* = 6.3 Hz, 2H), 2.44 (s, 3H), 2.17 (s, 3H), 2.02–1.94 (m, 2H). **^{13}C{^{1}H} NMR** (101 MHz, $CDCl_3$) δ 144.8, 143.6, 139.5, 139.0, 137.0, 131.2, 129.9, 129.0, 128.8, 127.4, 127.1, 127.0, 126.9, 126.8, 126.7, 126.4, 126.0, 122.7, 111.3, 55.5, 50.1, 47.5, 45.6, 28.4, 22.5, 21.7, 19.8. **HRMS** (ESI-TOF) *m/z*: $[M + H]^+$ calcd for $C_{32}H_{35}N_2O_2S$: 511.2414; found: 511.2433.

(±)-*N*-(2-(1-Benzyl-1,2,3,4-tetrahydroquinolin-6-yl)-2-(3-bromophenyl)ethyl)-4-methylbenzenesulfonamide (3af). Synthesized according to General Procedure D and then purified by silica gel chromatography with EtOAc/hexane (1:9). Colorless viscous material (219 mg, 76%). **^{1}H NMR** (400 MHz, $CDCl_3$) δ 7.72–7.62 (m, AA'BB' system, AA' part, 2H), 7.38–7.21 (m, 8H), 7.17 (s, 1H), 7.11 (t, J = 7.8 Hz, 1H), 7.04 (d, J = 7.8 Hz, 1H), 6.65 (d, J = 1.8 Hz, 1H), 6.60 (dd, J = 8.4, 1.8 Hz, AB system, A part, 1H), 6.37 (d, J = 8.4 Hz, AB system, B part, 1H), 4.50–4.33 (m, 3H), 3.84 (t, J = 7.9 Hz, 1H), 3.52–3.23 (m, 4H), 2.73 (t, J = 6.2 Hz, 2H), 2.44 (s, 3H), 2.05–1.87 (m, 2H). **^{13}C{^{1}H} NMR (101 MHz, $CDCl_3$)** δ 145.1, 144.4, 143.7, 139.0, 136.9, 131.0, 130.4, 130.1, 130.0, 128.9, 128.8, 127.4, 127.1, 126.7, 126.6, 126.55, 126.53, 123.0, 122.9, 111.4, 55.4, 50.1, 49.6, 47.4, 28.4, 22.4, 21.8. HRMS (ESI-TOF) *m/z*: $[M + H]^+$ calcd for $C_{31}H_{32}BrN_2O_2S$: 575.1362; found: 575.1364.

(±)-*N*-(2-(1-Benzyl-1,2,3,4-tetrahydroquinolin-6-yl)-2-(*m*-tolyl)ethyl)-4-methylbenzenesulfonamide (3ag). Synthesized according to General Procedure D and then purified by silica gel chromatography with EtOAc/hexane (1:9). Colorless viscous material (205 mg, 80%). **^{1}H NMR** (400 MHz, $CDCl_3$) δ 7.75–7.68 (m, AA'BB' system, AA' part, 2H), 7.39–7.33 (m, 2H), 7.33–7.25 (m, 5H), 7.17 (t, *J* = 7.8 Hz, 1H), 7.03 (d, *J* = 7.5 Hz, 1H), 6.96–6.90 (m, 2H), 6.73 (s, 1H), 6.68 (dd, *J* = 8.4, 1.9 Hz, AB system, A part, 1H), 6.41 (d, *J* = 8.4 Hz, AB system, B part, 1H), 4.59–4.50 (m, 1H), 4.46 (s, 2H), 3.89 (t, *J* = 8.0 Hz, 1H), 3.57–3.43 (m, 2H), 3.41–3.33 (m, 2H), 2.77 (t, *J* = 6.2 Hz, 2H), 2.46 (s, 3H), 2.31 (s, 3H), 2.07–1.96 (m, 2H). **^{13}C{^{1}H} NMR** (101 MHz, $CDCl_3$) δ 144.6, 143.4, 141.6, 138.9, 138.3, 136.8, 129.7, 128.64, 128.63 (2C), 128.59, 127.6, 127.5, 127.2, 126.8, 126.5, 126.3, 124.7, 122.5, 111.2, 55.3, 49.9, 49.5, 47.5, 28.2, 22.3, 21.6, 21.5. **HRMS** (ESI-TOF) *m/z*: $[M + H]^+$ calcd for $C_{32}H_{35}N_2O_2S$: 511.2414; found: 511.2438.

(±)-*N*-(2-(1-Benzyl-1,2,3,4-tetrahydroquinolin-6-yl)-2-(4-chlorophenyl)ethyl)benzenesulfonamide (3ah). Synthesized according to General Procedure D and then purified by silica gel chromatography with EtOAc/hexane (1:9). Colorless viscous material (207 mg, 78%). **^{1}H NMR** (400 MHz, $CDCl_3$) δ 7.72–7.62 (m, AA'BB' system, AA' part, 2H), 7.39–7.30 (m, 2H), 7.31–7.23 (m, 5H), 7.23–7.15 (m, 2H), 7.07–7.00 (m, 2H), 6.66 (d, *J* = 2.1 Hz, 1H), 6.62 (dd, *J* = 8.4, 2.1 Hz, AB system, A part, 1H), 6.38 (d, *J* = 8.4 Hz, AB system, B part, 1H), 4.61–4.50 (m, 1H), 4.45 (s, 2H), 3.88 (t, *J* = 8.0 Hz, 1H), 3.53–3.39 (m, 2H), 3.39–3.33 (m, 2H), 2.74 (t, *J* = 6.3 Hz, 2H), 2.44 (s, 3H), 2.05–1.92 (m, 2H). **^{13}C{^{1}H} NMR** (101 MHz, $CDCl_3$) δ 145.0, 143.7, 140.6, 139.0, 136.9, 132.6, 130.0, 129.4, 129.0, 128.9, 128.8, 127.4, 127.1, 127.0, 126.7, 126.5, 122.8, 111.4, 55.4, 50.1, 49.2, 47.6, 28.4, 22.4, 21.8. **HRMS** (ESI-TOF) *m/z*: $[M + H]^+$ calcd for $C_{31}H_{32}ClN_2O_2S$: 531.1868; found: 531.1847.

(±)-*N*-(2-(1-Benzyl-1,2,3,4-tetrahydroquinolin-6-yl)-2-(4-bromophenyl)ethyl)-4-methylbenzenesulfonamide (3ai). Synthesized according to General Procedure D and then purified by silica gel chromatography with EtOAc/hexane (1:9). White solid (222 mg, 77%; mp = 83–84 °C). **^{1}H NMR** (400 MHz, $CDCl_3$) δ 7.69–7.60 (m, AA'BB' system, AA' part, 2H), 7.37–7.20 (m, 9H), 7.00–6.90 (m, AA'BB' system, BB' part, 2H), 6.63 (s, 1H), 6.59 (dd, *J* = 8.4, 2.0 Hz, AB system, A part, 1H), 6.36 (d, *J* = 8.4 Hz, AB system, B part, 1H), 4.48–4.36 (m, 3H), 3.84 (t, *J* = 7.9 Hz, 1H), 3.52–3.29 (m, 4H), 2.72 (t, *J* = 6.3 Hz, 2H), 2.43 (s, 3H), 2.03–1.94 (m, 2H). **^{13}C NMR** (101 MHz, $CDCl_3$) δ 145.0, 143.7, 141.1, 138.9, 136.9, 131.9, 130.0, 129.7, 128.8, 128.7, 127.4, 127.1, 126.8, 126.7, 126.5, 122.8, 120.8, 111.4, 55.4, 50.1, 49.3, 47.4, 28.4, 22.4, 21.8. **HRMS** (ESI-TOF) *m/z*: $[M + H]^+$ calcd for $C_{31}H_{32}BrN_2O_2S$: 575.1362; found: 575.1387.

(±)-*N*-(2-(1-Benzyl-1,2,3,4-tetrahydroquinolin-6-yl)-2-(p-tolyl)ethyl)-4-methylbenzenesulfonamide (3aj). Synthesized according to General Procedure D and then purified by silica gel chromatography with EtOAc/hexane (1:9). Colorless viscous material (225 mg, 88%). **^{1}H NMR** (400 MHz, $CDCl_3$) δ 7.73–7.63 (m, AA'BB' system, AA' part, 2H), 7.41–7.19 (m, 7H), 7.09–7.05 (m, AA'BB' system, AA' part, 2H), 7.01–6.96 (m, AA'BB' system, BB' part, 2H), 6.69 (s, 1H), 6.63 (dd, *J* = 8.4, 2.1 Hz, AB system, A part, 1H), 6.38 (d, *J* = 8.4 Hz, AB system, B part, 1H), 4.44 (s, 2H), 4.40 (t, *J* = 5.9 Hz, 1H), 3.85 (t, *J* = 8.0 Hz, 1H), 3.54–3.29 (m, 4H), 2.74 (t, *J* = 6.2 Hz, 2H), 2.45 (s, 3H), 2.30 (s, 3H), 2.07–1.93 (m, 2H). **^{13}C{^{1}H} NMR** (101 MHz, $CDCl_3$) δ 144.9, 143.6, 139.0, 138.8, 137.0, 136.6, 129.9, 129.6, 128.86, 128.84, 127.9, 127.7, 127.4, 127.0, 126.7, 126.6, 122.7, 111.4, 55.5, 50.1, 49.4, 47.7, 28.4, 22.5, 21.8, 21.2. **HRMS** (ESI-TOF) *m/z*: $[M + H]^+$ calcd for $C_{32}H_{35}N_2O_2S$: 511.2414; found: 511.2436.

(±)-*N*-(2-([1,1'-Biphenyl]-4-yl)-2-(1-benzyl-1,2,3,4-tetrahydroquinolin-6-yl)ethyl)-4-methylbenzenesulfonamide (3ak). Synthesized according to General Procedure D and then purified by silica gel chromatography with EtOAc/hexane (1:9). White solid (212 mg, 74%; mp = 101–102 °C). **^{1}H NMR** (400 MHz, $CDCl_3$) δ 7.72–7.66 (m, AA'BB' system, AA' part, 2H), 7.55–7.51 (m, AA'BB' system, AA' part, 2H), 7.47 (d, *J* = 8.0 Hz, 2H), 7.42 (t, *J* = 7.7 Hz, 2H), 7.36–7.23 (m, 8H), 7.18–7.13 (m, AA'BB' system, BB' part, 2H), 6.71 (d, *J* = 1.8 Hz, 1H), 6.66 (dd, *J* = 8.4, 1.8 Hz, AB system, A part, 1H), 6.38 (d, *J* = 8.4 Hz, AB system, B part, 1H), 4.44 (s, 2H), 4.43–4.39 (m, 1H), 3.92 (t, *J* = 8.0 Hz, 1H), 3.59–3.40 (m, 2H), 3.40–3.31 (m, 2H), 2.75 (t, *J* = 6.3 Hz, 2H), 2.43 (s, 3H), 2.05–1.95 (m, 2H). **^{13}C{^{1}H} NMR** (101 MHz, $CDCl_3$) δ 145.0, 143.6, 140.9, 139.9, 139.0, 136.9, 129.9, 129.0, 128.9, 128.8, 128.4, 127.6, 127.4, 127.3, 127.2, 127.0, 126.7, 126.6, 122.8, 111.4, 55.4, 50.1, 49.5, 47.6, 28.4, 22.4, 21.8. (*Note: One aromatic carbon signal may be missing due to peak overlap.*) **HRMS** (ESI-TOF) *m/z*: $[M + H]^+$ calcd for $C_{37}H_{37}N_2O_2S$: 573.2570; found: 573.2591.

(±)-Methyl4-(1-(1-benzyl-1,2,3,4-tetrahydroquinolin-6-yl)-2-((4-methylphenyl)sulfonamido)ethyl)benzoate (3al). Synthesized according to General Procedure D at 80 °C, purified by silica gel chromatography with EtOAc/hexane (1:9). White solid (219 mg, 79%; mp = 164–165 °C). **^{1}H NMR (400 MHz, $CDCl_3$) δ** 7.92 – 7.87 (m, AA'BB' system, AA' part, 2H), 7.70 – 7.64 (m, AA'BB' system, AA' part, 2H), 7.35 – 7.30 (m, 2H), 7.29 – 7.21 (m, 5H), 7.19 – 7.13 (m, AA'BB' system, BB' part, 2H), 6.66 (s, 1H), 6.63 (d, *J* = 8.4 Hz, AB system, A part, 1H), 6.39 (d, *J* = 7.5 Hz, AB system, B part, 1H), 4.51 (t, *J* = 5.7 Hz, 1H), 4.43 (s, 2H), 3.95 (t, *J* = 7.9 Hz, 1H), 3.89 (s, 3H), 3.56 – 3.48 (m, 1H), 3.46 – 3.39 (m, 1H), 3.38 – 3.33 (m, 2H), 2.72 (t, *J* = 6.3 Hz, 2H), 2.43 (s, 3H), 2.02 – 1.95 (m, 2H). **^{13}C{^{1}H} NMR (101 MHz, $CDCl_3$) δ** 166.9, 147.2, 144.8, 143.6, 138.6, 136.8, 130.0, 129.9, 129.8, 128.74 (2C), 128.68, 127.9, 127.2, 127.0, 126.7, 126.5, 122.9, 111.5, 55.4, 52.2, 49.9, 49.8, 47.2, 28.2, 22.2, 21.6. **HRMS** (ESI-TOF) *m/z*: $[M + H]^+$ calcd for $C_{33}H_{35}N_2O_4S$: 555.2312; found: 555.2320.

(±)-*N*-(2-(1-Benzyl-1,2,3,4-tetrahydroquinolin-6-yl)-2-(naphthalen-1-yl)ethyl)-4-methylbenzenesulfonamide (3ao). Synthesized according to General Procedure D and then purified by silica gel chromatography with EtOAc/hexane (1:9). White solid (232 mg, 85%; mp = 84–85 °C). **^{1}H NMR** (400 MHz, $CDCl_3$) **δ** 7.87–7.63 (m, 5H), 7.54 (s, 1H), 7.52–7.41 (m, 2H), 7.41–7.31 (m, 2H), 7.30–7.20 (m, 6H), 6.75 (s, 1H), 6.71 (dd, *J* = 8.4, 1.9 Hz, AB system, A part, 1H), 6.41 (d, *J* = 8.4 Hz, AB system, B part, 1H), 4.56–4.48 (m, 1H), 4.45 (s, 2H), 4.08 (t, *J* = 7.9 Hz, 1H), 3.72–3.47 (m, 2H), 3.45–3.21 (m, 2H), 2.75 (t, *J* = 6.2 Hz, 2H), 2.45 (s, 3H), 2.09–1.90 (m, 2H). **^{13}C{^{1}H} NMR** (101 MHz, $CDCl_3$) **δ** 145.0, 143.7, 139.3, 139.0, 136.9, 133.6, 132.6, 130.0, 129.0, 128.9, 128.7, 128.0, 127.8, 127.5, 127.4, 127.1, 126.8, 126.7, 126.6, 126.4, 126.2, 126.0, 122.8, 111.4, 55.5, 50.1,

49.8, 47.6, 28.4, 22.5, 21.8. **HRMS** (ESI-TOF) *m/z*: $[M + H]^+$ calcd for $C_{35}H_{35}N_2O_2S$: 547.2414; found: 547.2441.

(±)-*N*-(2-(1-Benzyl-1,2,3,4-tetrahydroquinolin-6-yl)-2-phenylethyl)benzenesulfonamide (3ap). Synthesized according to General Procedure D and then purified by silica gel chromatography with EtOAc/hexane (1:9). Colorless viscous material (191 mg, 79%). **^{1}H NMR** (400 MHz, $CDCl_3$) δ 7.86–7.80 (m, AA'BB' system, AA' part, 2H), 7.63–7.57 (m, 1H), 7.50 (t, *J* = 7.7 Hz, 2H), 7.40–7.32 (m, 2H), 7.31–7.24 (m, 5H), 7.23–7.17 (m, 1H), 7.13 (d, *J* = 7.5 Hz, 2H), 6.72 (s, 1H), 6.67 (dd, *J* = 8.4, 1.5 Hz, 1H), 6.41 (d, *J* = 8.4 Hz, 1H), 4.61 (t, *J* = 5.9 Hz, NH, 1H), 4.46 (s, CH_2, 2H), 3.92 (t, *J* = 8.0 Hz, 1H), 3.63–3.43 (m, 2H), 3.43–3.32 (m, 2H), 2.76 (t, *J* = 6.2 Hz, 2H), 2.08–1.94 (m, 2H). **^{13}C{^{1}H} NMR** (101 MHz, $CDCl_3$) δ 144.7, 141.7, 139.7, 138.8, 132.7, 129.1, 128.7, 128.6 (2C), 127.8, 127.3, 127.1, 126.9, 126.8, 126.5, 126.4, 122.5, 111.2, 55.2, 49.9, 49.6, 47.5, 28.2, 22.3. **HRMS** (ESI-TOF) *m/z*: $[M + H]^+$ calcd for $C_{30}H_{31}N_2O_2S$: 483.2101; found: 483.2114.

(±)-*N*-(2-(1-Benzyl-1,2,3,4-tetrahydroquinolin-6-yl)-2-phenylethyl)-4-nitrobenzenesulfonamide (3aq). Synthesized according to General Procedure D and then purified by silica gel chromatography with EtOAc/hexane (1:4). Orange solid (219 mg, 83%; mp = 127–128 °C). **^{1}H NMR** (400 MHz, $CDCl_3$) δ 8.31–8.24 (m, AA'BB' system, AA' part, 2H), 7.96–7.88 (m, BB' part, 2H), 7.38–7.31 (m, 2H), 7.30–7.24 (m, 5H), 7.23–7.17 (m, 1H), 7.12 (d, *J* = 7.5 Hz, 2H), 6.71 (s, 1H), 6.67 (d, *J* = 8.4 Hz, AB system, A part, 1H), 6.38 (d, *J* = 8.4 Hz, AB system, B part, 1H), 4.81 (t, *J* = 5.6 Hz, 1H), 4.45 (s, 2H), 3.93 (t, *J* = 8.0 Hz, 1H), 3.72–3.45 (m, 2H), 3.42–3.31 (m, 2H), 2.74 (t, *J* = 6.2 Hz, 2H), 2.06–1.94 (m, 2H). **^{13}C{^{1}H} NMR** (101 MHz, $CDCl_3$) δ 149.9, 145.8, 144.8, 141.4, 138.7, 128.8, 128.7, 128.6, 128.3, 127.8, 127.0, 126.9, 126.6, 126.3, 124.3, 122.7, 111.2, 55.2, 49.95, 49.90, 47.7, 28.2, 22.2 (1 signal overlapped in the olefinic region). **HRMS** (ESI-TOF) *m/z*: $[M + H]^+$ calcd for $C_{30}H_{30}N_3O_4S$: 528.1952; found: 528.1973.

(±)-*N*-(2-(1-Benzyl-1,2,3,4-tetrahydroquinolin-6-yl)-2-phenylethyl)-4-chlorobenzenesulfonamide (3ar). Synthesized according to General Procedure D and then purified by silica gel chromatography with EtOAc/hexane (1:9). Colorless viscous material (163 mg, 63%). **^{1}H NMR (400 MHz, $CDCl_3$) δ** 7.74–7.67 (m, AA'BB' system, AA' part, 2H), 7.47–7.41 (m, BB' part, 2H), 7.36–7.31 (m, 2H), 7.29–7.23 (m, 5H), 7.22–7.17 (m, 1H), 7.10 (d, *J* = 6.9 Hz, 2H), 6.69 (s, 1H), 6.64 (d, *J* = 7.6 Hz, AB system, A part, 1H), 6.39 (d, *J* = 7.6 Hz, AB system, B part, 1H), 4.49 (br s, NH, 1H), 4.44 (s, 2H), 3.90 (t, *J* = 8.0 Hz, 1H), 3.56–3.40 (m, 2H), 3.39–3.32 (m, 2H), 2.74 (t, *J* = 6.2 Hz, 2H), 2.04–1.96 (m, 2H). **^{13}C{^{1}H} NMR** (101 MHz, $CDCl_3$) δ 144.7, 141.6, 139.2, 138.8, 138.4, 129.4, 128.9, 128.8, 128.7 (2C), 127.8, 127.02, 126.97, 126.7, 126.4, 122.8, 111.3, 55.4, 50.0, 49.7, 47.6, 28.2, 22.2 (1 signal

overlapped in the olefinic region). **HRMS** (ESI-TOF) *m/z*: $[M + H]^+$ calcd for $C_{30}H_{30}ClN_2O_2S$: 517.1711; found: 517.1713.

(±)-*N*-(2-(1-Benzyl-5-chloro-1,2,3,4-tetrahydroquinolin-6-yl)-2-phenylethyl)-4-methylbenzenesulfonamide (3ba). Synthesized according to General Procedure D and then purified by silica gel chromatography with EtOAc/hexane (1:9). Colorless oil (207 mg, 78%). **^{1}H NMR** (400 MHz, $CDCl_3$) δ 7.72–7.61 (m, 2H, AA'BB' system, AA' part), 7.35 (t, *J* = 7.4 Hz, 2H), 7.30–7.22 (m, 7H), 7.20 (d, *J* = 7.4 Hz, 1H), 7.09 (d, *J* = 7.3 Hz, 2H), 6.62 (d, *J* = 8.7 Hz, 1H, AB system, A part), 6.32 (d, *J* = 8.7 Hz, 1H, AB system, B part), 4.50 (t, *J* = 7.9 Hz, 1H), 4.45 (s, 2H), 4.42 (t, *J* = 5.9 Hz, 1H), 3.49–3.43 (m, 2H), 3.39–3.31 (m, 2H), 2.94–2.75 (m, 2H), 2.43 (s, 3H), 2.08–1.96 (m, 2H). **^{13}C{^{1}H} NMR** (101 MHz, $CDCl_3$) δ 145.9, 143.5, 140.7, 138.4, 136.6, 134.9, 129.8, 128.8, 128.7, 128.2, 127.3, 127.1, 126.9, 126.5, 125.8, 124.9, 120.9, 109.8, 55.7, 49.6, 46.7, 46.2, 26.4, 22.0, 21.7. **HRMS** (ESI-TOF) *m/z*: $[M + H]^+$ calcd for $C_{31}H_{32}ClN_2O_2S$: 531.1868; found 531.1862.

(±)-*N*-(2-(1-Benzyl-5-bromo-1,2,3,4-tetrahydroquinolin-6-yl)-2-phenylethyl)-4-methylbenzenesulfonamide (3ca). Synthesized according to General Procedure D and then purified by silica gel chromatography with EtOAc/hexane (1:9). Light yellow solid (204 mg, 71%; mp 74–75 °C). **^{1}H NMR** (400 MHz, $CDCl_3$) δ 7.74–7.66 (m, AA'BB' system, AA' part, 2H), 7.36 (t, *J* = 7.3 Hz, 2H), 7.31–7.23 (m, 7H), 7.20 (d, *J* = 7.0 Hz, 1H), 7.13 (d, *J* = 8.0 Hz, 2H), 6.63 (d, *J* = 8.7 Hz, AB system, A part, 1H), 6.37 (d, *J* = 8.7 Hz, AB system, B part, 1H), 4.58 (t, *J* = 7.9 Hz, 1H), 4.54 (t, *J* = 5.8 Hz, 1H), 4.46 (s, 2H), 3.50–3.43 (m, 2H), 3.40–3.30 (m, 2H), 2.96–2.77 (m, 2H), 2.43 (s, 3H), 2.10–1.95 (m, 2H). **^{13}C{^{1}H} NMR** (101 MHz, $CDCl_3$) δ 146.1, 143.4, 140.8, 138.4, 136.7, 129.7, 128.9, 128.8, 128.6, 128.2, 127.3, 127.0, 126.8, 126.6, 126.4, 126.1, 122.4, 110.4, 55.6, 49.6, 48.8, 46.9, 29.9, 22.3, 21.6. **HRMS** (ESI-TOF) *m/z*: $[M + H]^+$ calcd for $C_{31}H_{32}BrN_2O_2S$: 577.1347; found 577.1350.

(±)-*N*-(2-(1-Benzyl-5-methyl-1,2,3,4-tetrahydroquinolin-6-yl)-2-phenylethyl)-4-methylbenzenesulfonamide (3da). Synthesized according to General Procedure D and then purified by silica gel chromatography with EtOAc/hexane (1:9). Colorless oil (189 mg, 74%). **^{1}H NMR** (400 MHz, $CDCl_3$) δ 7.84–7.74 (m, AA'BB' system, AA' part, 2H), 7.51–7.45 (m, 2H), 7.44–7.38 (m, 3H), 7.37–7.32 (m, 4H), 7.29 (d, *J* = 6.9 Hz, 1H), 7.14 (d, *J* = 7.1 Hz, 2H), 6.71 (d, *J* = 8.6 Hz, AB system, B part, 1H), 6.45 (d, *J* = 8.6 Hz, AB system, B part, 1H), 4.72 (s, 1H), 4.64–4.50 (m, 2H), 4.39 (t, *J* = 7.8 Hz, 1H), 3.64–3.56 (m, 1H), 3.54–3.49 (m, 1H), 3.49–3.39 (m, 3H), 2.89–2.72 (m, 2H), 2.53 (s, 3H), 2.20–2.09 (m, 5H). **^{13}C{^{1}H} NMR** (101 MHz, $CDCl_3$) δ 144.7, 143.3, 141.7, 139.1, 136.6, 135.3, 129.6, 128.6, 128.5, 128.1, 127.1, 126.8, 126.6, 126.4, 125.5, 124.5, 121.6, 109.2, 55.8, 49.4, 47.4,

46.1, 25.7, 22.5, 21.5, 14.9. **HRMS** (ESI-TOF) *m/z*: $[M + H]^+$ calcd for $C_{32}H_{35}N_2O_2S$: 511.2414; found 511.2414.

(±)-*N*-(2-(1-Benzyl-5-methoxy-1,2,3,4-tetrahydroquinolin-6-yl)-2-phenylethyl)-4-methylbenzenesulfonamide (3ea). Synthesized according to General Procedure D and then purified by silica gel chromatography with EtOAc/hexane (1:9). White solid (166 mg, 63%; mp 70–71 °C). **^{1}H NMR** (400 MHz, $CDCl_3$) δ 7.82–7.68 (m, AA'BB' system, AA' part, 2H), 7.43 (d, *J* = 6.8 Hz, 2H), 7.39–7.29 (m, 7H), 7.26 (d, *J* = 7.2 Hz, 1H), 7.14 (d, *J* = 7.2 Hz, 2H), 6.66 (d, *J* = 8.6 Hz, AB system, A part, 1H), 6.30 (d, *J* = 8.6 Hz, AB system, B part, 1H), 4.67 (t, *J* = 5.7 Hz, 1H), 4.61–4.51 (m, 2H), 4.49 (t, *J* = 8.0 Hz, 1H), 3.64 (s, 3H), 3.60–3.54 (m, 1H), 3.50–3.41 (m, 3H), 3.00–2.91 (m, 1H), 2.89–2.80 (m, 1H), 2.50 (s, 3H), 2.13–2.03 (m, 2H). **^{13}C{^{1}H} NMR** (101 MHz, $CDCl_3$) δ 156.5, 146.0, 143.3, 142.0, 138.9, 136.7, 129.7, 128.7, 128.6, 128.0, 127.3, 127.0, 126.7, 126.5, 125.6, 120.1, 115.9, 107.4, 60.6, 55.6, 49.7, 47.4, 43.0, 22.2, 21.9, 21.6. **HRMS** (ESI-TOF) *m/z*: $[M + H]^+$ calcd for $C_{32}H_{35}N_2O_3S$: 527.2363; found 527.2368.

(±)-*N*-(2-(1-Benzyl-7-chloro-1,2,3,4-tetrahydroquinolin-6-yl)-2-phenylethyl)-4-methylbenzenesulfonamide (3fa). Synthesized according to General Procedure D and then purified by silica gel chromatography with EtOAc/hexane (1:9). Off-white solid (202 mg, 76%; mp 156–157 °C). **^{1}H NMR** (400 MHz, $CDCl_3$) δ 7.84–7.74 (m, AA'BB' system, AA' part, 2H), 7.45–7.27 (m, 10H), 7.12 (d, *J* = 7.5 Hz, 2H), 6.73 (s, 1H), 6.55 (s, 1H), 4.56 (t, *J* = 5.9 Hz, 1H), 4.50 (s, 2H), 4.47 (t, *J* = 8.1 Hz, 1H), 3.62–3.56 (m, 2H), 3.45–3.35 (m, 2H), 2.75 (t, *J* = 6.1 Hz, 2H), 2.52 (s, 3H), 2.08–2.00 (m, 2H). **^{13}C{^{1}H} NMR** (101 MHz, $CDCl_3$) δ 145.4, 143.5, 140.7, 138.0, 136.9, 132.8, 129.8, 128.8, 128.7, 128.1 (2C), 127.3, 127.1, 126.9, 126.6, 124.1, 121.4, 111.5, 55.0, 49.4, 46.6, 45.5, 27.9, 22.1, 21.6. **HRMS** (ESI-TOF) *m/z*: $[M + H]^+$ calcd for $C_{31}H_{32}ClN_2O_2S$: 531.1868; found 531.1867.

(±)-*N*-(2-(1-Benzyl-7-bromo-1,2,3,4-tetrahydroquinolin-6-yl)-2-phenylethyl)-4-methylbenzenesulfonamide (3ga). Synthesized according to General Procedure D and then purified by silica gel chromatography with EtOAc/hexane (1:9). White solid (236 mg, 82%; mp 77–78 °C). **^{1}H NMR** (400 MHz, $CDCl_3$) δ 7.74–7.68 (m, AA'BB' system, AA' part, 2H), 7.37–7.18 (m, 10H), 7.12 (d, *J* = 7.4 Hz, 2H), 6.66 (s, 1H), 6.63 (s, 1H), 4.47–4.39 (m, 3H), 4.37 (t, *J* = 7.9 Hz, 1H), 3.54–3.44 (m, 2H), 3.36–3.25 (m, 2H), 2.64 (t, *J* = 6.1 Hz, 2H), 2.44 (s, 3H), 2.00–1.90 (m, 2H). **^{13}C{^{1}H} NMR** (101 MHz, $CDCl_3$) δ 145.6, 143.5, 140.7, 137.9, 136.8, 129.8, 128.83, 128.76, 128.1 (2C), 127.3, 127.2, 127.0, 126.6, 125.7, 123.6, 122.1, 114.7, 54.9, 49.4, 47.9, 46.8, 28.0, 22.0, 21.7. **HRMS** (ESI-TOF) *m/z*: $[M + H]^+$ calcd for $C_{31}H_{32}BrN_2O_2S$: 577.1347; found 577.1346.

(±)-*N*-(2-(1-Benzyl-7-methyl-1,2,3,4-tetrahydroquinolin-6-yl)-2-phenylethyl)-4-methylbenzenesulfonamide (3ha). Synthesized according to General Procedure D and then purified by silica gel chromatography with EtOAc/hexane (1:9). Colorless oil (184 mg, 72%). **^{1}H NMR** (400 MHz, $CDCl_3$) δ 7.83–7.66 (m, AA'BB' system, AA' part, 2H), 7.44–7.28 (m, 9H), 7.27–7.22 (m, 1H), 7.08 (d, *J* = 7.2 Hz, 2H), 6.68 (s, 1H), 6.39 (s, 1H), 4.71–4.41 (m, 3H), 4.18 (t, *J* = 7.9 Hz, 1H), 3.63–3.54 (m, 1H), 3.53–3.45 (m, 1H), 3.44–3.32 (m, 2H), 2.78 (t, *J* = 6.1 Hz, 2H), 2.52 (s, 3H), 2.14–1.95 (m, 5H). **^{13}C{^{1}H} NMR** (101 MHz, $CDCl_3$) δ 144.5, 143.5, 141.7, 139.0, 137.0, 135.7, 129.8, 128.70, 128.68 (2C), 128.1, 127.3, 126.9, 126.8, 126.7, 125.2, 120.0, 113.5, 55.2, 49.7, 47.3, 45.6, 28.0, 22.5, 21.6, 19.7. **HRMS** (ESI-TOF) *m/z*: $[M + H]^+$ calcd for $C_{32}H_{35}N_2O_2S$: 511.2414; found 511.2415.

(±)-*N*-(2-(1-Benzyl-7-methoxy-1,2,3,4-tetrahydroquinolin-6-yl)-2-phenylethyl)-4-methylbenzenesulfonamide (3ia). Synthesized according to General Procedure D and then purified by silica gel chromatography with EtOAc/hexane (1:9). White solid (179 mg, 68%; mp = 78–79 °C). **^{1}H NMR** (400 MHz, $CDCl_3$) δ 7.70–7.64 (m, AA'BB' system, AA' part, 2H), 7.37–7.21 (m, 9H), 7.20–7.15 (m, 1H), 7.11 (d, *J* = 6.9 Hz, 2H), 6.45 (s, 1H), 6.01 (s, 1H), 4.50–4.40 (m, 3H), 4.29 (t, *J* = 8.0 Hz, 1H), 3.54–3.44 (m, 5H), 3.37–3.31 (m, 2H), 2.62 (t, *J* = 6.3 Hz, 2H), 2.44 (s, 3H), 2.01–1.92 (m, 2H). **^{13}C{^{1}H} NMR** (101 MHz, $CDCl_3$) δ 156.5, 145.5, 143.2, 141.7, 139.0, 136.8, 129.6, 128.7, 128.5, 128.4, 128.1, 127.2, 127.0, 126.6, 126.5, 115.6, 114.2, 95.3, 55.7, 55.3, 50.1, 46.5, 42.9, 27.4, 22.5, 21.6. **HRMS** (ESI-TOF) *m/z*: $[M + H]^+$ calcd for $C_{32}H_{35}N_2O_3S$: 527.2363; found 527.2363.

(±)-*N*-(2-(1-Benzyl-8-fluoro-1,2,3,4-tetrahydroquinolin-6-yl)-2-phenylethyl)-4-methylbenzenesulfonamide (3ja). Synthesized according to General Procedure D and then purified by silica gel chromatography with EtOAc/hexane (5:95). Colorless oil (105 mg, 41%). **^{1}H NMR** (400 MHz, $CDCl_3$) δ 7.74–7.65 (m, AA'BB' system, AA' part, 2H), 7.42–7.24 (m, 9H), 7.22 (t, *J* = 7.3 Hz, 1H), 7.10 (d, *J* = 7.3 Hz, 2H), 6.63–6.47 (m, 2H), 4.46 (t, *J* = 6.0 Hz, 1H), 4.33 (s, 2H), 3.93 (t, *J* = 8.0 Hz, 1H), 3.59–3.38 (m, 2H), 3.12–3.00 (m, 2H), 2.68 (t, *J* = 6.3 Hz, 2H), 2.45 (s, 3H), 1.89–1.72 (m, 2H). **^{13}C{^{1}H} NMR** (101 MHz, $CDCl_3$) δ 154.0 (d, *J* = 243.8 Hz), 143.7, 140.94, 139.5 (d, *J* = 1.9 Hz), 136.8, 134.6, 131.3 (d, J = 4.3 Hz), 129.9, 129.5 (d, *J* = 3.0 Hz), 129.0, 128.5, 128.0, 127.9, 127.3, 127.2, 127.1, 124.4 (d, *J* = 2.3 Hz), 113.5 (d, *J* = 22.2 Hz), 58.5 (d, *J* = 8.9 Hz), 49.7, 48.6, 47.4, 28.2 (d, *J* = 2.3 Hz), 21.7, 19.9. **^{19}F NMR** (376 MHz, $CDCl_3$) δ -123.66. **HRMS** (ESI-TOF) *m/z*: $[M + H]^+$ calcd for $C_{31}H_{32}FN_2O_2S$: 515.2163; found 515.2157.

(±)-*N*-(2-(1-Benzyl-2-methyl-1,2,3,4-tetrahydroquinolin-6-yl)-2-phenylethyl)-4-methylbenzenesulfonamide (1:1 dr) (3na). Synthesized according to General Procedure D and then purified by silica gel chromatography with EtOAc/hexane (1:9). Colorless oil (198 mg, 75%). **^{1}H NMR** (400 MHz, $CDCl_3$) δ 7.71–7.68 (m, AA'BB' system, AA' part, 2H), 7.69–7.65 (m, AA'BB' system, AA' part, 2H), 7.36–7.22 (m, 18H), 7.22–7.16 (m, 2H), 7.09 (d, *J* = 7.4 Hz, 4H), 6.72 (s, 2H),

6.63–6.57 (m, 2H), 6.28 (d, *J* = 6.0 Hz, 2H), 4.55–4.41 (m, 4H), 4.40–4.34 (m, 2H), 3.88 (t, *J* = 8.0 Hz, 2H), 3.63–3.55 (m, 2H), 3.54–3.38 (m, 4H), 2.91–2.80 (m, 2H), 2.72–2.62 (m, 1H), 2.45 (s, 3H), 2.44 (s, 3H), 1.19 (d, *J* = 6.4 Hz, 3H), 1.18 (d, J = 6.4 Hz, 3H). **$^{13}C\{^1H\}$ NMR (101 MHz, $CDCl_3$) δ** 143.9, 143.5, 141.8, 139.4, 136.9, 136.8, 129.8, 128.8, 128.7, 128.5, 127.92, 127.90, 127.3, 126.9, 126.8, 126.5, 126.4, 126.3, 122.2, 111.7, 53.5, 53.1, 49.7, 47.6, 28.1, 24.2, 24.1, 21.6, 19.1, 19.0 (other signals overlapped). **HRMS** (ESI-TOF) *m/z*: $[M + H]^+$ calcd for $C_{32}H_{35}N_2O_2S$: 511.2414; found 511.2417.

(±)-4-Methyl-*N*-(2-phenyl-2-(2,3,6,7-tetrahydro-1*H*,5*H*-pyrido[3,2,1-*ij*]quinolin-9-yl)ethyl)benzenesulfonamide (3oa). Synthesized according to General Procedure D and then purified by silica gel chromatography with EtOAc/hexane (1:9). Colorless oil (156 mg, 70%). **^{1}H NMR** (400 MHz, $CDCl_3$) δ 7.73–7.66 (m, AA’BB’ system, AA’ part, 2H), 7.34–7.29 (m, AA’BB’ system, BB’ part, 2H), 7.28–7.22 (m, 2H), 7.20–7.15 (m, 1H), 7.10 (d, *J* = 7.1 Hz, 2H), 6.47 (s, 2H), 4.47 (t, *J* = 5.6 Hz, 1H), 3.84 (t, *J* = 8.0 Hz, 1H), 3.55–3.37 (m, 2H), 3.15–3.05 (m, 4H), 2.65 (t, *J* = 6.5 Hz, 4H), 2.45 (s, 3H), 2.00–1.88 (m, 4H). **$^{13}C\{^1H\}$ NMR** (101 MHz, $CDCl_3$) δ 143.43, 142.08, 141.86, 136.93, 129.76, 128.74, 127.88, 127.29, 127.23, 126.77, 126.44, 121.87, 50.00, 49.71, 47.54, 27.74, 22.07, 21.64. **HRMS** (ESI-TOF) *m/z*: $[M + H]^+$ calcd for $C_{27}H_{31}N_2O_2S$: 447.2101; found 447.2094.

***(R)*-*N*-(2-(1-Benzyl-1,2,3,4-tetrahydroquinolin-6-yl)-2-phenylethyl)-4-methylbenzenesulfonamide ((*R*)-3aa).** Synthesized according to General Procedure D and then purified by silica gel chromatography with EtOAc/hexane (1:9). White solid (199 mg, 80%; mp = 129–130 °C). Optical Rotation: [α]25 D = -0.16 (c = 1, CH_2Cl_2). >96% ee, HPLC condition: Chiralpak AD-H column, *n*-hexane/*i*-PrOH = 90:10, flow rate = 1 ml/min, wavelength = 254 nm, tR = 10.7 min for minor isomer, tR = 12.2 min for major isomer).

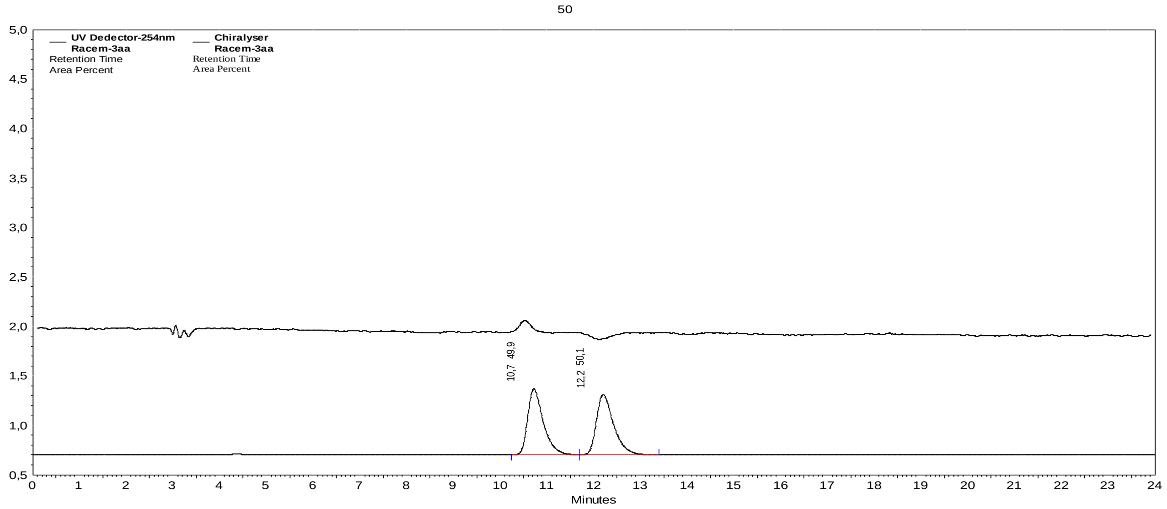

Figure S1: HPLC chromatogram of racemic compound **3aa** (AD-H column; 90:10 hexane−isopropanol; 1.0 mL min−1)

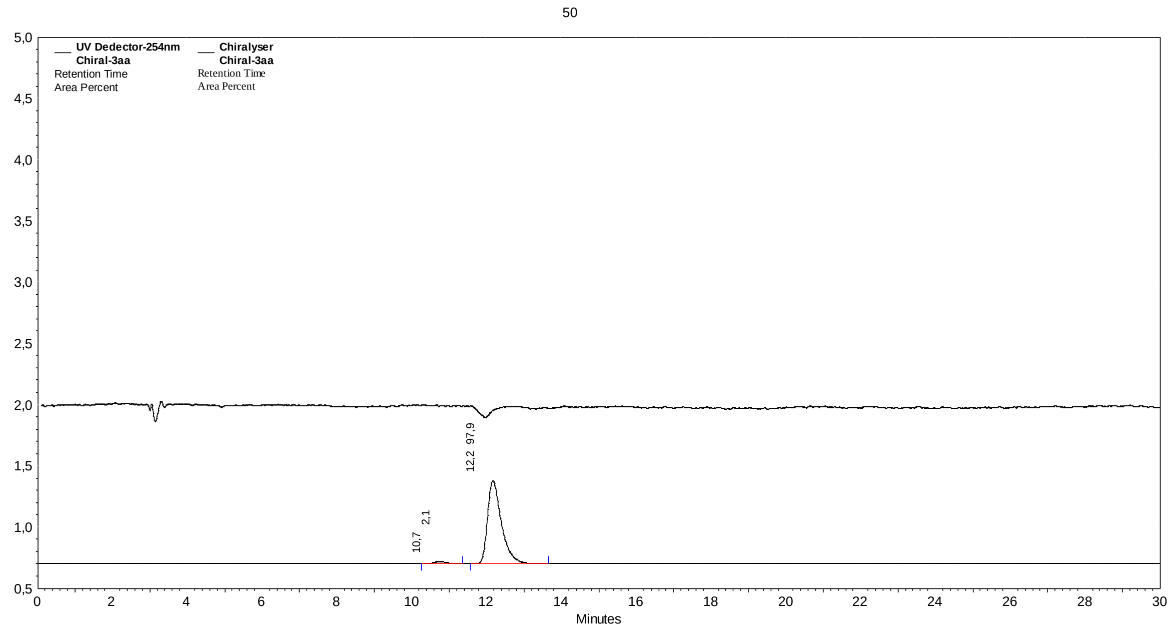

Figure S2: HPLC chromatogram of chiral compound **(*R*)-3aa** (>96% ee; AD-H column; 90:10 hexane−isopropanol; 1.0 mL min−1)

(±)-4-Methyl-*N*-(2-phenyl-2-(1,2,3,4-tetrahydroquinolin-6-yl)ethyl)benzenesulfonamide (4)

About 10% Pd/C (10 mg) was added to a solution of the *N*-(2-(1-benzyl-1,2,3,4-tetrahydroquinolin-6-yl)-2-phenylethyl)-4-methylbenzenesulfonamide **3aa** (100 mg, 0.2 mmol) in MeOH/DCM (2/1) (10 mL). The system was degassed 3 times (vacuum/H_2), then reacted under H_2 atmosphere (1 atm) for 12 hours at room temperature. The mixture was filtered over a filter paper, and the filtrate was concentrated on rotary evaporator to provide the product **4** as a yellow solid (77 mg, 95%; mp = 81–82 °C). **^{1}H NMR (400 MHz, $CDCl_3$) δ** 7.72 – 7.66 (m, AA'BB' system, AA' part, 2H), 7.33 – 7.28 (m, AA'BB' system, BB' part, 2H), 7.25 (t, *J* = 7.4 Hz, 2H), 7.19 (t, *J* = 7.4 Hz, 1H), 7.10 (d, *J* = 7.4 Hz, 2H), 6.71 – 6.61 (m, 2H), 6.37 (d, *J* = 7.8 Hz, AB system, B part, 1H), 4.55 (t, *J* = 5.9 Hz, 1H), 3.90 (t, *J* = 8.0 Hz, 1H), 3.72 (bs, 1H), 3.56 – 3.40 (m, 2H), 3.30 – 3.18 (m, 2H), 2.66 (t, *J* = 6.4 Hz, 2H), 2.45 (s, 3H), 1.94 – 1.84 (m, 2H). **^{13}C{^{1}H} NMR (101 MHz, $CDCl_3$) δ** 143.7, 143.4, 141.7, 136.8, 129.7, 129.1, 128.70, 128.66, 127.8, 127.2, 126.8, 126.2, 121.8, 114.6, 49.7, 47.5, 41.9, 27.0, 22.0, 21.6. **HRMS (ESI-TOF)** *m/z*: $[M + H]^+$ calcd for $C_{24}H_{27}N_2O_2S$: 407.1788; found 407.1795.

(±)-4-Methyl-*N*-(2-phenyl-2-(quinolin-6-yl)ethyl)benzenesulfonamide (5)

To a solution of 4-Methyl-*N*-(2-phenyl-2-(1,2,3,4-tetrahydroquinolin-6-yl)ethyl)benzenesulfonamide (**4**; 50mg, 0.125 mmol) in CH_2Cl_2 (10 mL) was added the active MnO_2 (130 mg, 1.5mmol). The mixture was stirred at rt for 12 h. The reaction was monitored by TLC. After filtration, the mixture was evaporated under reduced pressure, and the compound **5** was purified by silica gel column chromatography using EtOAc/hexane (60:40). 4-Methyl-*N*-(2-phenyl-2-(quinolin-6-

yl)ethyl)benzenesulfonamide (**5**) was obtained as white solid (43 mg, 85%; mp = 204–205 °C). **^{1}H NMR (400 MHz, CDCl$_3$) δ** 8.87 – 8.78 (m, 1H), 8.03 (d, *J* = 8.4 Hz, 1H), 7.92 (d, *J* = 8.8 Hz, 1H), 7.70 – 7.65 (m, AA'BB' system, BB' part, 2H), 7.57 (s, 1H), 7.41 (dd, *J* = 8.7, 1.8 Hz, 1H), 7.34 (dd, *J* = 8.2, 4.3 Hz, 1H), 7.30 – 7.19 (m, 5H), 7.17 – 7.11 (m, 2H), 5.13 (t, *J* = 5.8 Hz, 1H), 4.31 (t, *J* = 7.9 Hz, 1H), 3.74 – 3.57 (m, 2H), 2.41 (s, 3H). **^{13}C{^{1}H} NMR (101 MHz, CDCl$_3$) δ** 150.0, 146.7, 143.6, 140.5, 139.7, 136.9, 136.6, 130.3, 129.8, 129.6, 129.1, 128.2, 128.1, 127.4, 127.2, 126.5, 121.4, 50.7, 47.2, 21.7. **HRMS (ESI-TOF)** *m/z*: [M + H]$^+$ calcd for $C_{24}H_{23}N_2O_2S$: 403.1475; found 403.1493.

1-Benzyl-6-(1-tosylindolin-3-yl)-1,2,3,4-tetrahydroquinoline (6). To a stirred solution of *N*-(2-(1-benzyl-1,2,3,4-tetrahydroquinolin-6-yl)-2-(2-bromophenyl)ethyl)-4-methylbenzenesulfonamide (**3ad**; 0.1 mmol, 58 mg), 1,2-diaminocyclohexane (2.3 mg, 20 mol %), and K_2CO_3 (21 mg, 0.15 mmol) in DMF (2 mL), CuI (2 mg, 10 mol %) was added at room temperature and then stirred at 120 °C for 4 h in an oil bath. After completion, the reaction mixture was cooled to room temperature and diluted with EtOAc (15 mL). The mixture was successively washed with brine (2 × 5 mL). Drying with Na_2SO_4 and evaporation of the solvent provided a residue that was purified by silica gel column chromatography using EtOAc/hexane (20:80) to give 1-benzyl-6-(1-tosylindolin-3-yl)-1,2,3,4-tetrahydroquinoline (**6**) as a colorless liquid in 82% yield (40 mg). **^{1}H NMR (400 MHz, CDCl$_3$) δ** 7.76 – 7.68 (m, 3H), 7.37 – 7.31 (m, 2H), 7.29 – 7.19 (m, 6H), 6.96 (t, *J* = 7.3 Hz, 1H), 6.90 (d, *J* = 7.3 Hz, 1H), 6.51 (d, *J* = 1.9 Hz, 1H), 6.46 (dd, *J* = 8.4, 1.9 Hz, AB system, A part, 1H), 6.33 (d, *J* = 8.4 Hz, AB system, B part, 1H), 4.44 (s, 2H), 4.30 (t, *J* = 10.2 Hz, 1H), 4.24 – 4.16 (m, 1H), 3.71 (dd, *J* = 10.2, 7.6 Hz, 1H), 3.44 – 3.29 (m, 2H), 2.68 (t, *J* = 6.2 Hz, 2H), 2.36 (s, 3H), 2.06 – 1.94 (m, 2H). **^{13}C{^{1}H} NMR (101 MHz, CDCl$_3$) δ** 144.8, 144.1, 142.0, 138.9, 135.8, 133.9, 129.8, 129.7, 129.3, 128.7, 128.4, 128.0, 123.9, 122.5, 114.7, 111.0, 58.8, 55.3, 50.0, 45.5, 28.2, 22.4, 21.6. **HRMS (ESI-TOF)** *m/z*: [M + H]$^+$ calcd for $C_{31}H_{31}N_2O_2S$: 495.2101; found 495.2107.

8-Benzyl-3-phenyl-1-tosyl-2,3,5,6,7,8-hexahydro-1*H*-pyrrolo[3,2-*g*]quinoline (7). To a stirred solution of *N*-(2-(1-benzyl-7-bromo-1,2,3,4-tetrahydroquinolin-6-yl)-2-phenylethyl)-4-methylbenzenesulfonamide (**3ga**) (0.1 mmol, 58 mg), 1,2-diaminocyclohexane (2.3 mg, 20 mol%), and K_2CO_3 (21 mg, 0.15 mmol) in DMF (2 mL), CuI (2 mg, 10 mol %) was added at room temperature and then stirred at 120 °C for 4 h in an oil bath. After completion, the reaction mixture was cooled to room temperature and diluted with EtOAc (15 mL). The mixture was successively washed with brine (2 × 5 mL). Drying with Na_2SO_4 and evaporation of the solvent provided a residue that was purified by silica gel column chromatography using EtOAc/hexane (20:80) to give 1-benzyl-6-(1-tosylindolin-3-yl)-1,2,3,4-tetrahydroquinoline (**7**) as a white solid (42 mg, 86%; mp = 155–156 °C). **^{1}H NMR (400 MHz, CDCl$_3$) δ** 7.46 – 7.39 (m, 4H), 7.37 – 7.31 (m, 1H), 7.30 – 7.26 (m, AA'BB' system, AA' part, 2H), 7.22 – 7.16 (m, 3H), 7.10 – 7.03 (m, AA'BB' system, BB' part, 2H),

6.95 (s, 1H), 6.93 – 6.86 (m, 2H), 6.45 (s, 1H), 4.65 – 4.51 (m, 2H), 4.29 – 4.15 (m, 2H), 3.73 – 3.59 (m, 1H), 3.47 (t, *J* = 5.6 Hz, 2H), 2.65 (t, *J* = 6.2 Hz, 2H), 2.37 (s, 3H), 2.04 – 1.92 (m, 2H). **^{13}C{^{1}H} NMR (101 MHz, $CDCl_3$) δ** 145.2, 143.6, 141.3, 139.0, 133.6, 129.8, 129.6, 128.8, 128.6, 127.8, 127.6, 127.1, 127.0, 126.9, 125.6, 121.8, 118.2, 97.8, 59.0, 55.9, 50.8, 45.7, 28.0, 22.4, 21.7. **HRMS (ESI-TOF)** *m/z*: [M + H]$^+$ calcd for $C_{31}H_{31}N_2O_2S$: 495.2101; found 495.2108.

(±)-1,7,7-Trimethylbicyclo[2.2.1]heptan-2-yl-4-(1-(1-benzyl-1,2,3,4-tetrahydroquinolin-6-yl)-2-((4-methylphenyl)sulfonamido)ethyl)benzoate (3as). Synthesized according to General Procedure D at 80 °C, purified by silica gel chromatography with EtOAc/hexane (1:9). White solid (281 mg, 83%; mp = 101–102 °C). **^{1}H NMR (400 MHz, DMSO-*d*$_6$) δ** 7.89 – 7.78 (m, AA'BB' system, AA' part, 2H), 7.68 – 7.56 (m, 3H), 7.37 – 7.29 (m, 4H), 7.27 (d, *J* = 7.1 Hz, 2H), 7.24 – 7.13 (m, 3H), 6.81 – 6.65 (m, 2H), 6.31 (d, *J* = 8.3 Hz, AB system, B part, 1H), 5.00 (d, *J* = 9.5 Hz, 1H), 4.39 (s, 2H), 3.96 (t, *J* = 7.8 Hz, 1H), 3.39 – 3.32 (m, 2H), 3.31 – 3.26 (m, 2H), 3.22 – 3.12 (m, 1H), 2.64 (t, *J* = 6.0 Hz, 2H), 2.43 – 2.25 (m, 4H), 2.10 – 1.98 (m, 1H), 1.91 – 1.80 (m, 2H), 1.78 – 1.65 (m, 2H), 1.35 (t, *J* = 12.6 Hz, 1H), 1.30 – 1.19 (m, 1H), 1.02 (dd, *J* = 13.6, 3.0 Hz, 1H), 0.90 (s, 3H), 0.86 (s, 3H), 0.83 (s, 3H). **^{13}C{^{1}H} NMR (101 MHz, DMSO-*d*$_6$) δ** 165.7, 148.7, 143.8, 142.5, 138.9, 137.4, 129.5, 129.1, 128.4, 128.2, 128.2, 128.1, 128.0, 126.6, 126.53, 126.49, 126.0, 121.7, 110.6, 79.5, 54.2, 49.8, 49.5, 48.7, 47.5, 46.8, 46.7, 44.3, 36.4, 27.6, 26.9, 21.8, 20.9, 19.5, 18.6, 13.4. **HRMS (ESI-TOF)** *m/z*: [M + H]$^+$ calcd for $C_{42}H_{49}N_2O_4S$: 677.3408; found 677.3419.

(±)-2-Isopropyl-5-methylcyclohexyl 4-(1-(1-benzyl-1,2,3,4-tetrahydroquinolin-6-yl)-2-((4-methylphenyl)sulfonamido)ethyl)benzoate (3at). Synthesized according to General Procedure D at 80 °C, purified by silica gel chromatography with EtOAc/hexane (1:9). White solid (295 mg, 87%; mp = 92–93 °C). **^{1}H NMR (400 MHz, DMSO-*d*$_6$) δ** 7.84 – 7.77 (m, AA'BB' system, AA' part, 2H), 7.66 – 7.58 (m, 3H), 7.34 – 7.25 (m, 5H), 7.22 – 7.16 (m, 3H), 6.76 – 6.68 (m, 2H), 6.31 (d, *J* = 8.1 Hz, AB system, B part, 1H), 4.81 (td, *J* = 10.9, 4.4 Hz, 1H), 4.39 (s, 2H), 3.95 (t, *J* = 8.0 Hz, 1H), 3.35 – 3.32 (m, 2H), 3.31 – 3.26 (m, 2H), 3.22 – 3.12 (m, 1H), 2.64 (t, *J* = 5.6 Hz, 2H), 2.34 (s, 3H), 1.95 (d, *J* = 12.0 Hz, 1H), 1.88 – 1.79 (m, 3H), 1.65 (d, *J* = 11.5 Hz, 2H), 1.50 (t, *J* = 11.5 Hz, 2H), 1.13 – 0.99 (m, 2H), 0.92 – 0.80 (m, 7H), 0.72 (d, *J* = 6.9 Hz, 3H). **^{13}C{^{1}H} NMR (101 MHz, DMSO-*d*$_6$) δ** 165.01, 148.67, 148.64, 143.80, 142.44, 138.93, 137.42, 129.51, 129.13, 128.43, 128.21, 128.14, 128.08, 127.87, 126.56, 126.49 (2C), 125.98, 121.71, 110.61, 73.86, 54.19, 49.79, 49.45, 46.75, 46.71, 46.62, 40.52, 33.72, 30.85, 27.59, 26.20, 23.23, 21.87, 21.76, 20.93, 20.42, 16.45. **HRMS (ESI-TOF)** *m/z*: [M + H]$^+$ calcd for $C_{42}H_{51}N_2O_4S$: 679.3564; found 679.3569.

4. Gram-scale synthesis of 3aa and recovery of HFIP

The reaction was carried out according to General Procedure D using 1.0 g (3.7 mmol) of 2-phenyl-1-tosylaziridine (**2a**) and 30 mL of HFIP. Upon completion of the reaction, HFIP was recovered by simple distillation, affording 25 mL (83%) of the solvent. After purification, compound **3aa** was obtained in 1.41 g (78%).

5. Mechanistic experiments

Free-radical inhibition experiments: The experiments were performed according to general procedure D with *N*-benzyl tetrahydroquinoline (**1a**; 224 mg, 1.0 mmol, 2.0 equiv) and phenyl aziridine (**2a**; 137 mg, 0.5 mmol, 1.0 equiv) and TEMPO (156 mg, 1 mmol) or BHT (224 mg, 1 mmol). After purification, **3aa** was obtained with 55% and 80% yields, respectively.

NMR Analysis of 2a in the Absence and Presence of HFIP

To evaluate the interaction between 2-phenyl-1-tosylaziridine (**2a**) and HFIP, comparative ^{1}H and ^{13}C NMR experiments were conducted. The compound was dissolved in $CDCl_3$ at a concentration of approximately 10 mg/0.6 mL, and spectra were recorded at 25 °C on a 400 MHz NMR spectrometer, both in the absence and in the presence of 10 equivalents of HFIP (Figures S3 and S4).

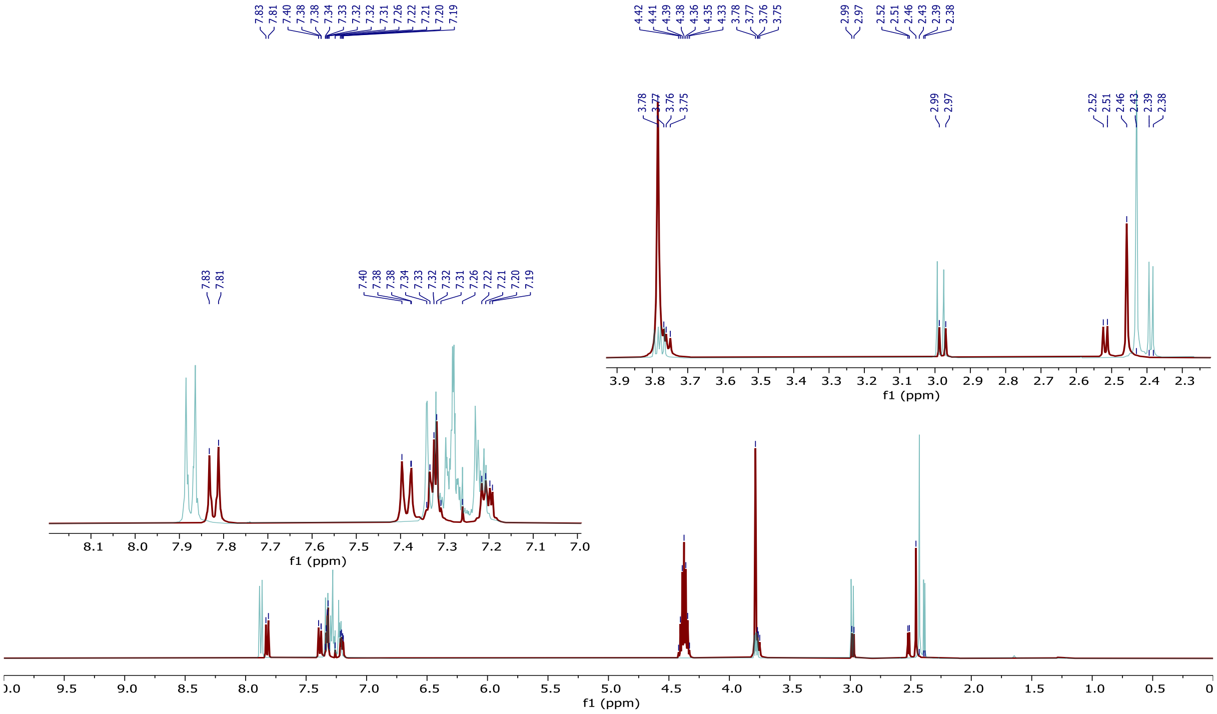

Figure S3. ^{1}H NMR study of compound **2a** ($CDCl_3$, 400 MHz) with HFIP (red colour spectrum) and without HFIP (blue colour spectrum).

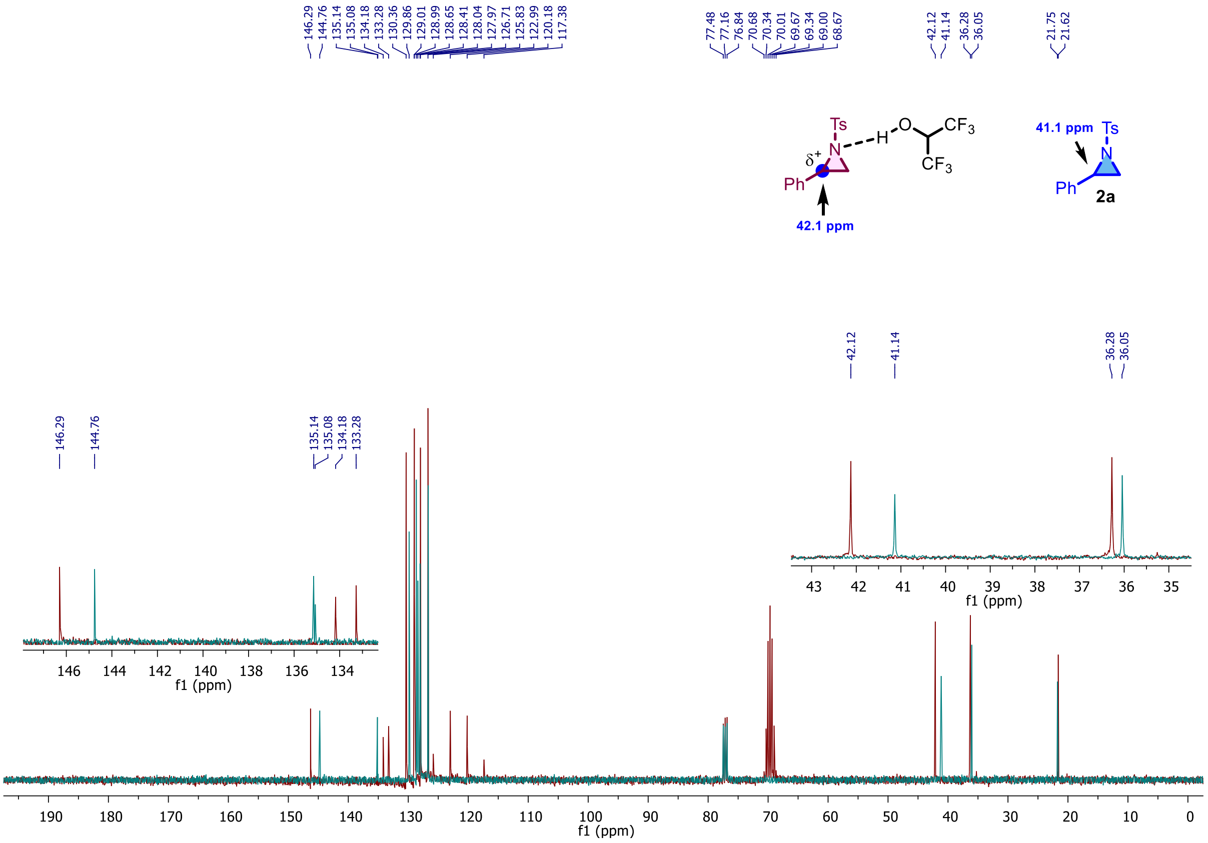

Figure S4. ^{13}C NMR study of compound **2a** ($CDCl_3$, 100 MHz) with HFIP (red colour spectrum) and without HFIP (blue colour spectrum).

6. References

(1) Zhang, W.; Xu, G.; Qiu, L.; Sun, J. Gold-Catalyzed C5-Alkylation of Indolines and Sequential Oxidative Aromatization: Access to C5-Functionalized Indoles. *Org. Biomol. Chem.* **2018**, *16*, 3889–3892.

(2) Taskesenligil, Y.; Aslan, M.; Ardahanli, R.; Saracoglu, N. Synthesis of Indoline- and 1,2,3,4-Tetrahydroquinoline-based Symmetrical Triarylmethanes. *J. Org. Chem.* **2025**, *90*, 28, 9954–9964.

(3) Dağalan, Z.; Can, H.; Daştan, A.; Nişancı, B.; Metin, Ö. Highly efficient hydrogenation and dehydrogenation of N-Heteroarenes catalyzed by mesoporous graphitic carbon nitride supported CoPd alloy nanoparticles. *Tetrahedron*. **2022,** *114*, 132766.

(4) Yi, N.; Liu, Y.; Xiong, Y.; Gong, H.; Tan, J.-P.; Fang, Z.; Yi, B. Gold-Catalyzed Intramolecular Hydroarylation and Transfer Hydrogenation of N-Aryl Propargylamines to Construct Tetrahydroquinolines and 5,6-Dihydro-4H-pyrrolo[3,2,1-ij]quinolines. *J. Org. Chem.* **2023**, *88*, 11945,

(5) Karjee, P.; Sarkar, T.; Kar, S.; Punniyamurthy, T. Transition-Metal-Free Stereospecific Oxidative Annulative Coupling of Indolines with Aziridines. *J. Org. Chem.* **2020**, *85*, 8261–8270.

(6) Zhang, L.; Wang, H.; Santiago, T. G.; Yue, W. J.; Martin, R. Photoinduced Nickel-Catalysed Enantioconvergent sp^3–sp^3 Coupling of Unactivated Olefins and Aziridines. *Nat. Catal.* **2025**, *8*, 1–9.

(7) Kok, G. P. Y.; Yang, H.; Wong, M. W.; Zhao, Y. Cu-Catalyzed [3+3] Cycloaddition of Isocyanoacetates with Aziridines and Stereoselective Access to α,β-Diamino Acids. *Org. Lett.* **2018**, *20*, 5112–5115.

7. NMR Spectra of Compounds

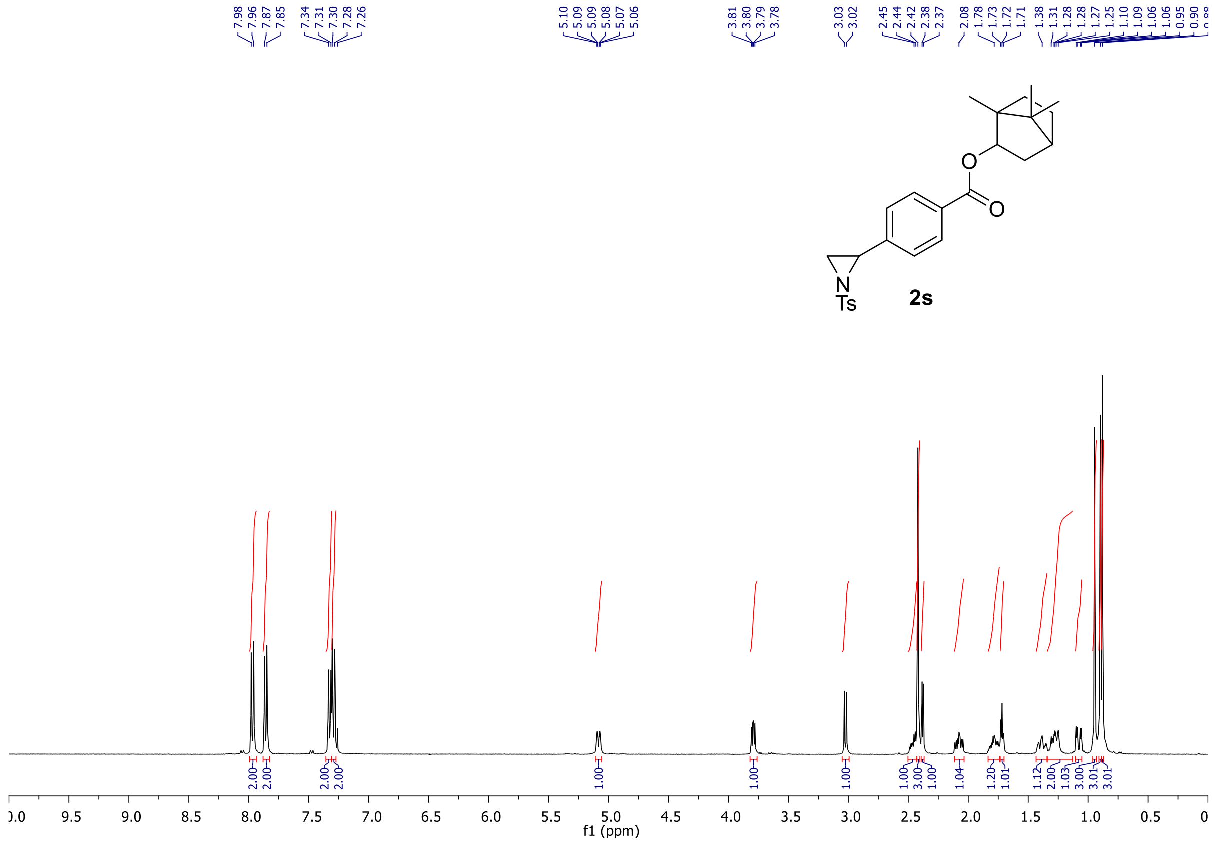

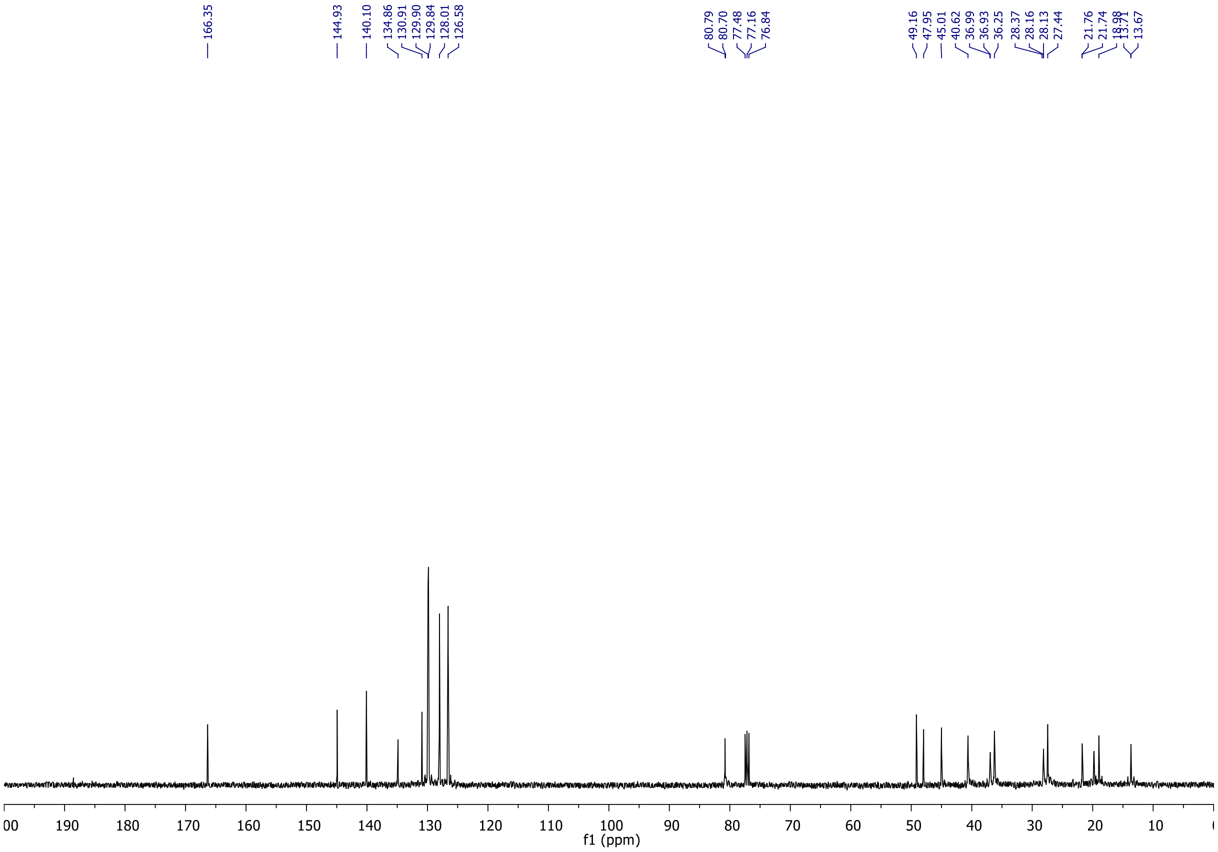

^{1}H NMR (400 MHz) and ^{13}C{^{1}H} NMR (100 MHz) spectra of **2s** ($CDCl_3$)

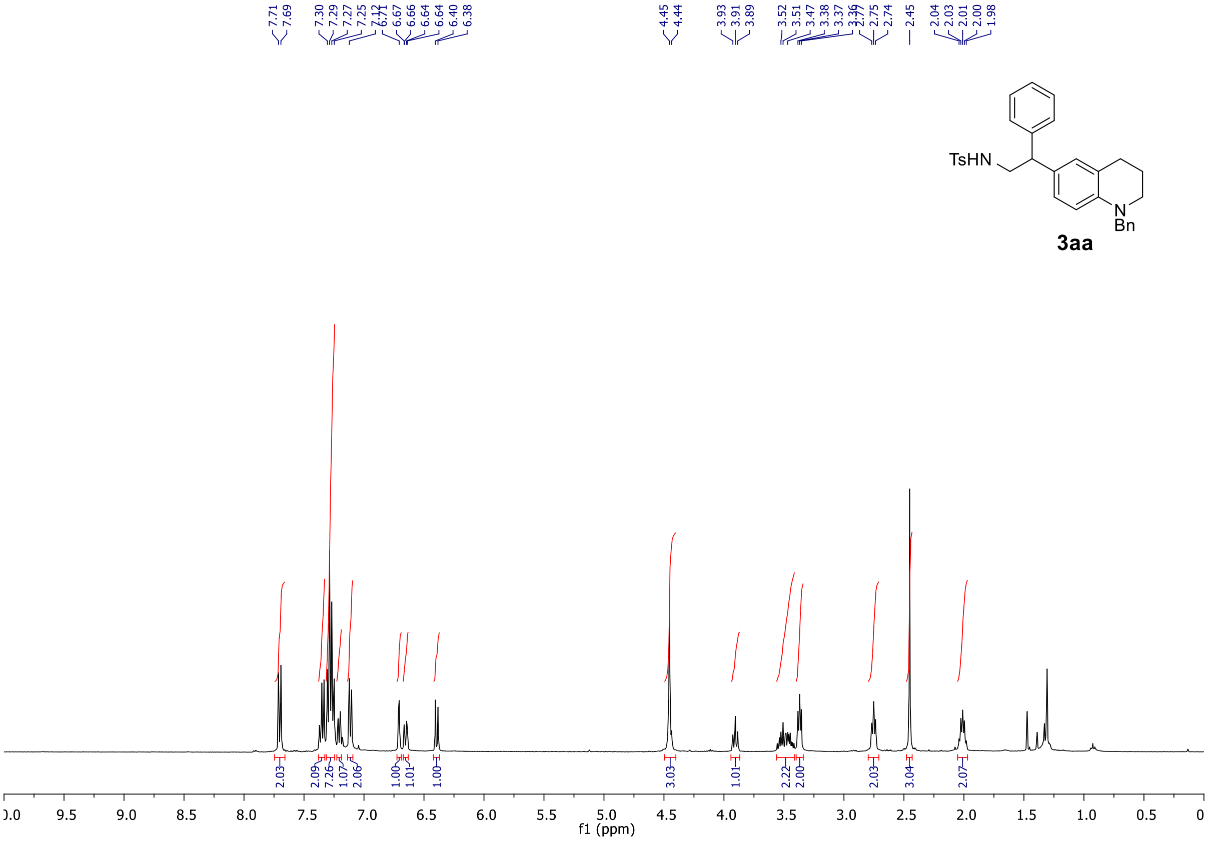

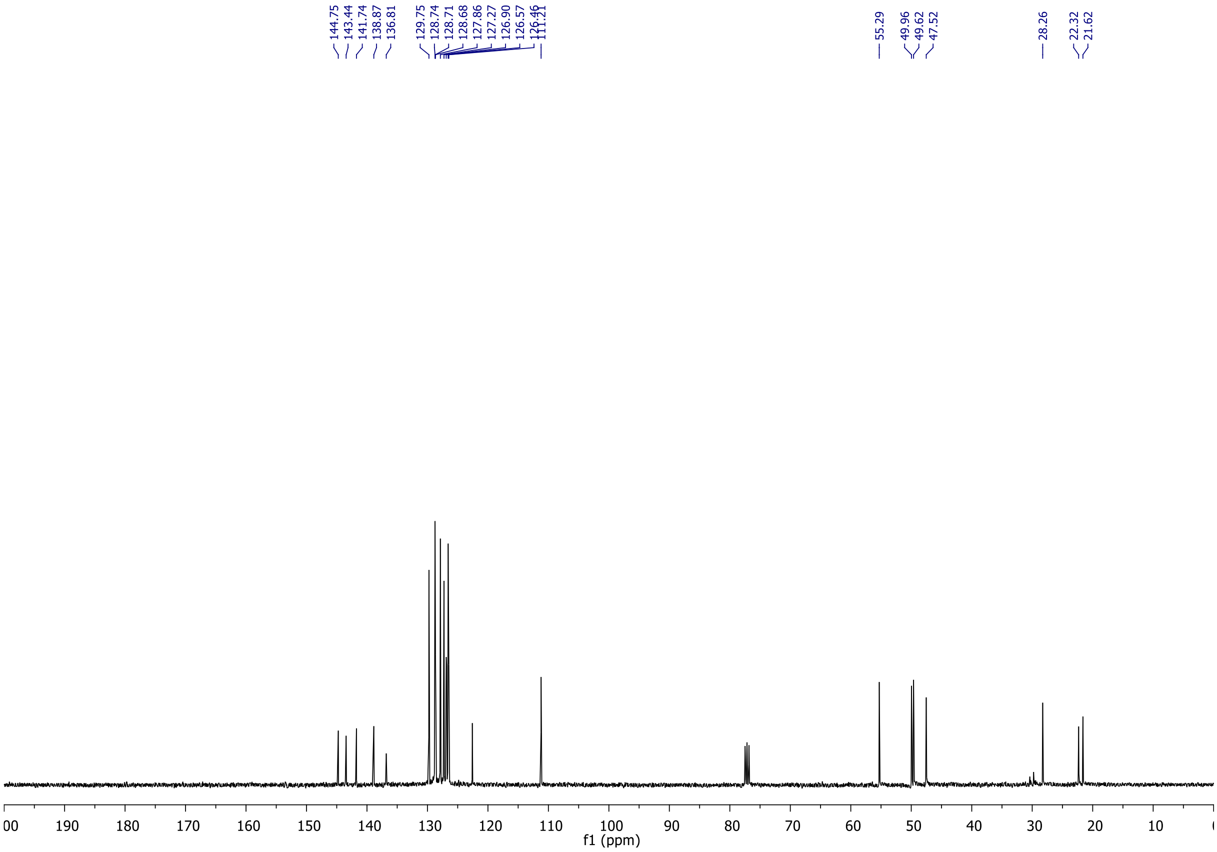

^{1}H NMR (400 MHz) and ^{13}C{^{1}H} NMR (100 MHz) spectra of **3aa** ($CDCl_3$)

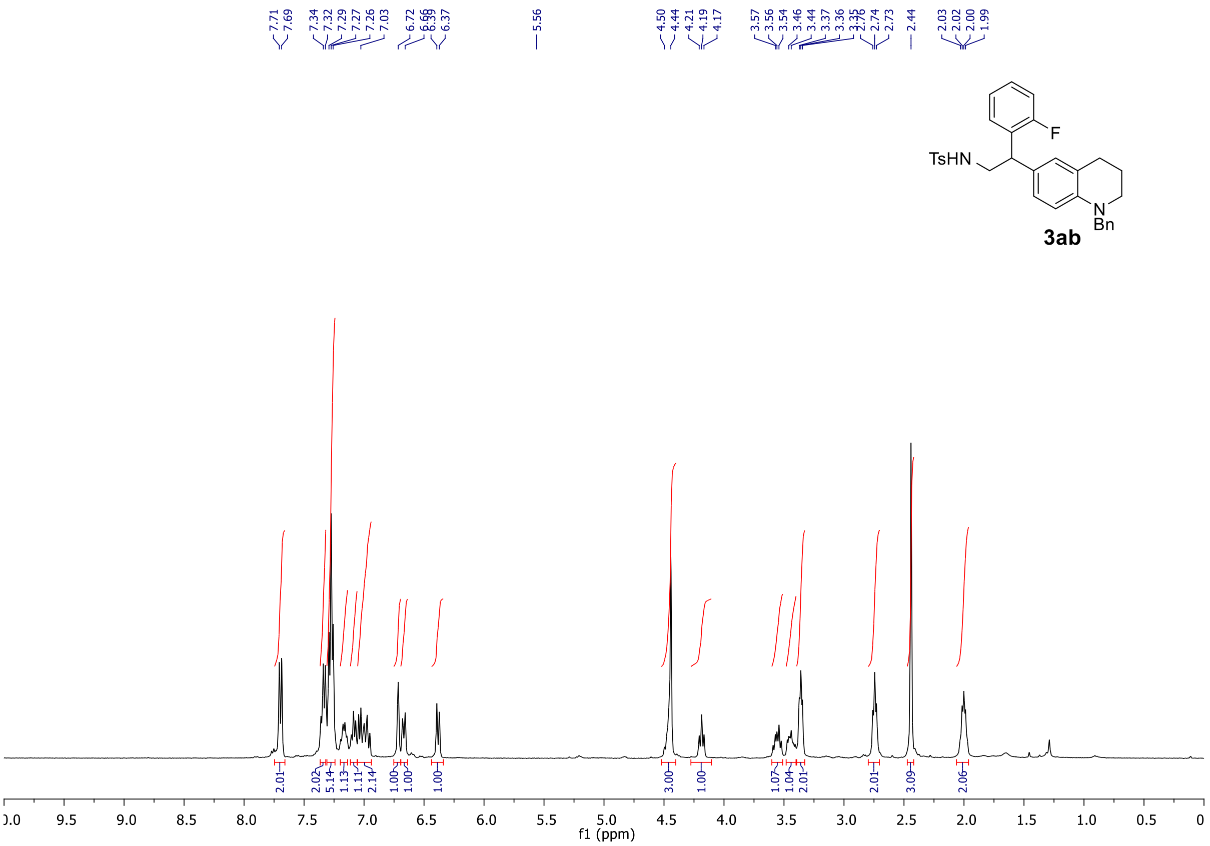

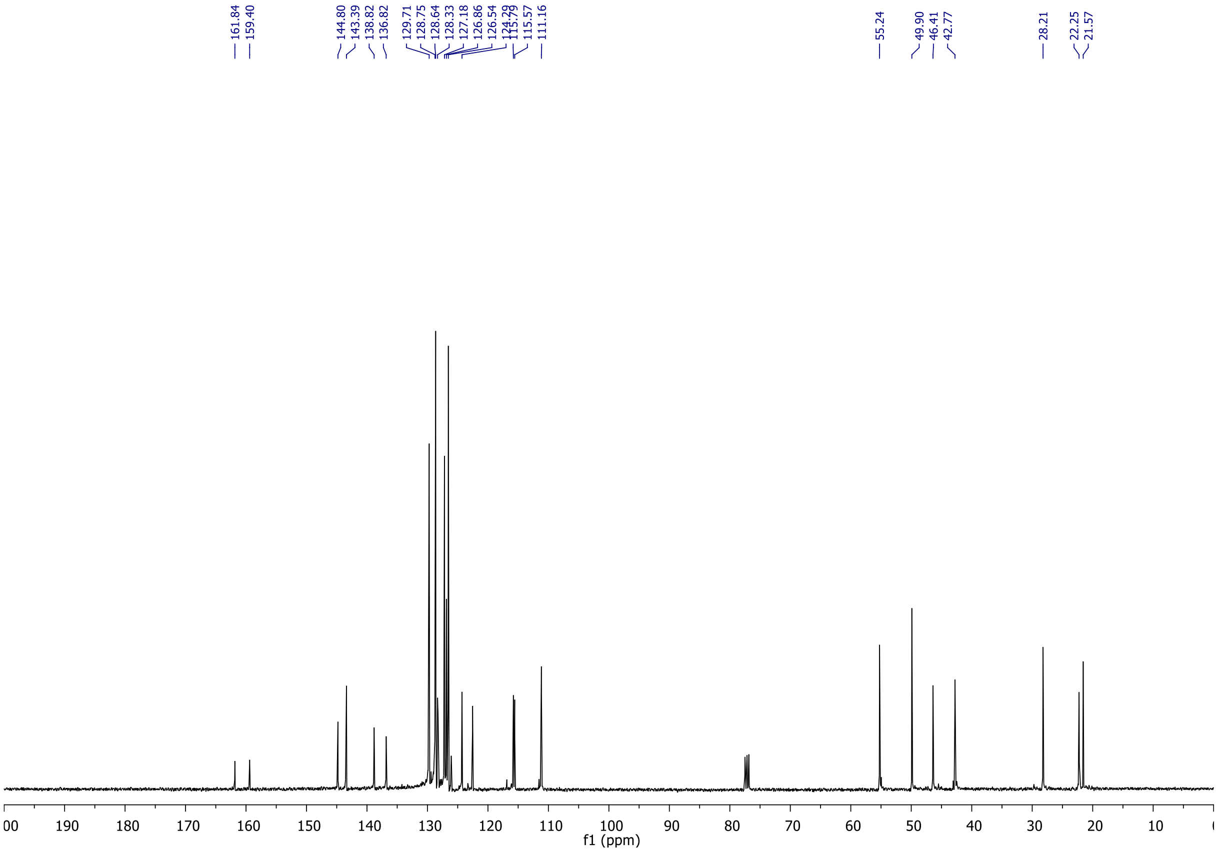

^{1}H NMR (400 MHz) and ^{13}C{^{1}H} NMR (100 MHz) spectra of **3ab** ($CDCl_3$)

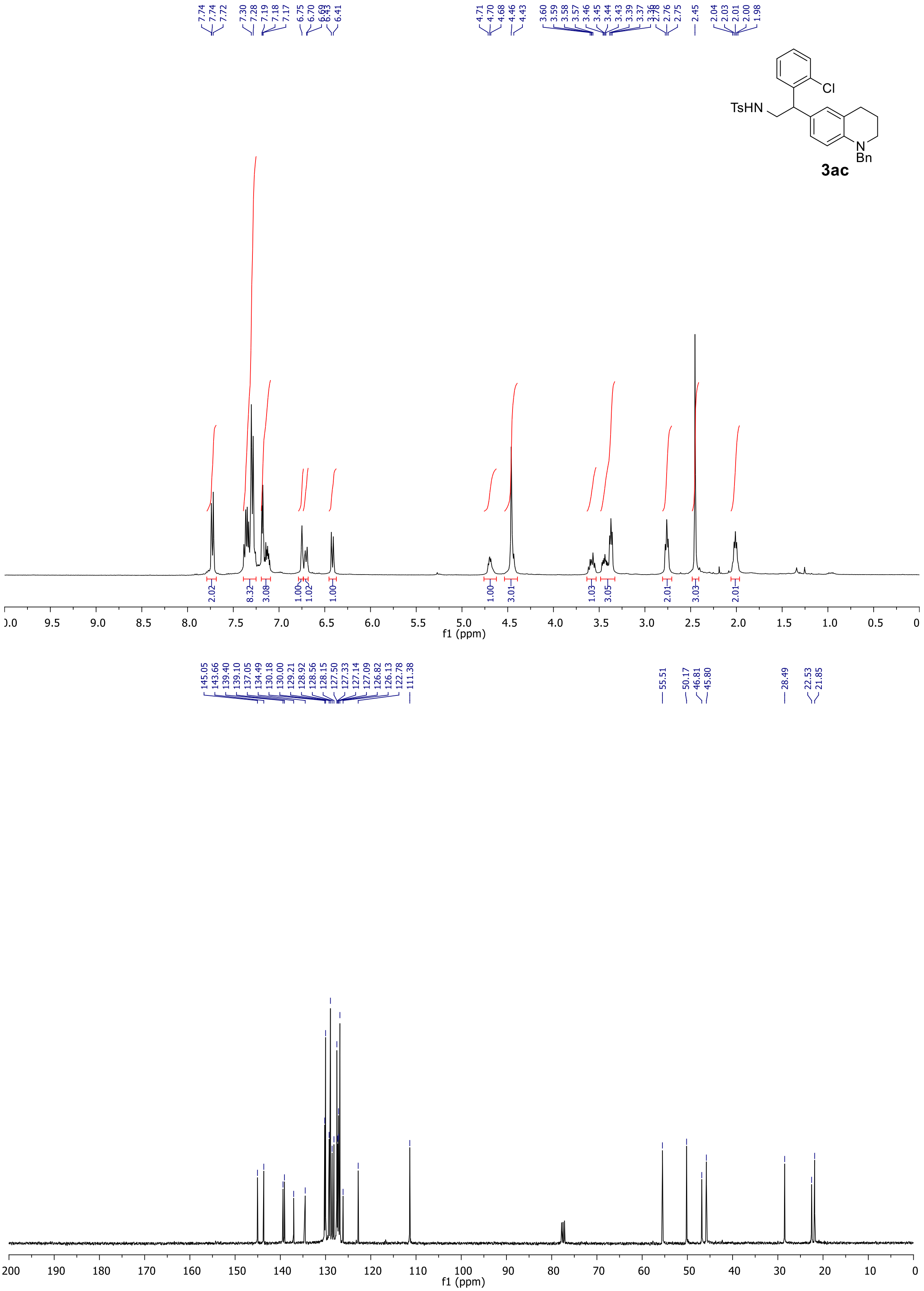

^{1}H NMR (400 MHz) and ^{13}C{^{1}H} NMR (100 MHz) spectra of **3ac** ($CDCl_3$)

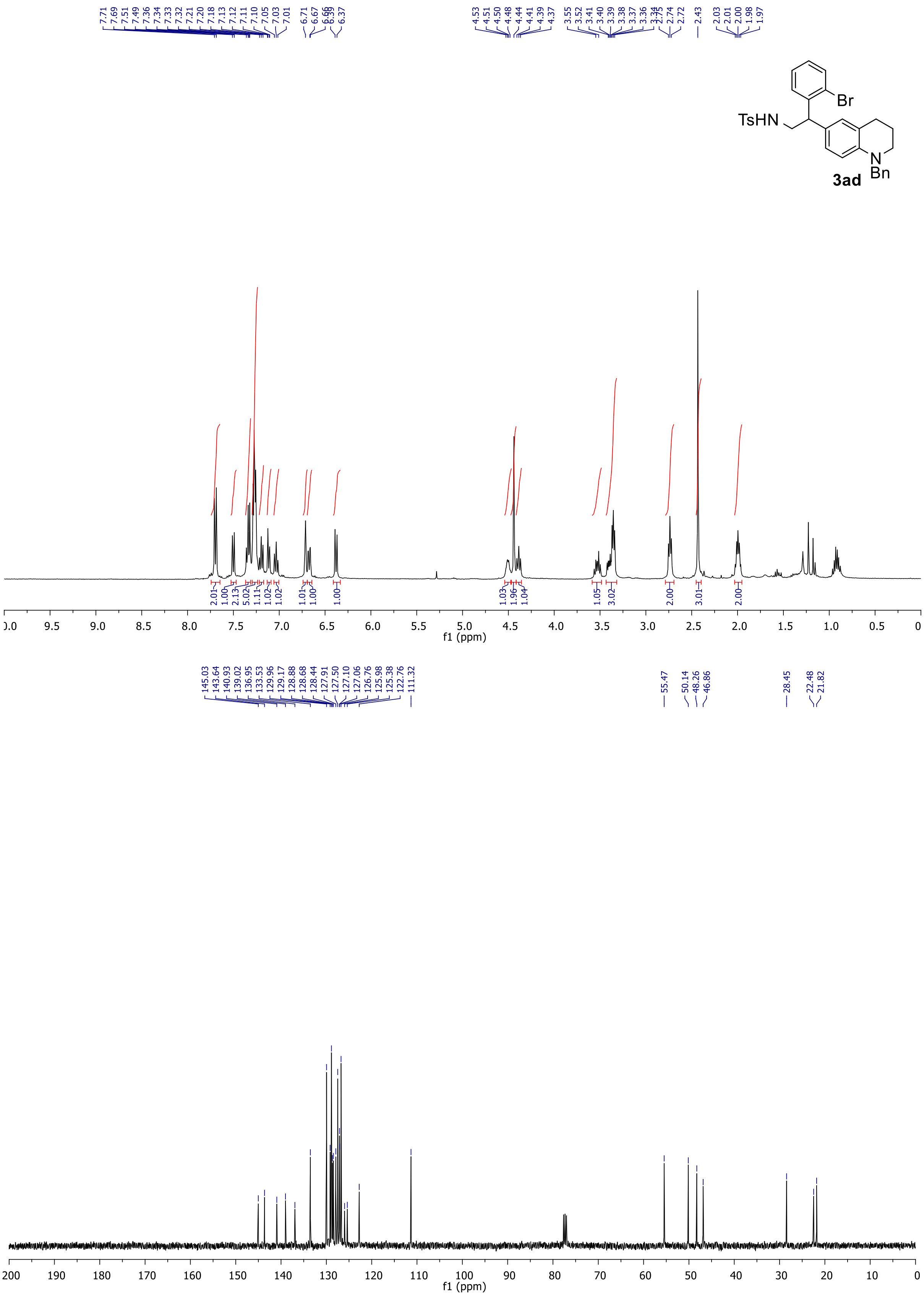

^{1}H NMR (400 MHz) and $^{13}C\{^{1}H\}$ NMR (100 MHz) spectra of **3ad** ($CDCl_3$)

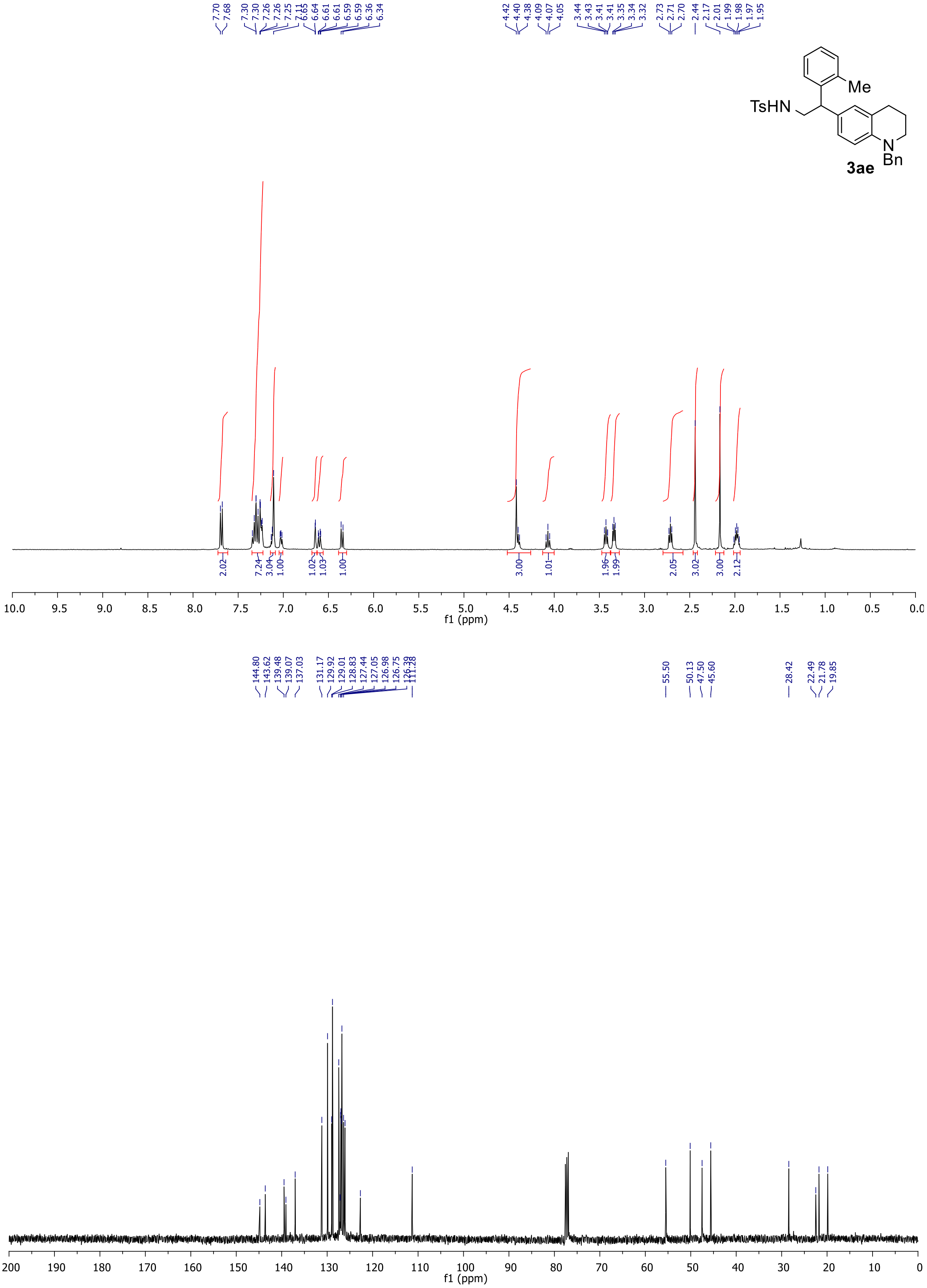

^{1}H NMR (400 MHz) and $^{13}C\{^{1}H\}$ NMR (100 MHz) spectra of **3ae** ($CDCl_3$)

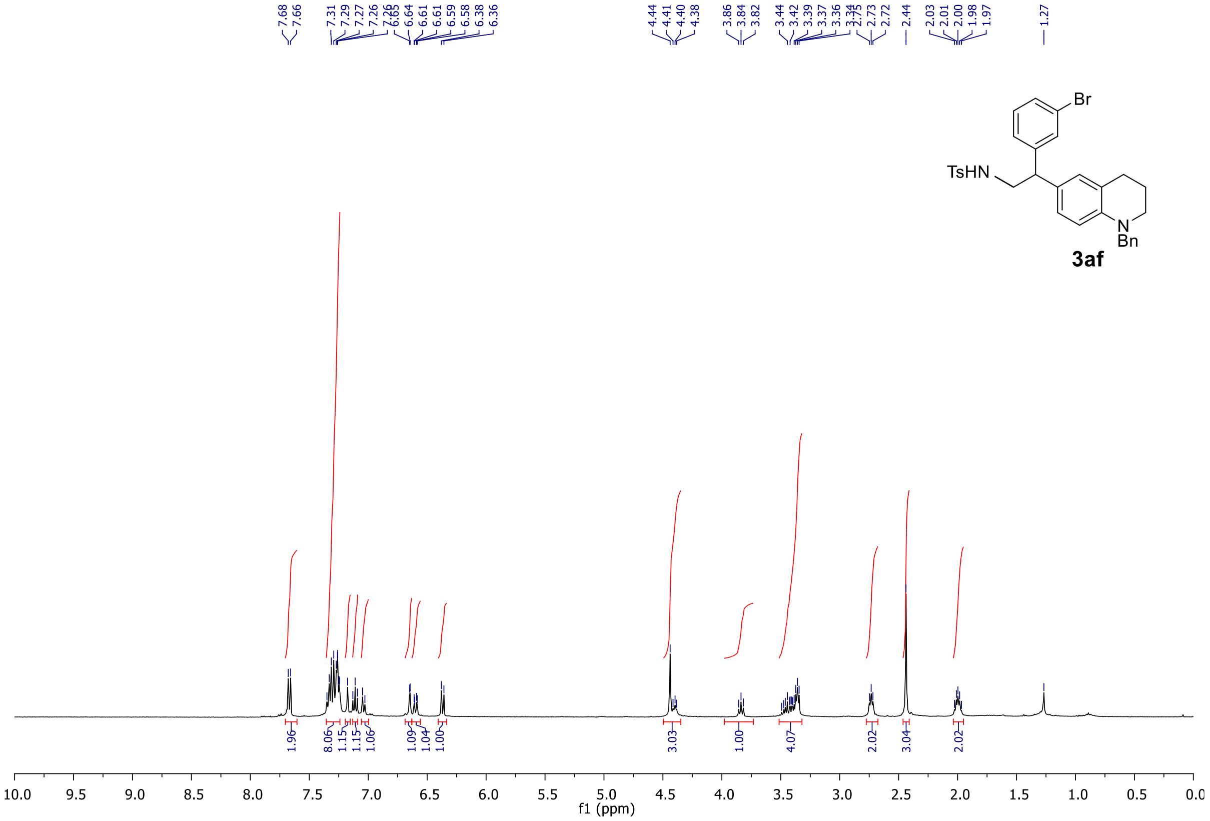

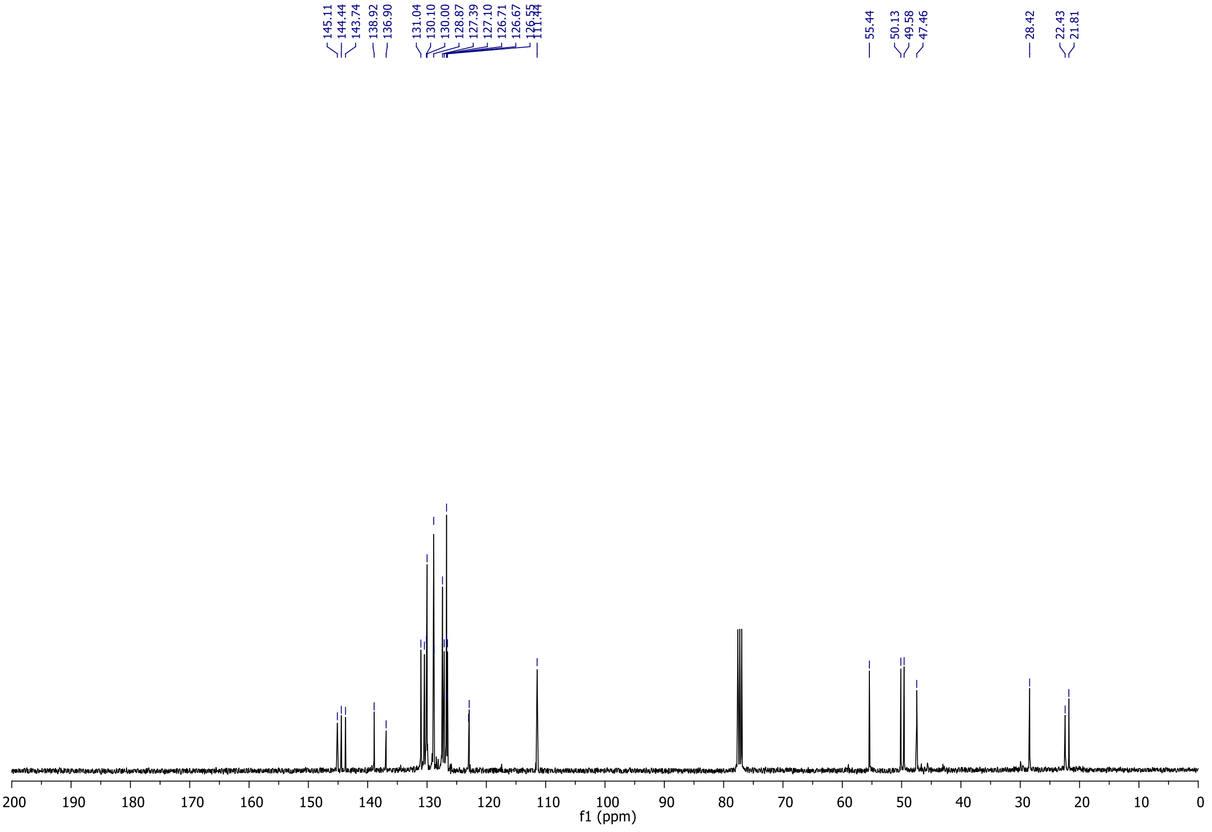

^{1}H NMR (400 MHz) and ^{13}C{^{1}H} NMR (100 MHz) spectra of **3af** ($CDCl_3$)

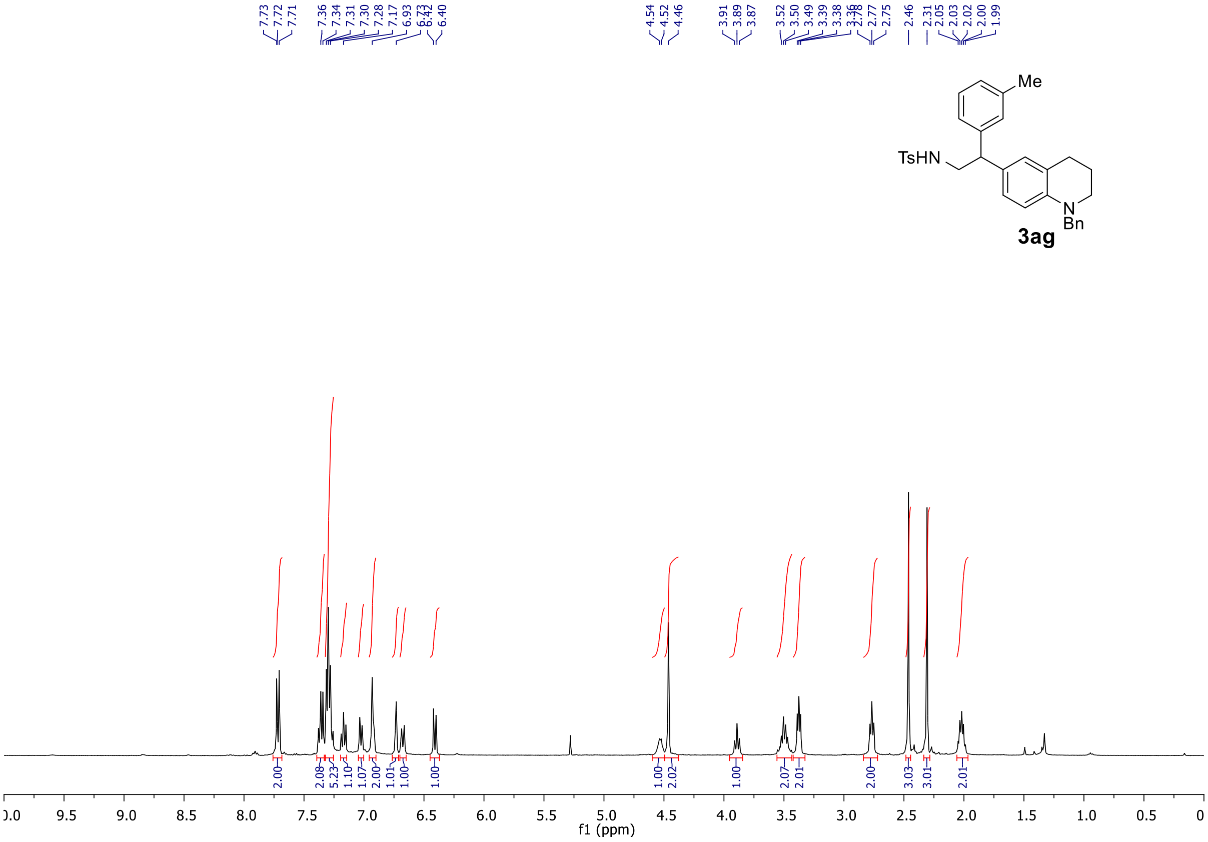

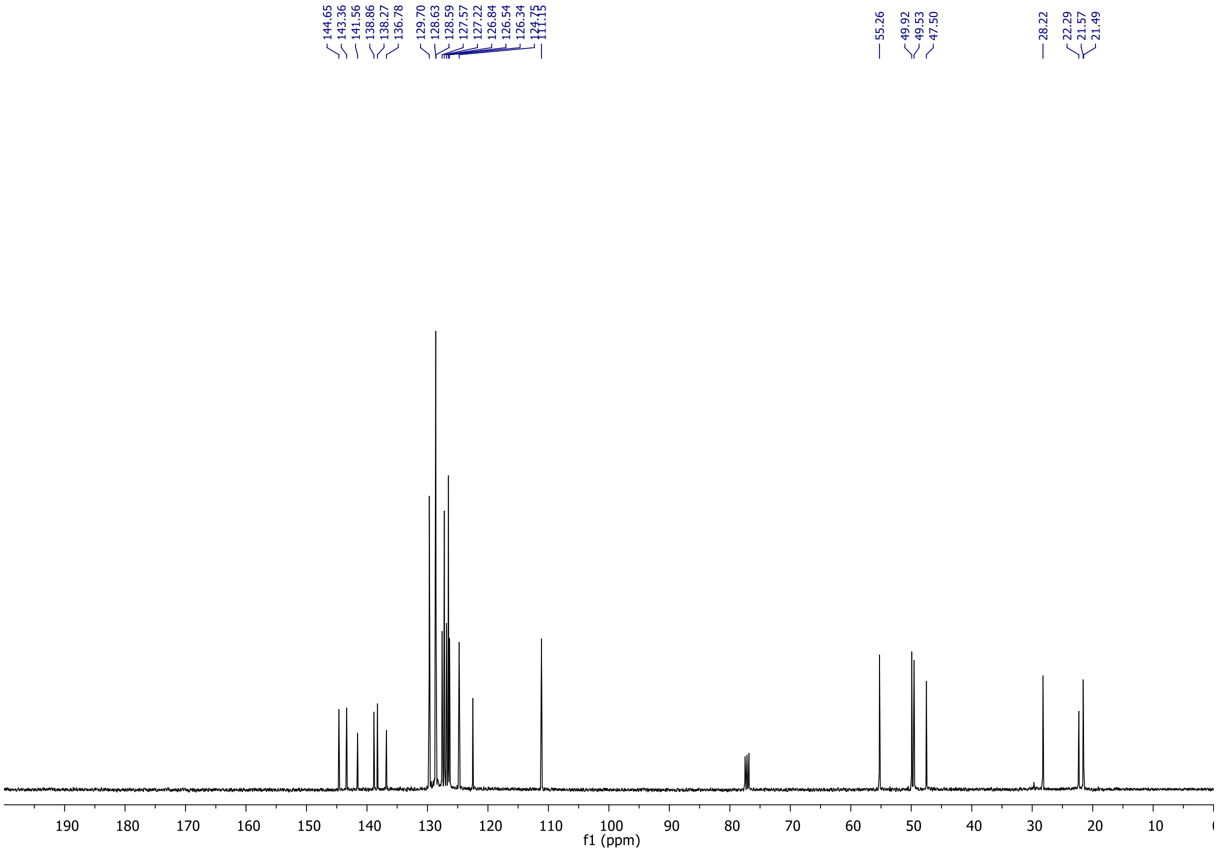

^{1}H NMR (400 MHz) and ^{13}C{^{1}H} NMR (100 MHz) spectra of **3ag** ($CDCl_3$)

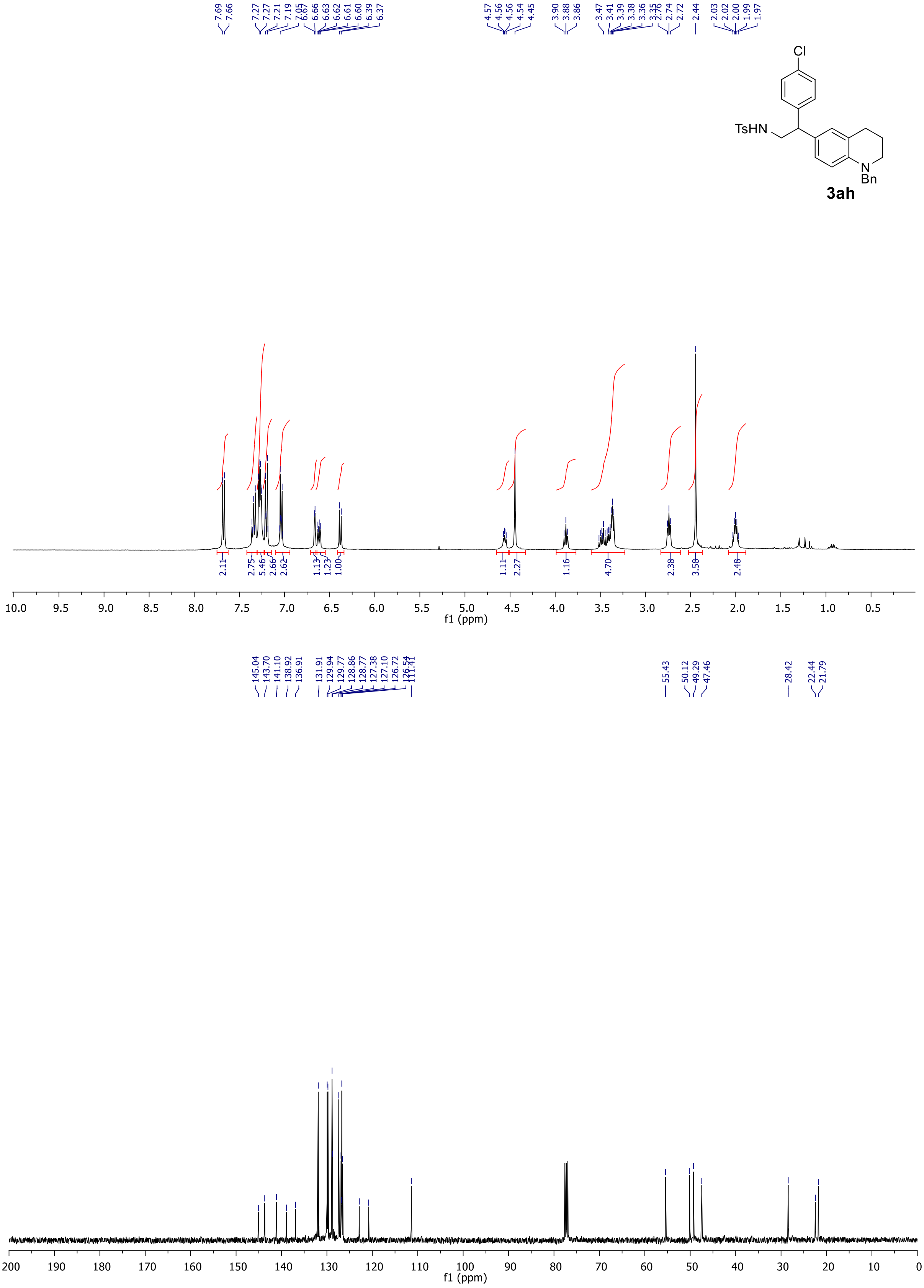

^{1}H NMR (400 MHz) and ^{13}C{^{1}H} NMR (100 MHz) spectra of **3ah** ($CDCl_3$)

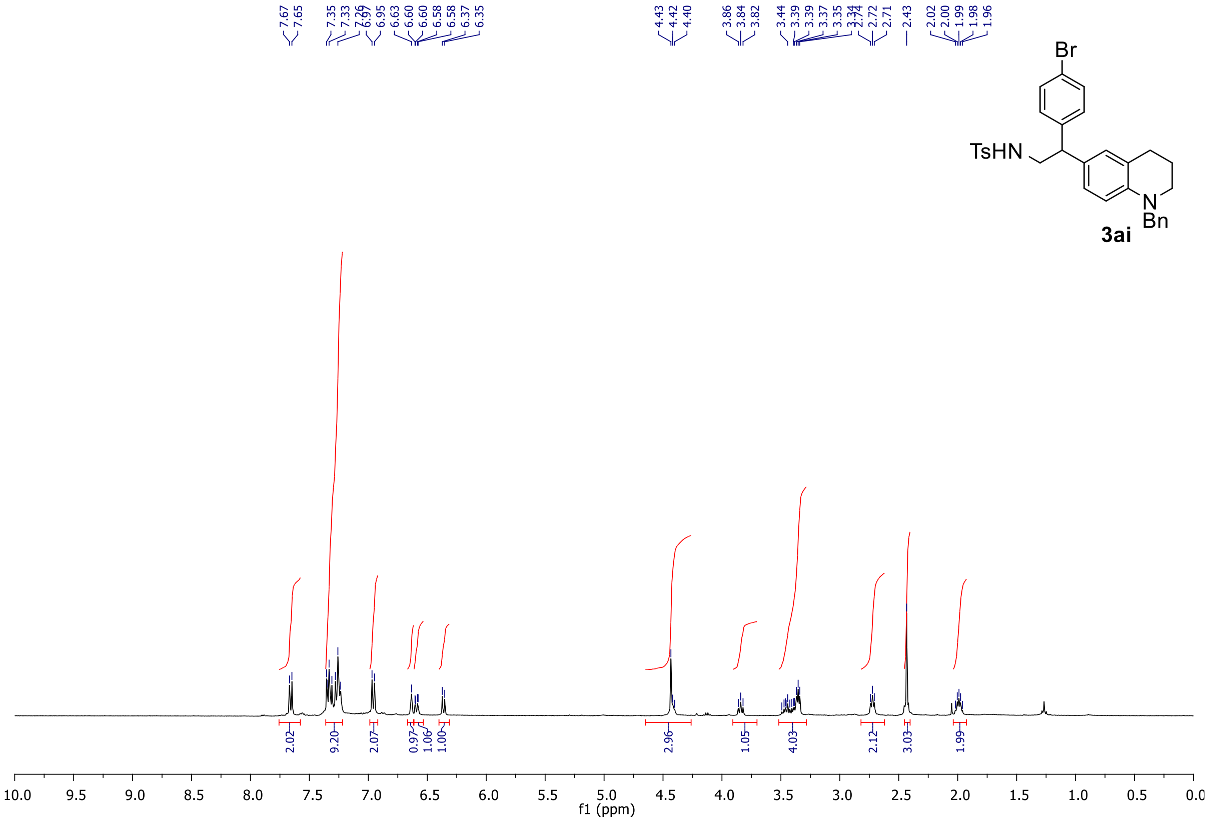

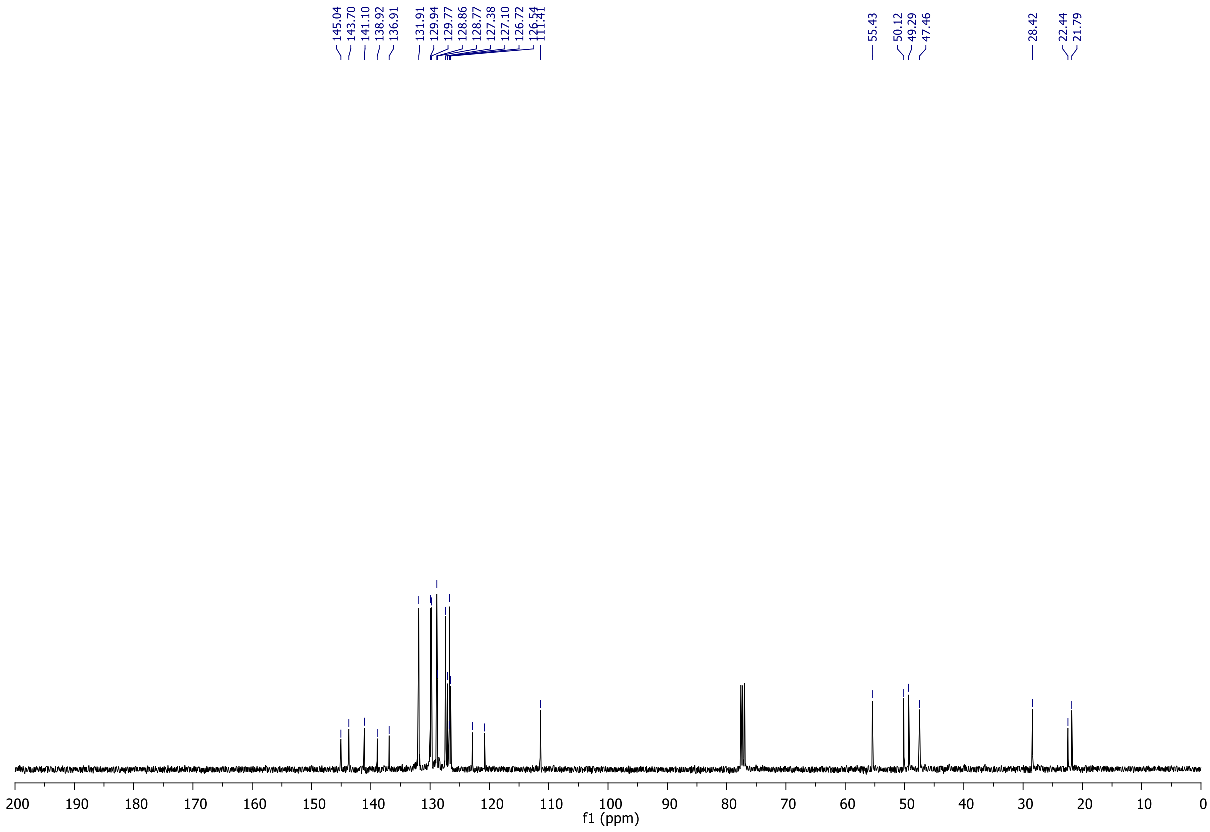

^{1}H NMR (400 MHz) and ^{13}C{^{1}H} NMR (100 MHz) spectra of **3ai** ($CDCl_3$)

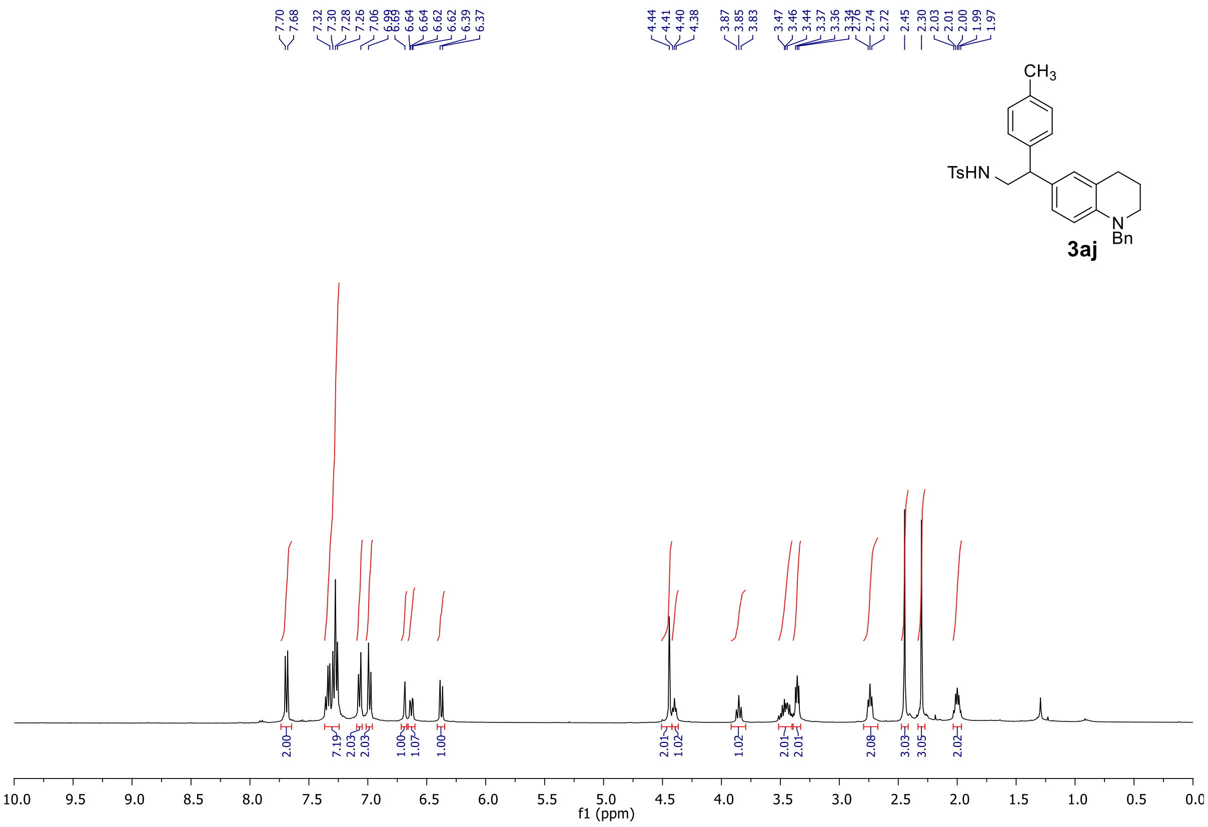

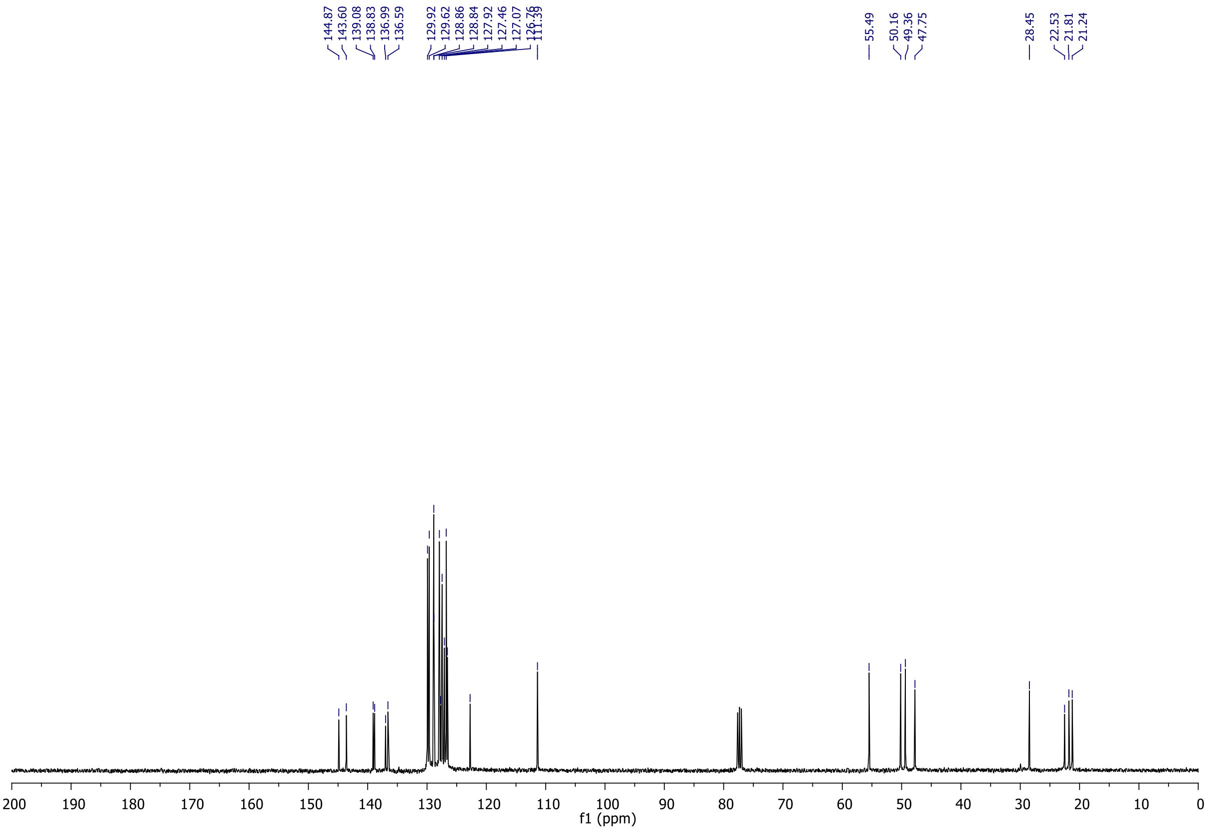

^{1}H NMR (400 MHz) and $^{13}C\{^{1}H\}$ NMR (100 MHz) spectra of **3aj** ($CDCl_3$)

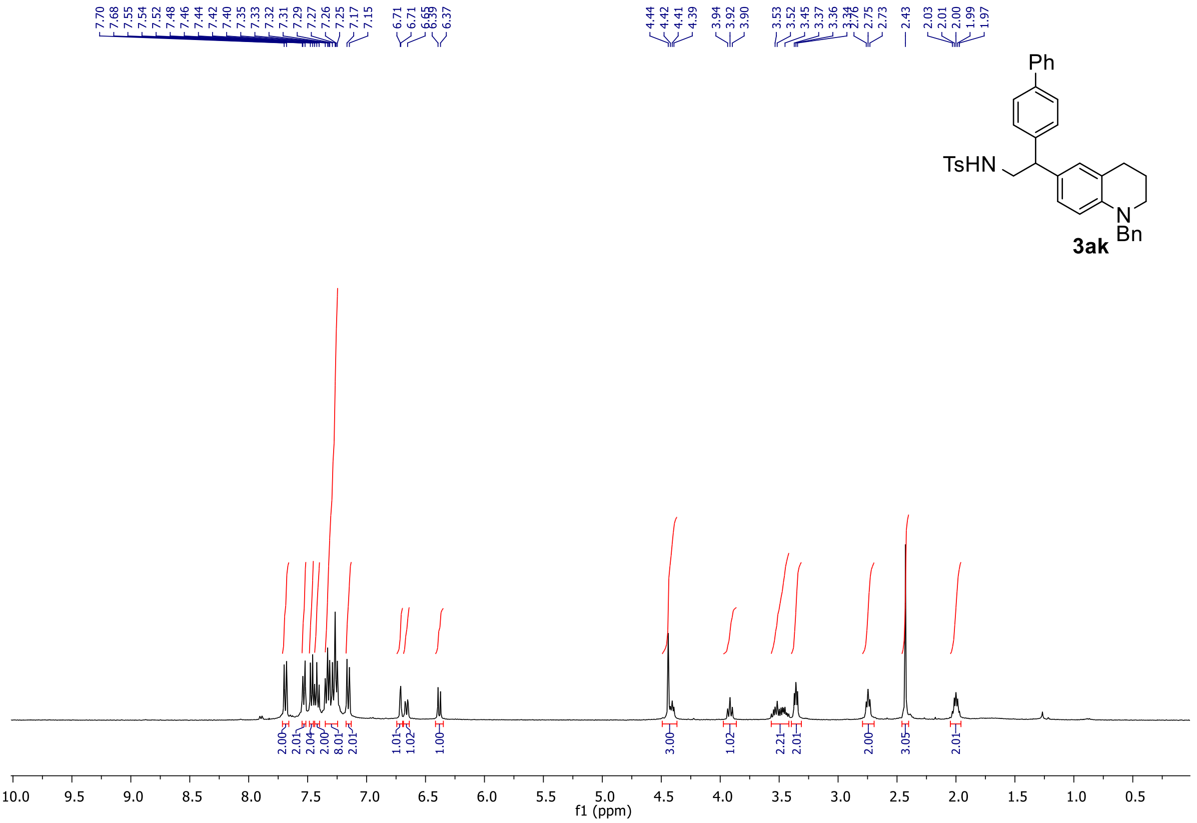

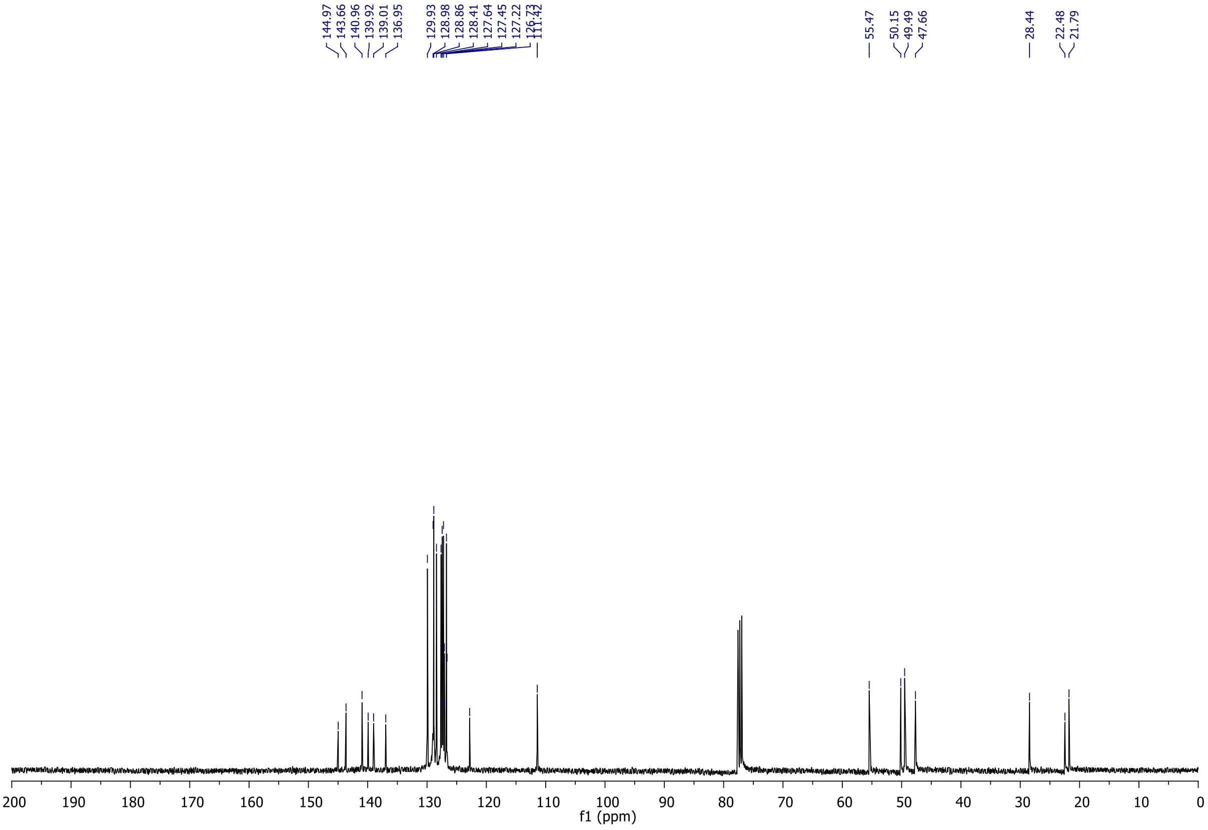

^{1}H NMR (400 MHz) and ^{13}C{^{1}H} NMR (100 MHz) spectra of **3ak** ($CDCl_3$)

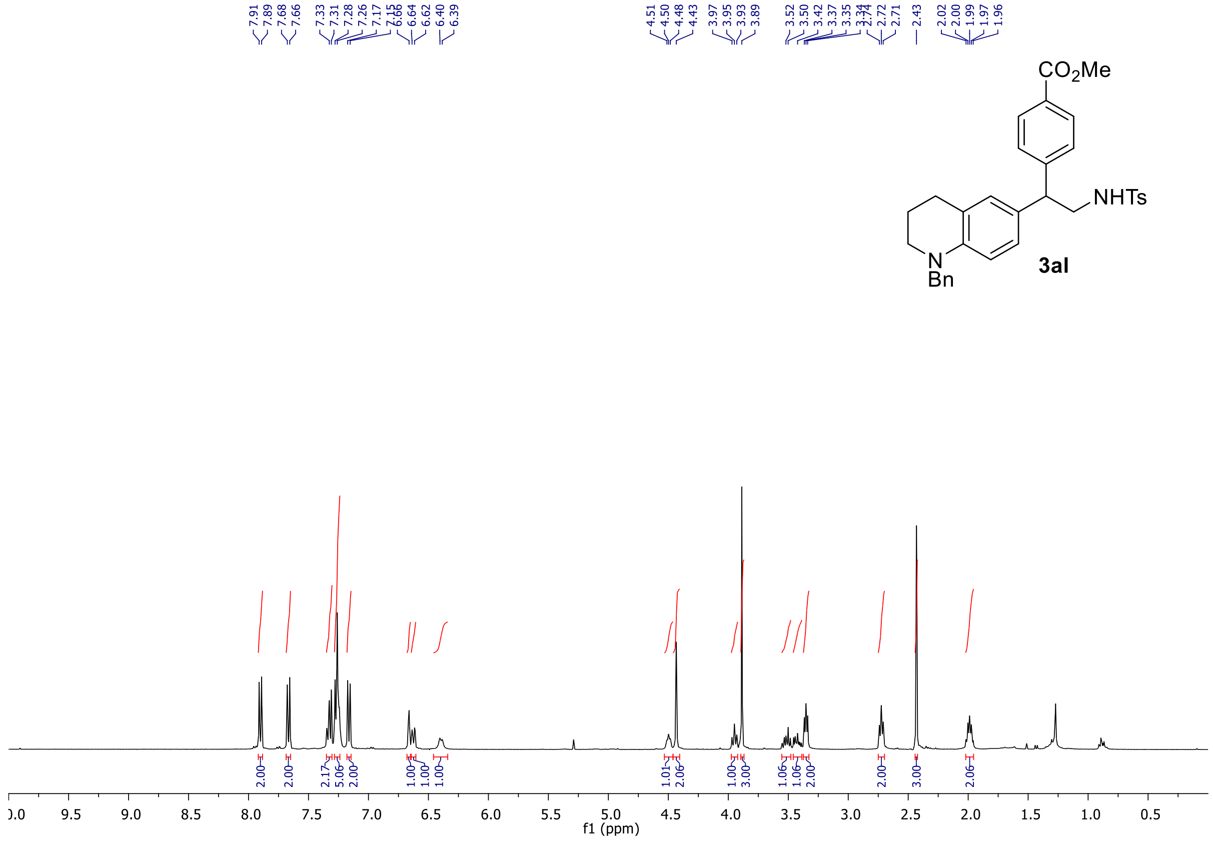

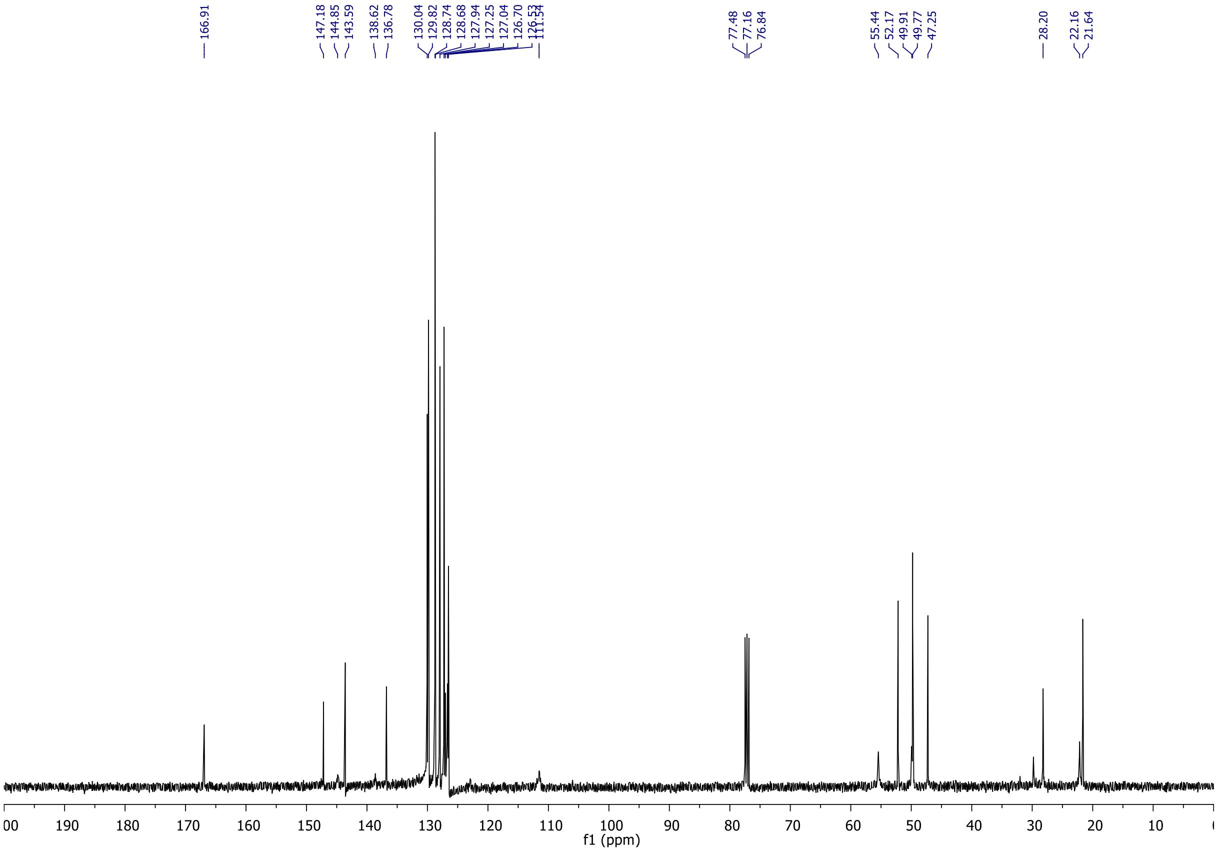

^{1}H NMR (400 MHz) and ^{13}C{^{1}H} NMR (100 MHz) spectra of **3al** ($CDCl_3$)

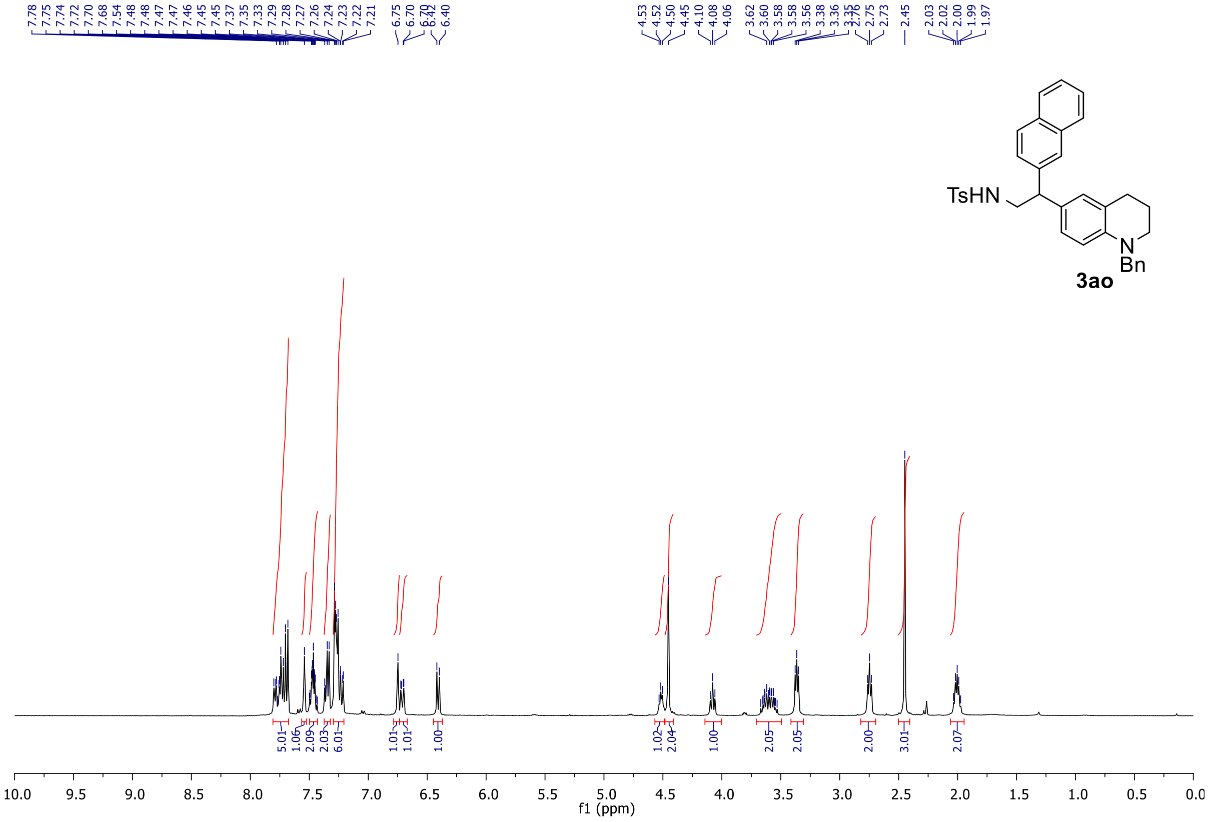

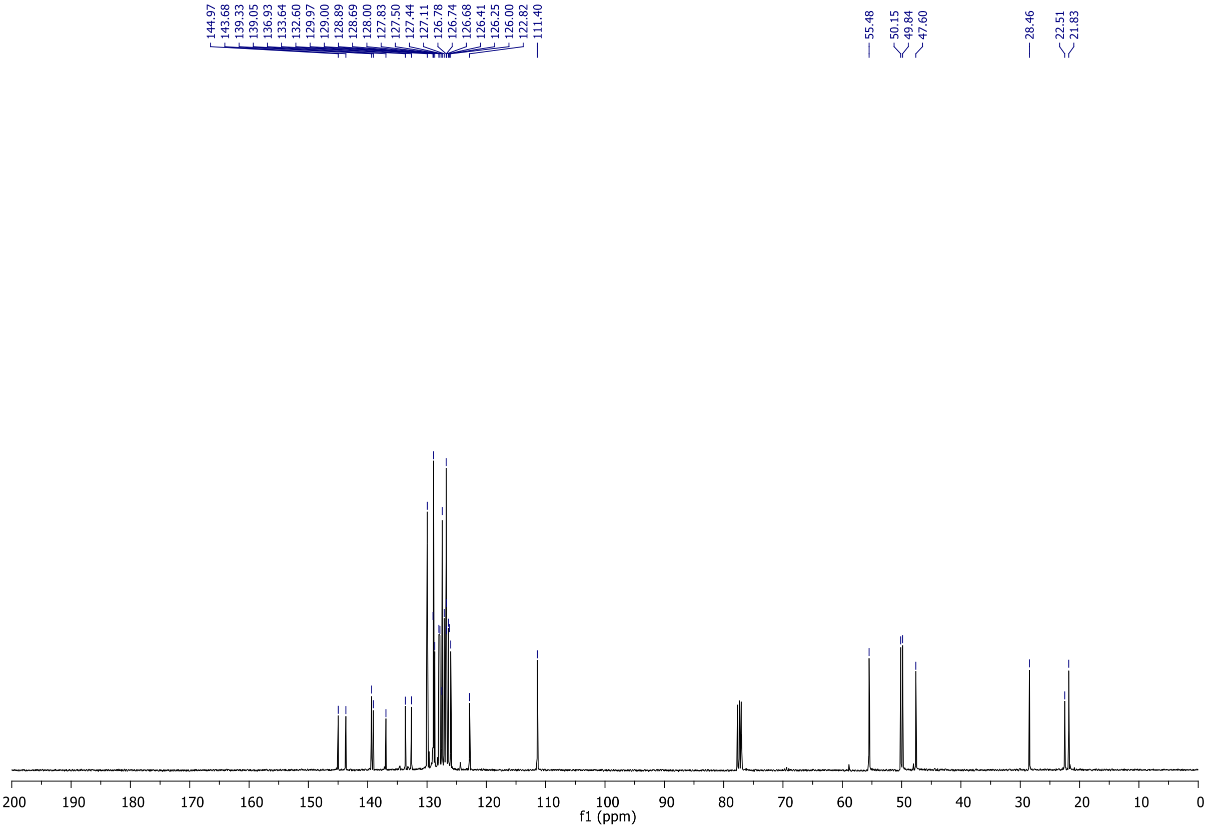

^{1}H NMR (400 MHz) and ^{13}C{^{1}H} NMR (100 MHz) spectra of **3ao** ($CDCl_3$)

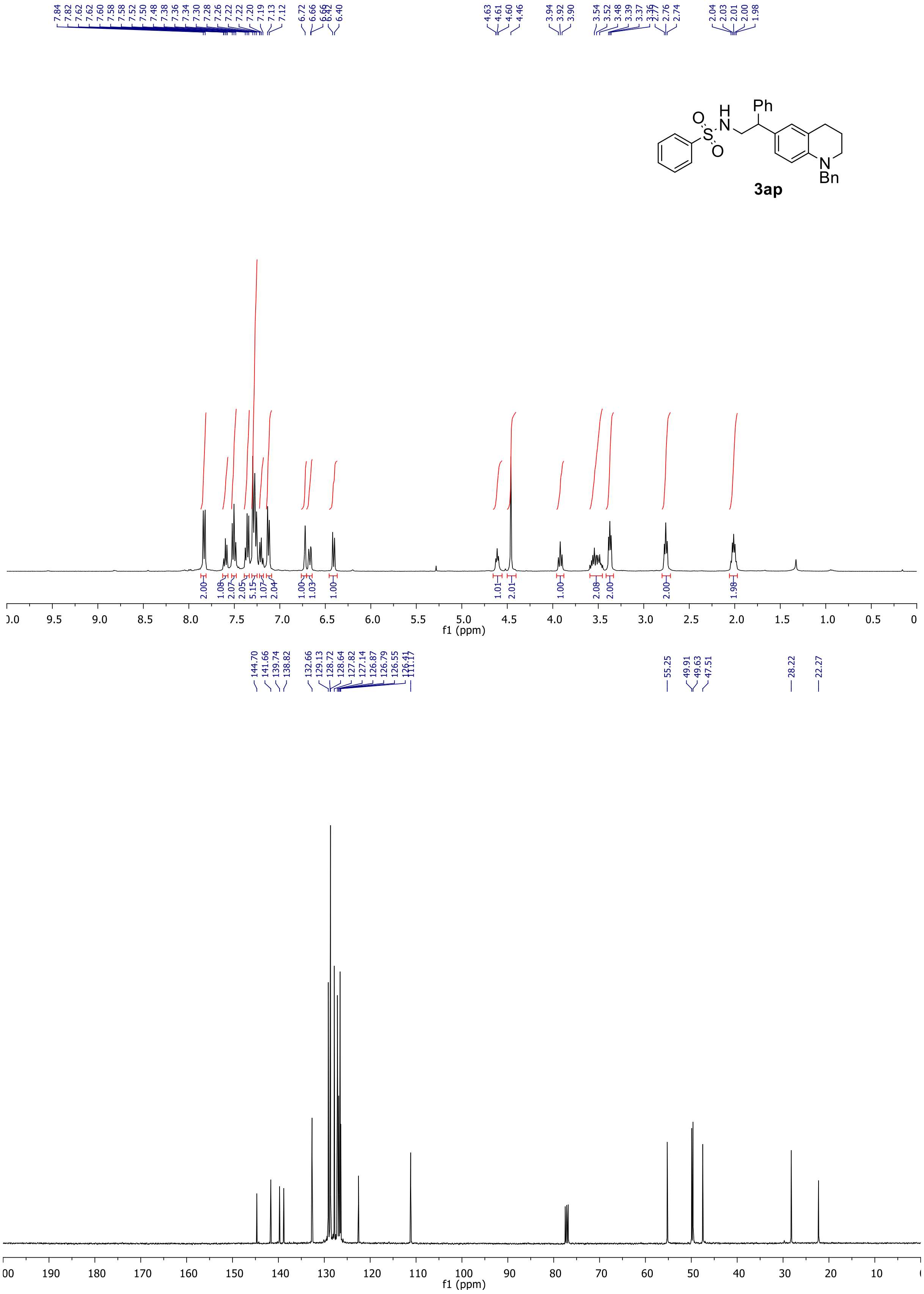

^{1}H NMR (400 MHz) and ^{13}C{^{1}H} NMR (100 MHz) spectra of **3ap** ($CDCl_3$)

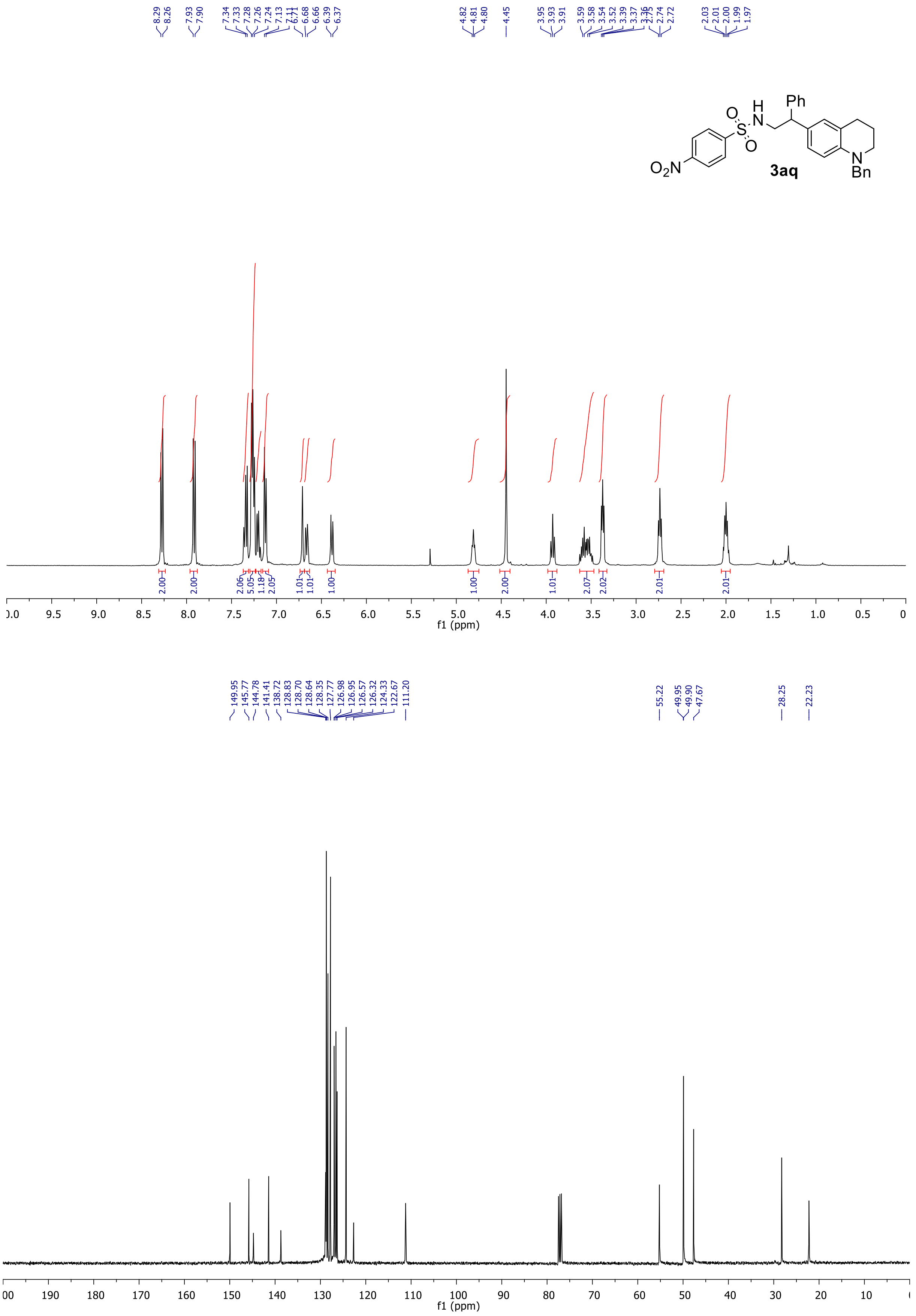

^{1}H NMR (400 MHz) and ^{13}C{^{1}H} NMR (100 MHz) spectra of **3aq** ($CDCl_3$)

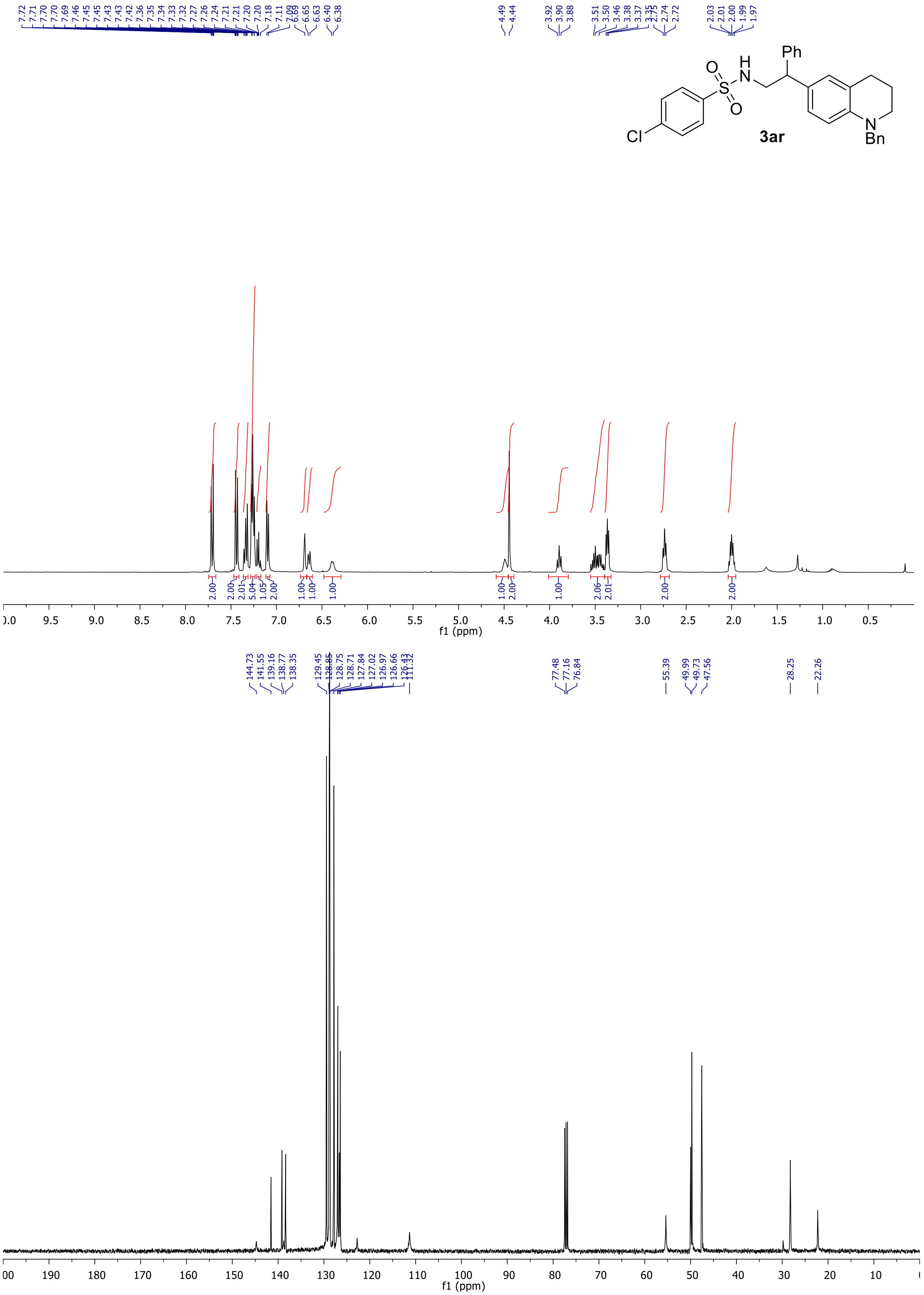

^{1}H NMR (400 MHz) and $^{13}C\{^{1}H\}$ NMR (100 MHz) spectra of **3ar** ($CDCl_3$)

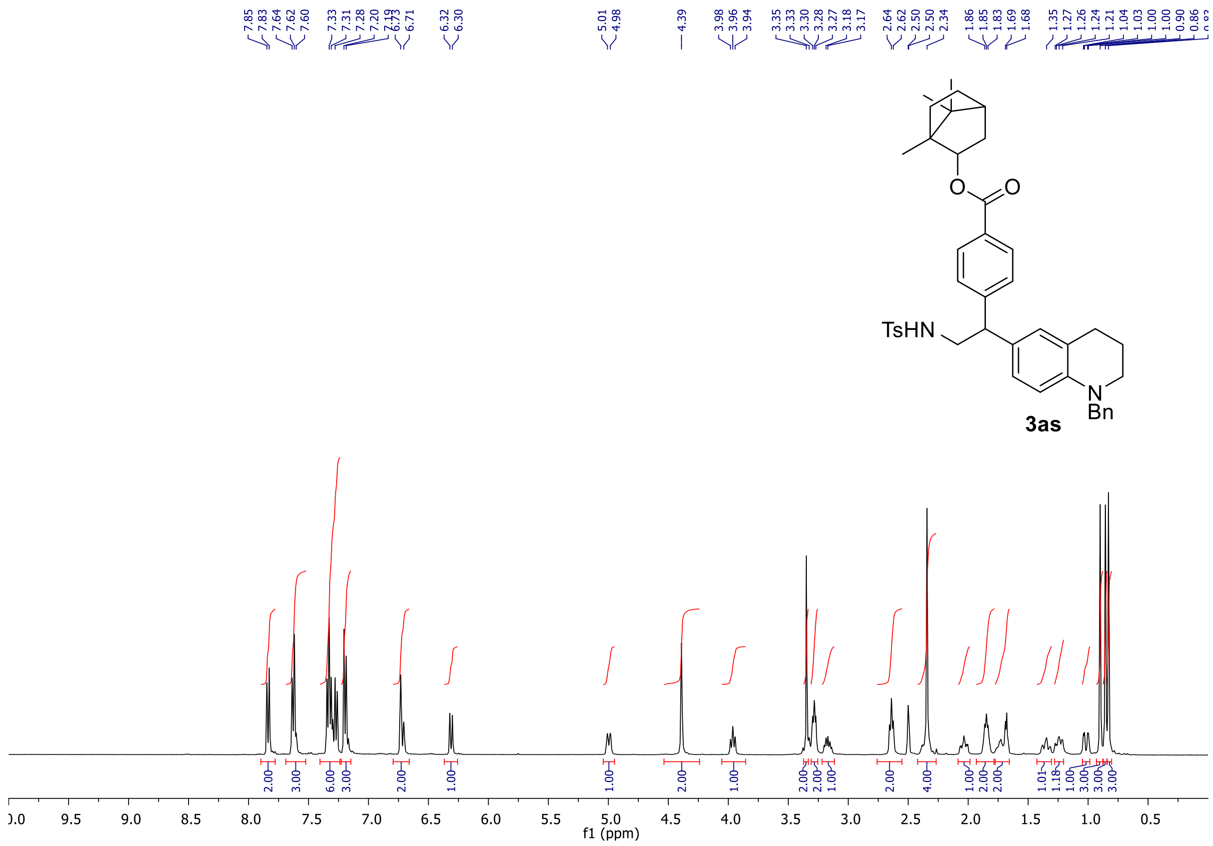

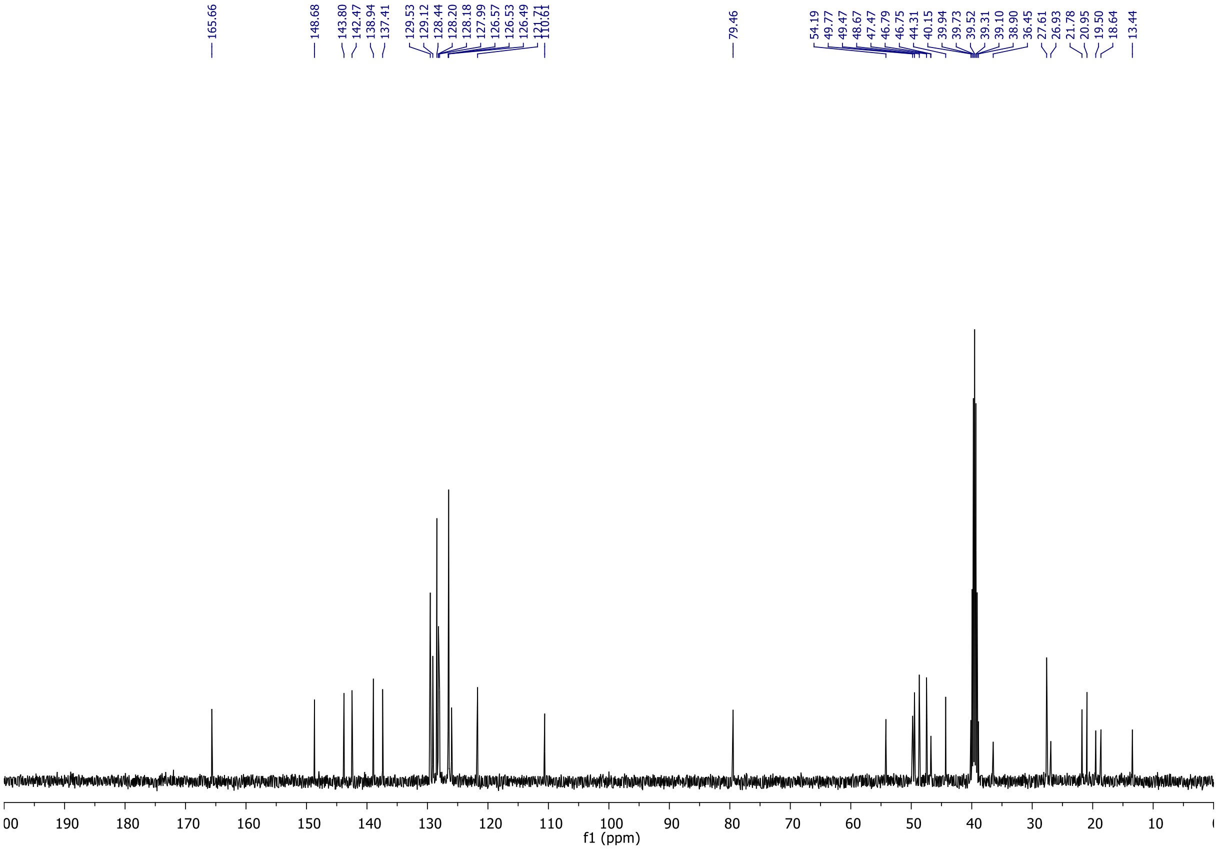

^{1}H NMR (400 MHz) and $^{13}C\{^{1}H\}$ NMR (100 MHz) spectra of **3as** (DMSO-d_6)

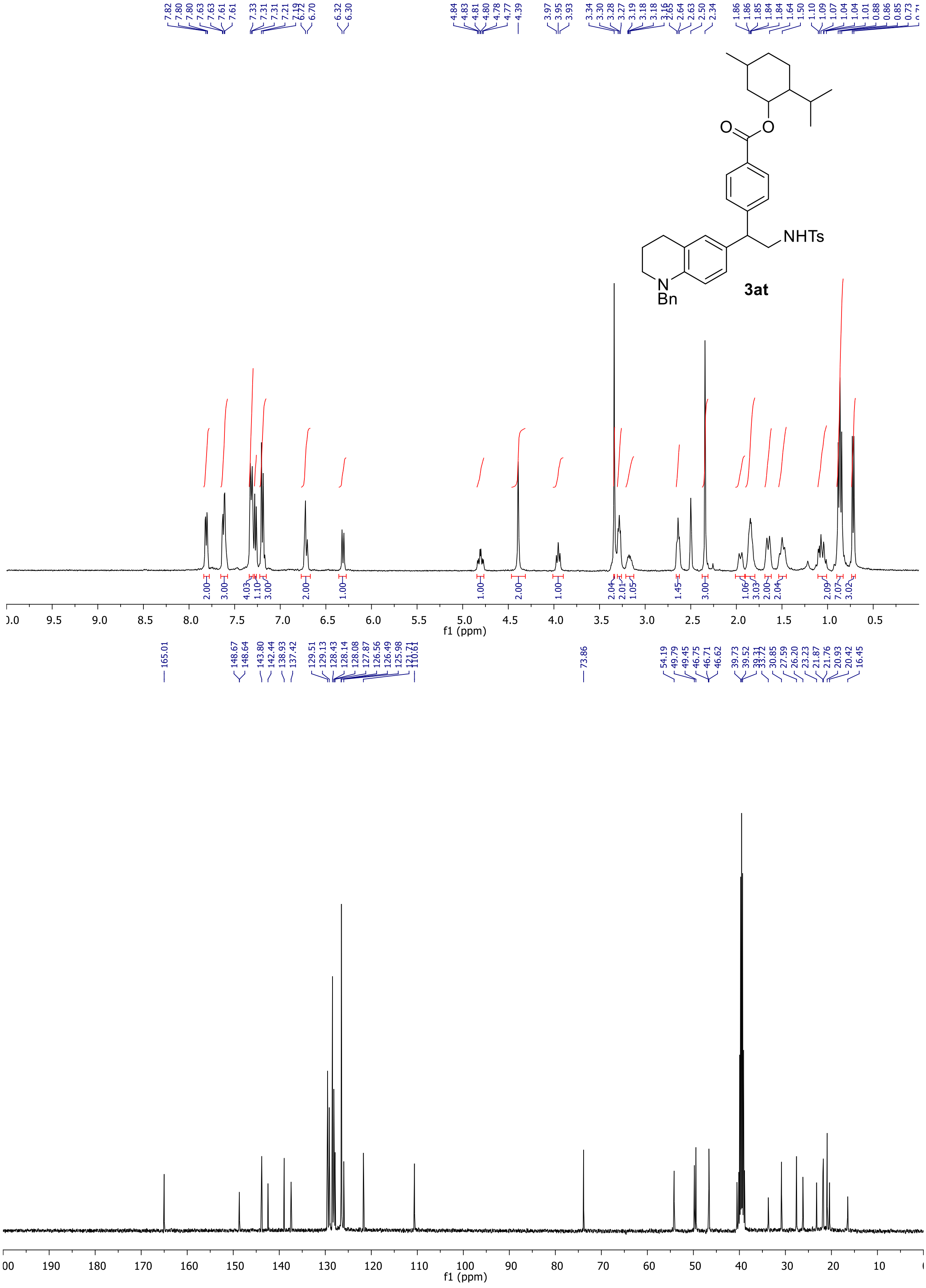

^{1}H NMR (400 MHz) and $^{13}C\{^{1}H\}$ NMR (100 MHz) spectra of **3at** (DMSO-d_6)

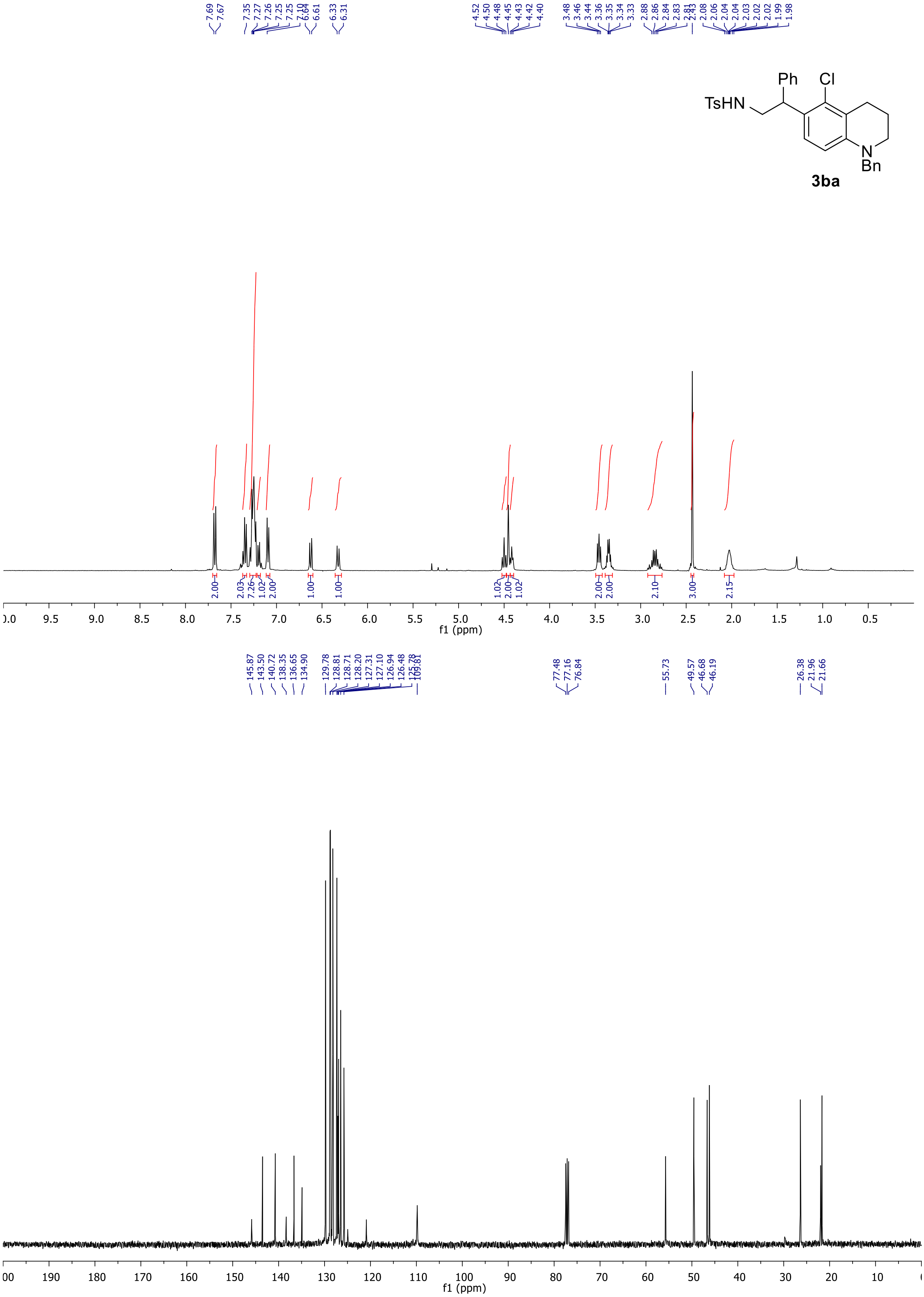

^{1}H NMR (400 MHz) and ^{13}C{^{1}H} NMR (100 MHz) spectra of **3ba** ($CDCl_3$)

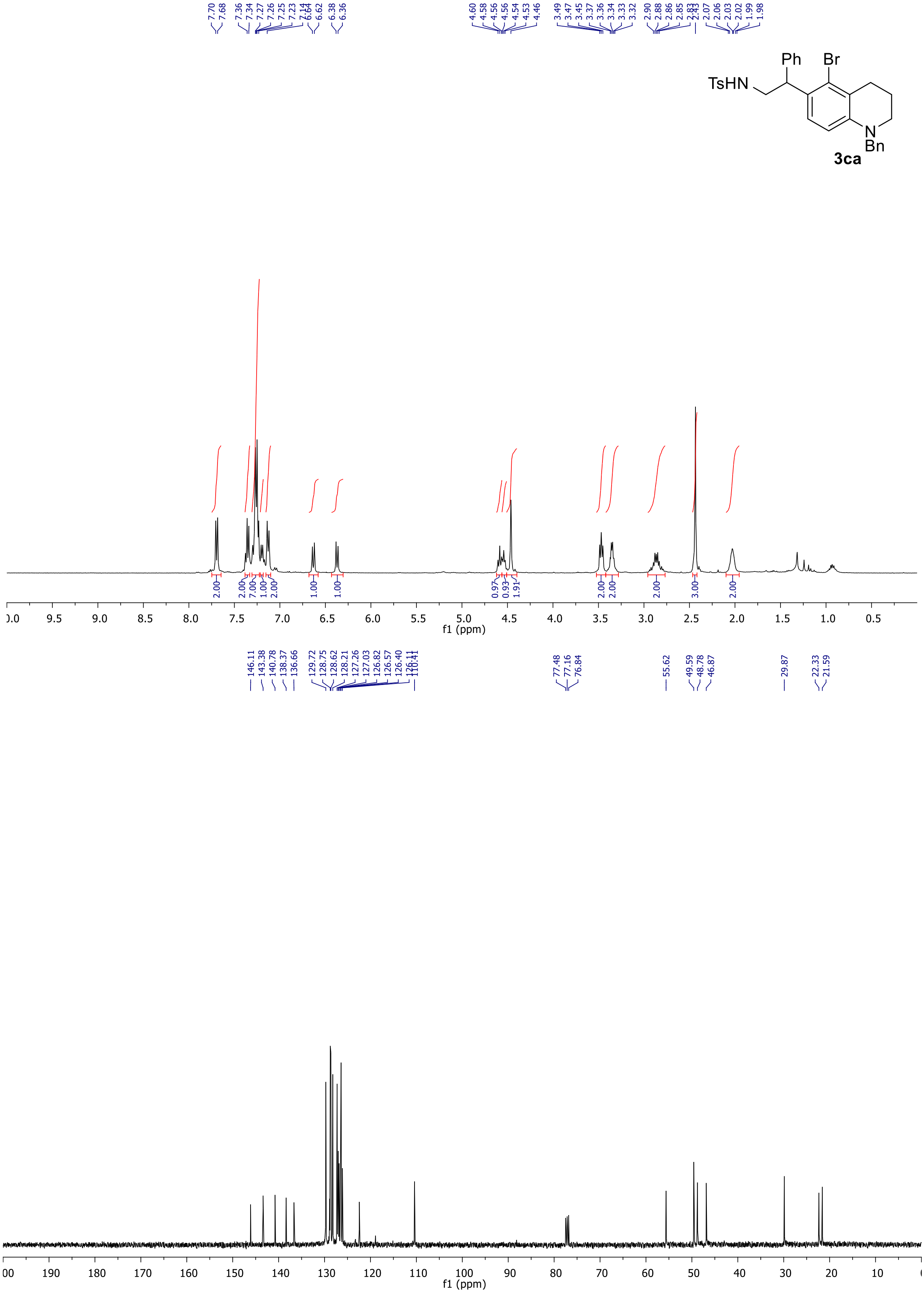

^{1}H NMR (400 MHz) and ^{13}C{^{1}H} NMR (100 MHz) spectra of **3ca** ($CDCl_3$)

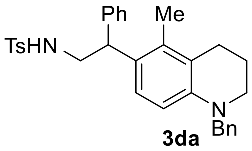

3da

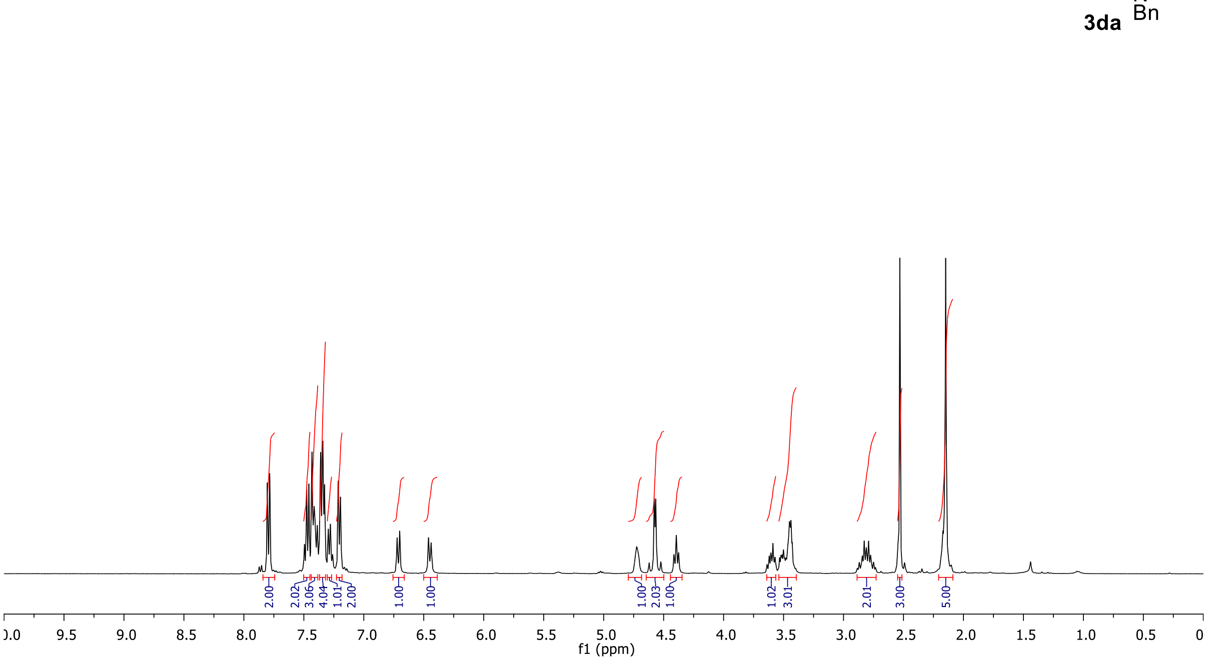

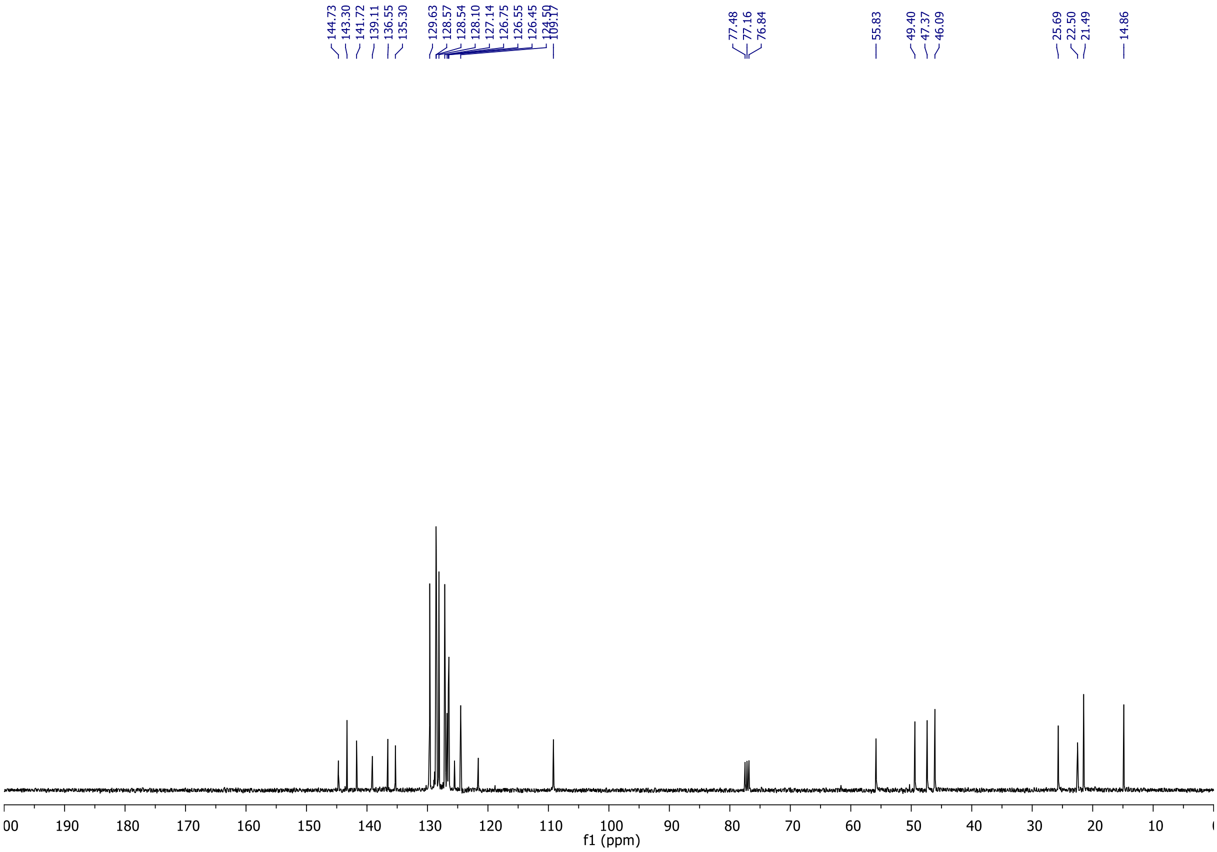

^{1}H NMR (400 MHz) and ^{13}C{^{1}H} NMR (100 MHz) spectra of **3da** ($CDCl_3$)

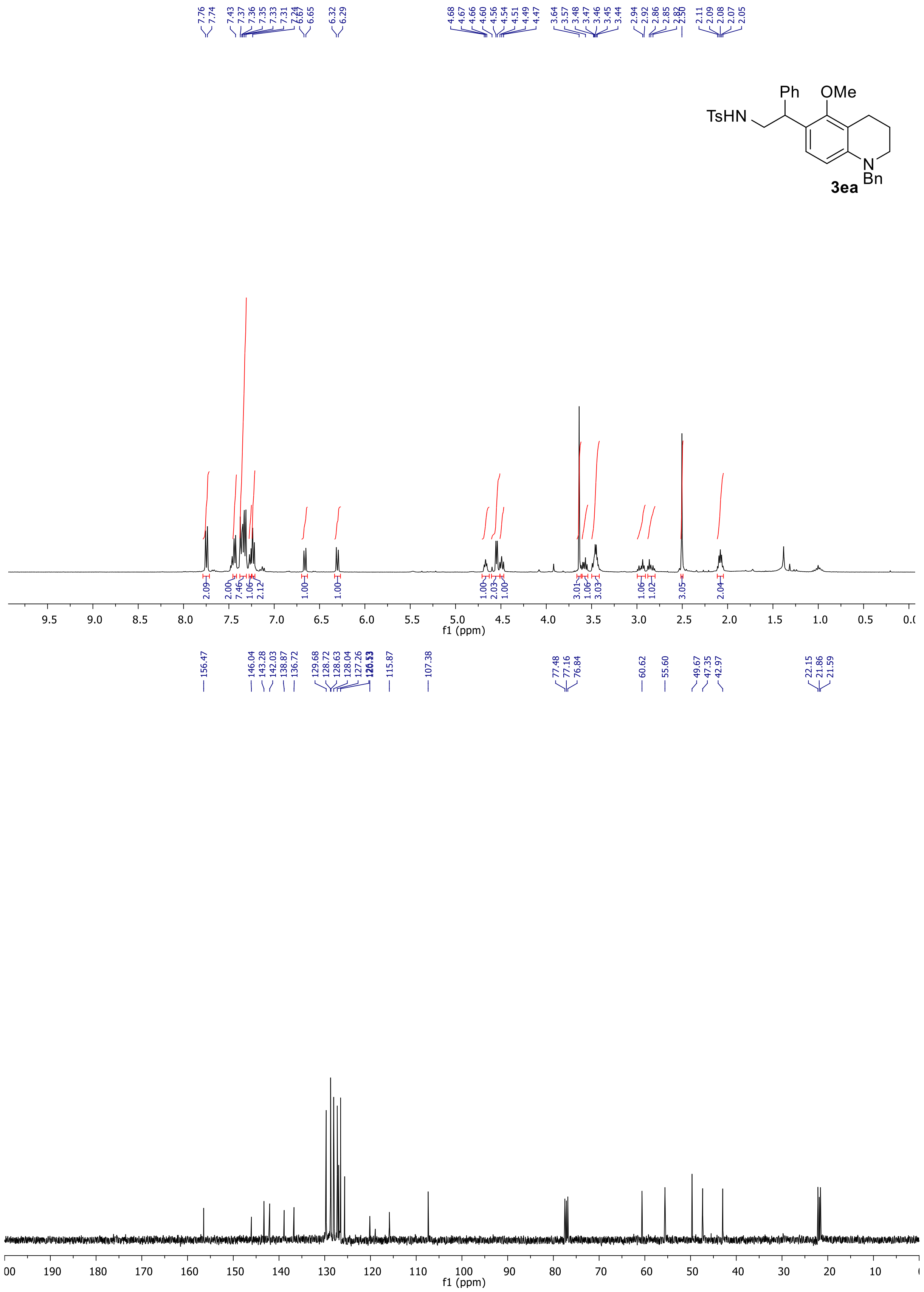

^{1}H NMR (400 MHz) and ^{13}C{^{1}H} NMR (100 MHz) spectra of **3ea** ($CDCl_3$)

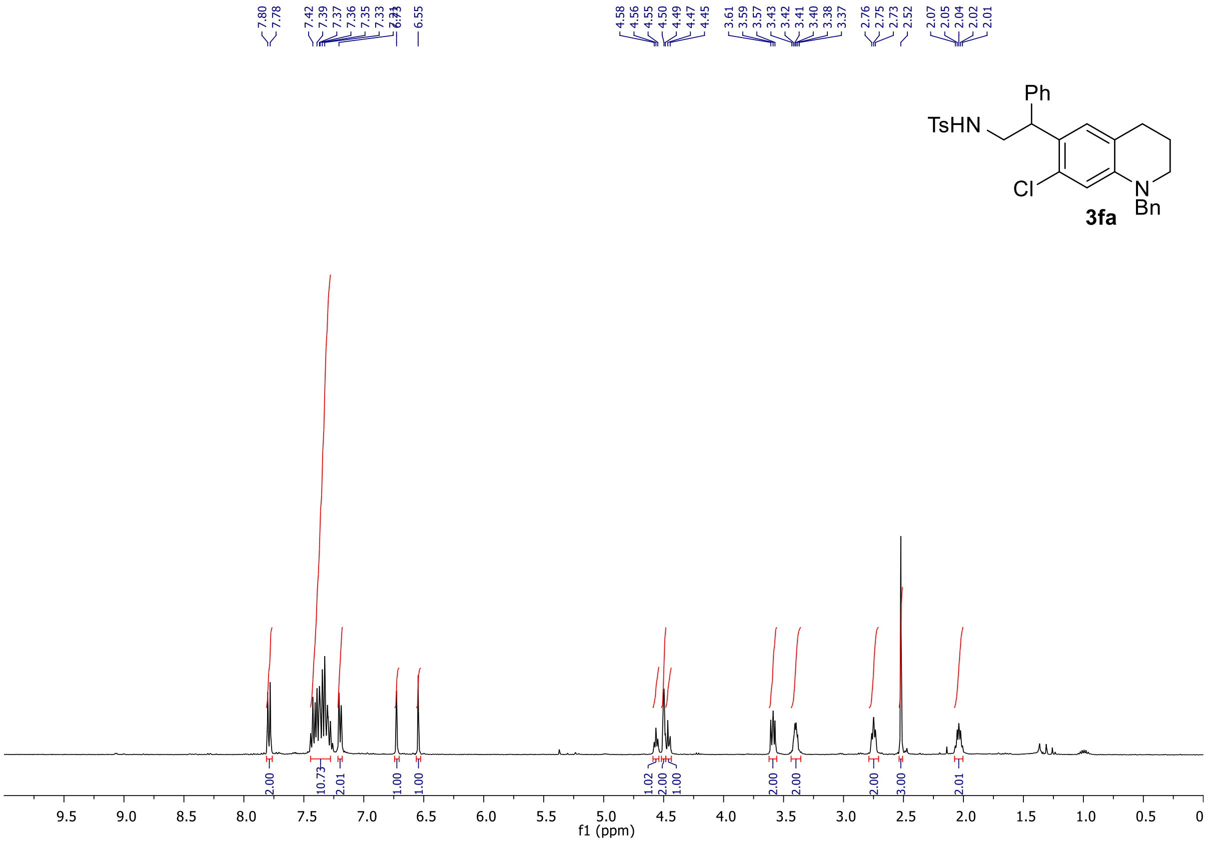

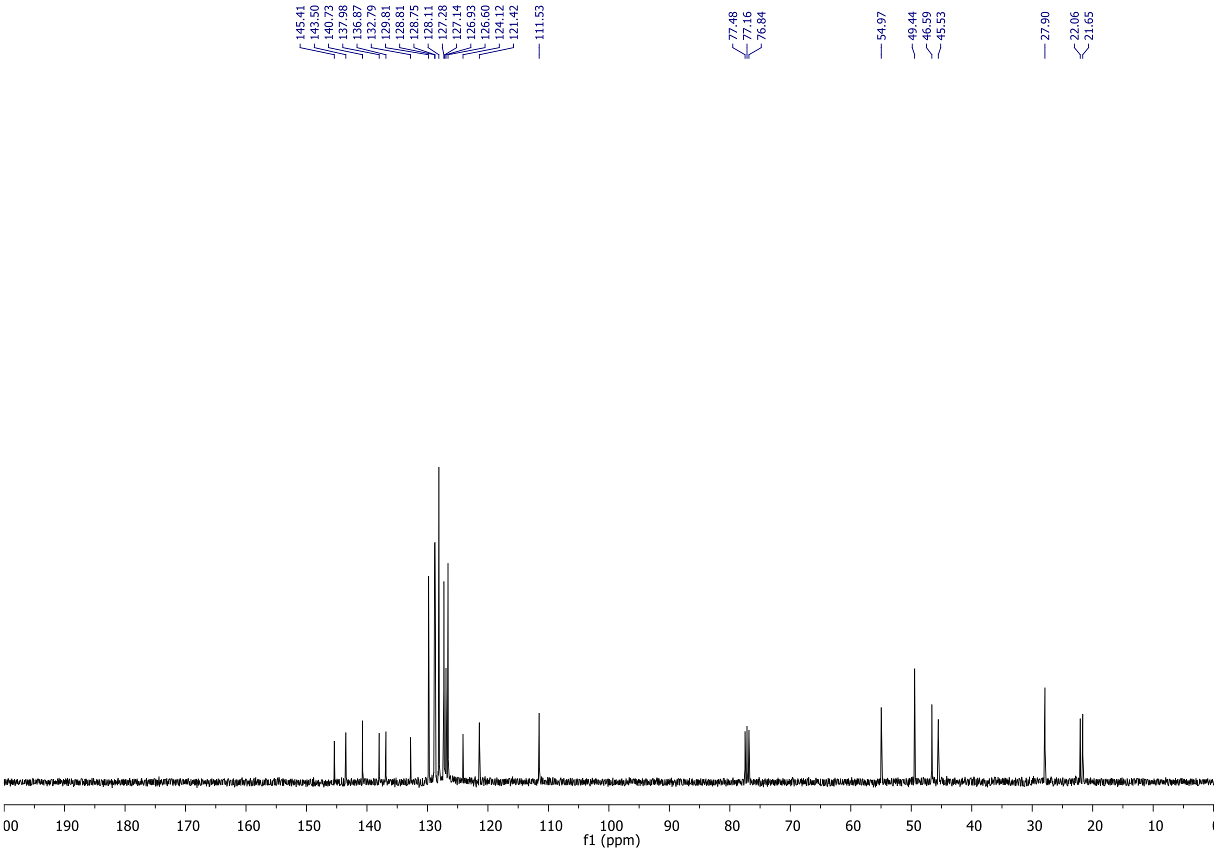

^{1}H NMR (400 MHz) and $^{13}C\{^{1}H\}$ NMR (100 MHz) spectra of **3fa** ($CDCl_3$)

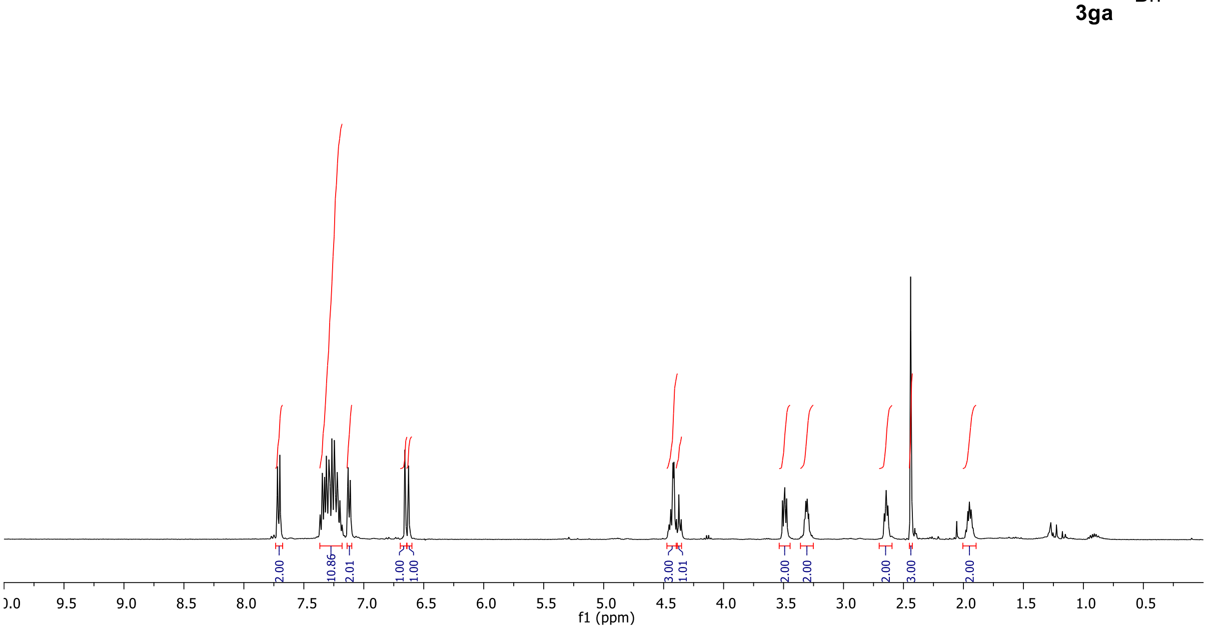

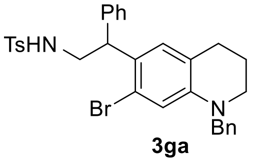

3ga

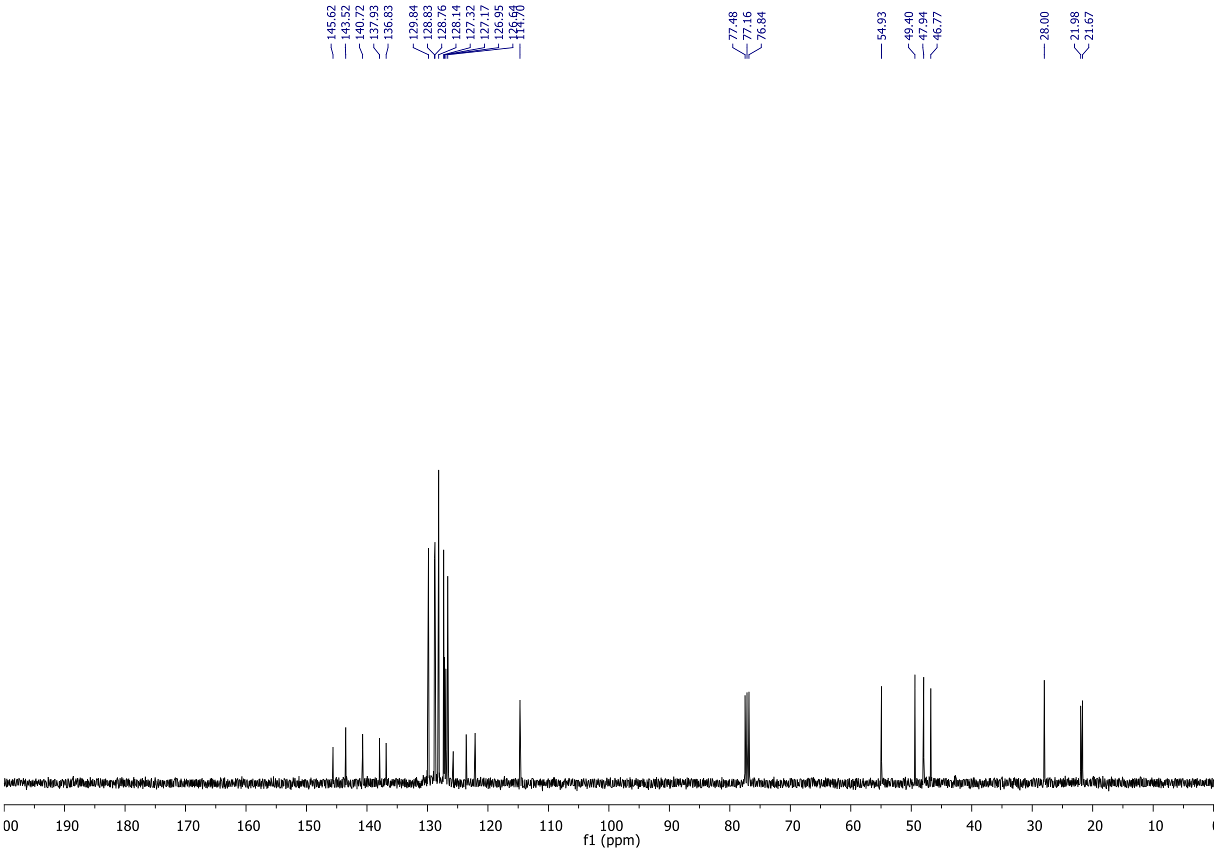

^{1}H NMR (400 MHz) and ^{13}C{^{1}H} NMR (100 MHz) spectra of **3ga** ($CDCl_3$)

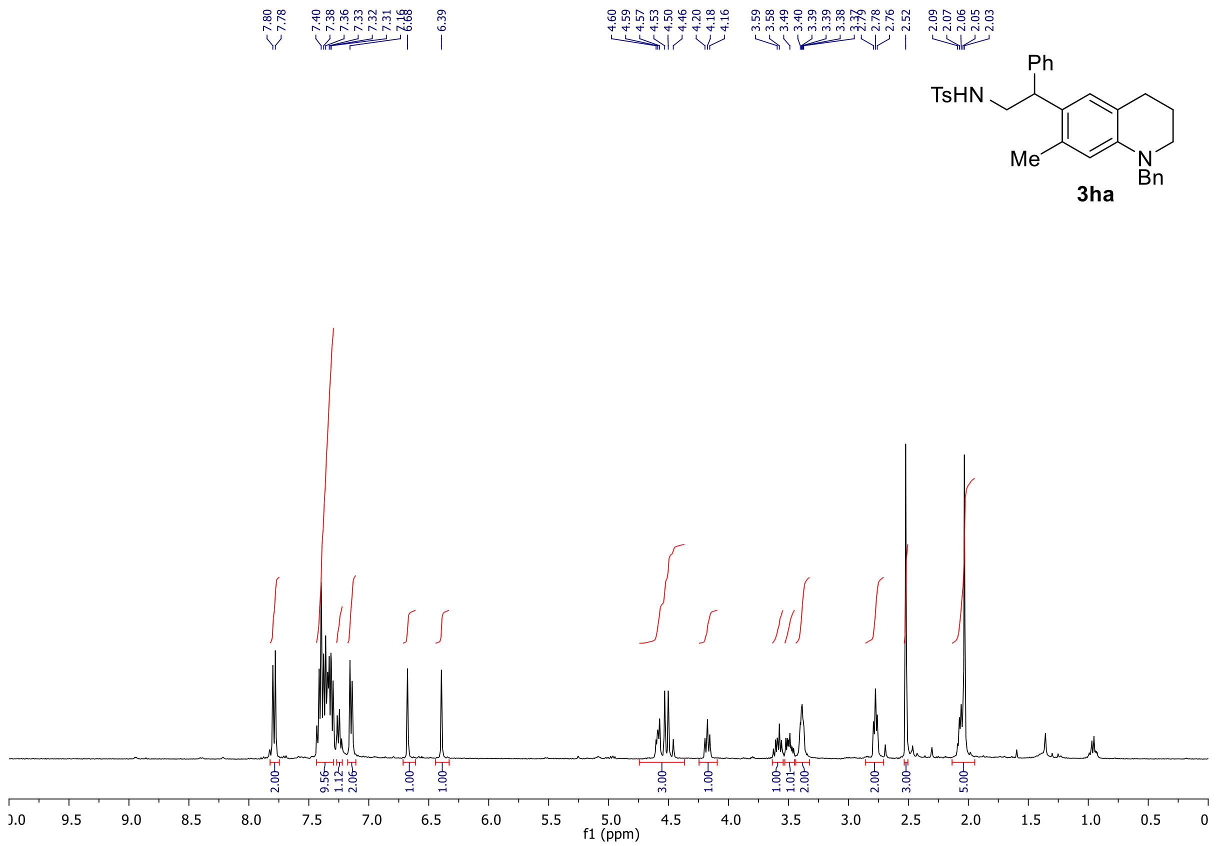

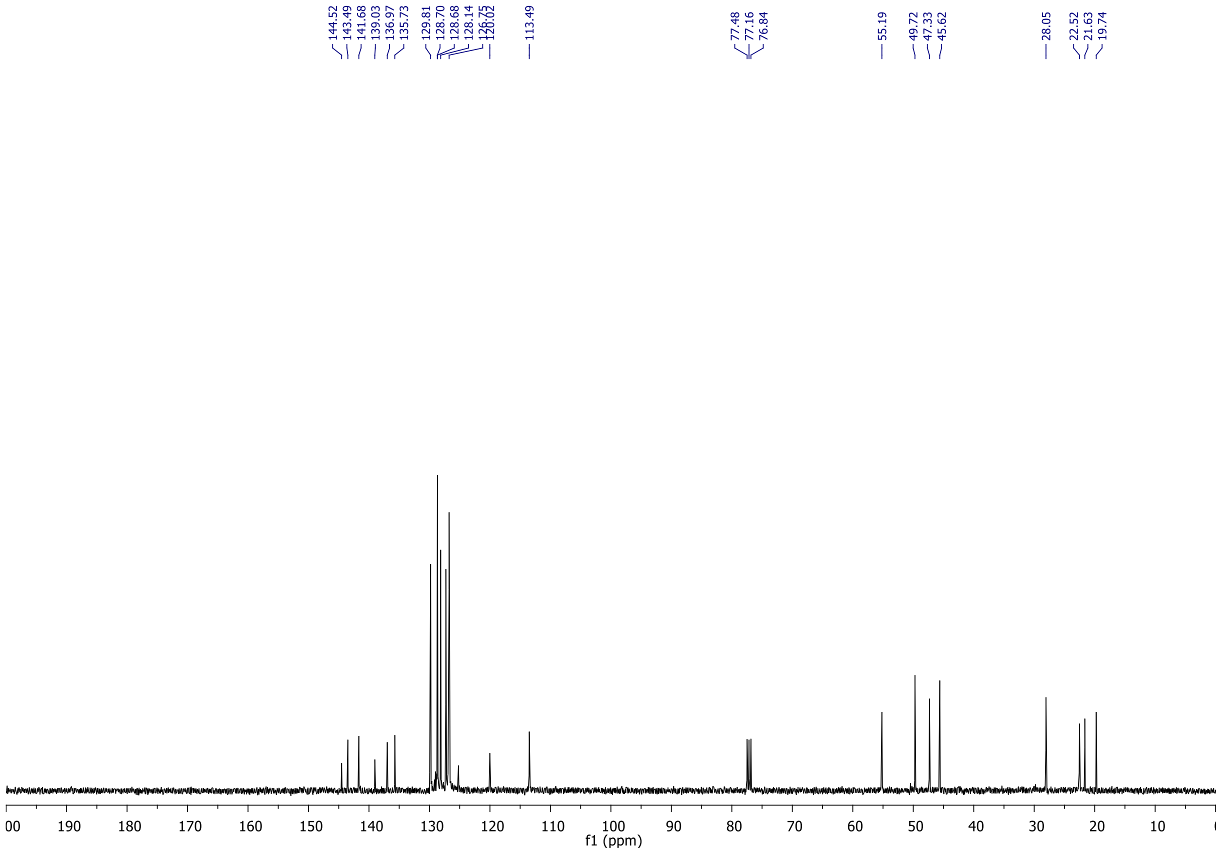

^{1}H NMR (400 MHz) and ^{13}C{^{1}H} NMR (100 MHz) spectra of **3ha** (CDCl$_3$)

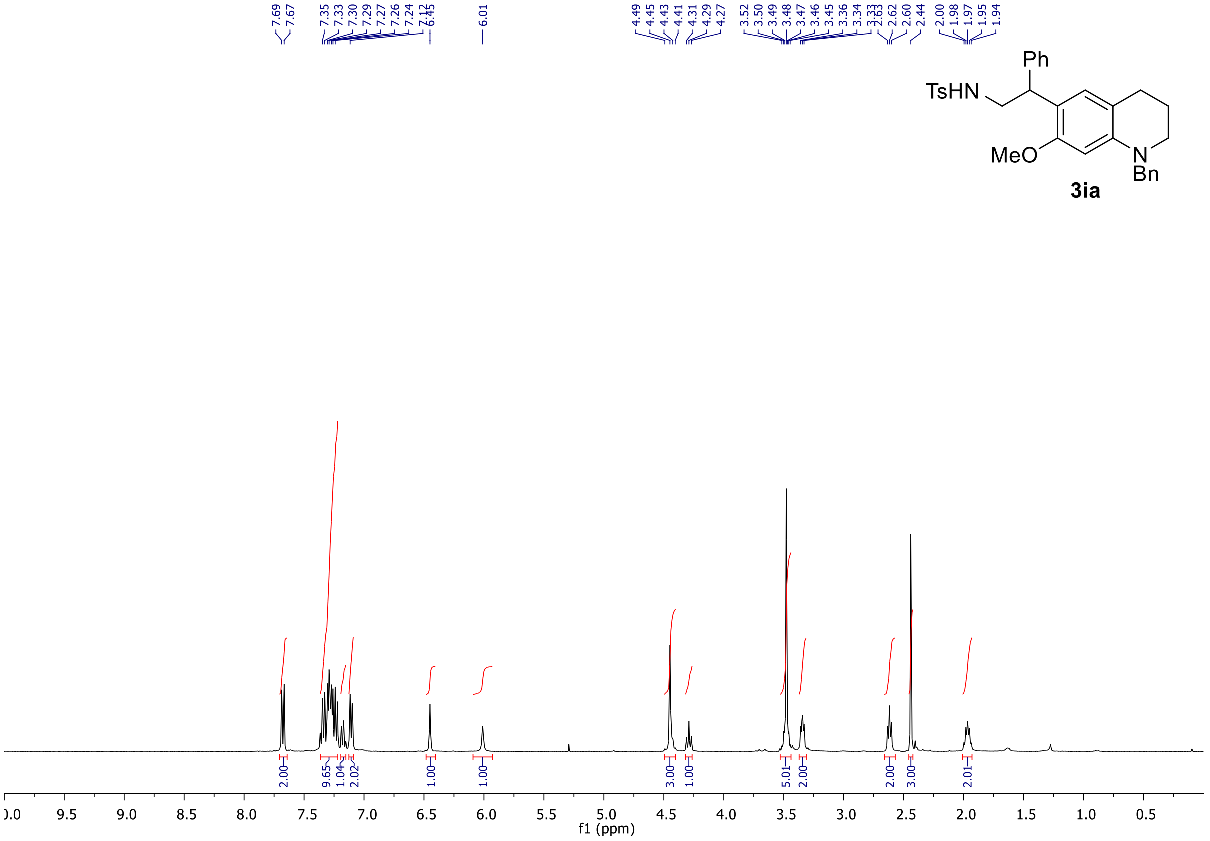

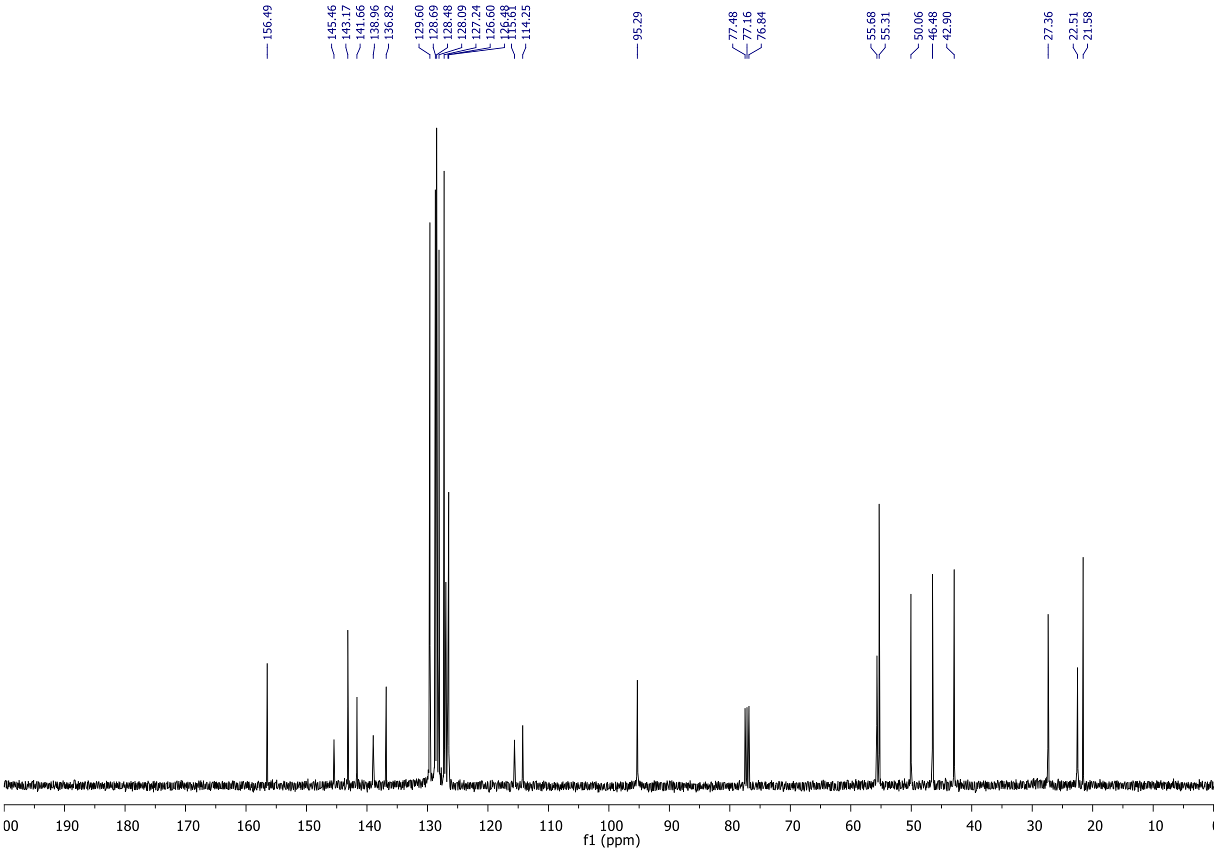

^{1}H NMR (400 MHz) and $^{13}C\{^{1}H\}$ NMR (100 MHz) spectra of **3ia** ($CDCl_3$)

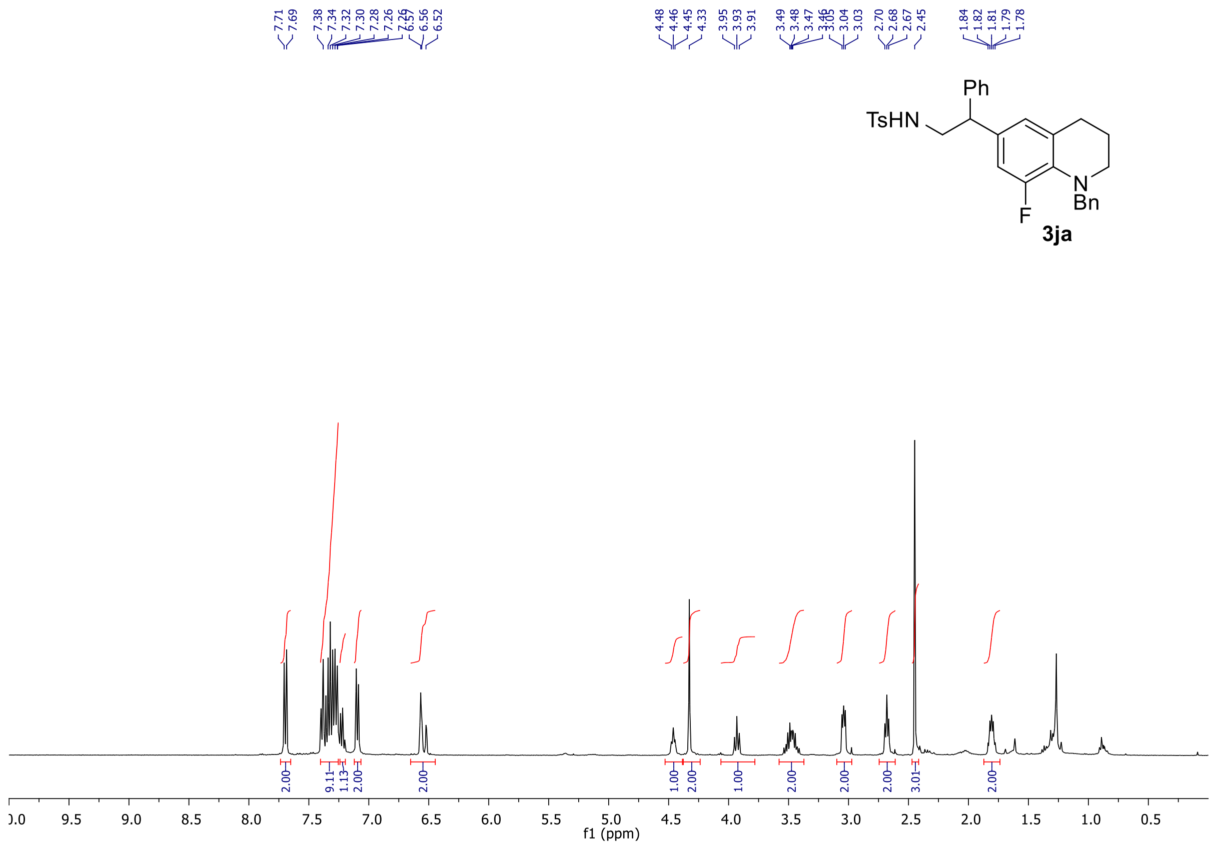

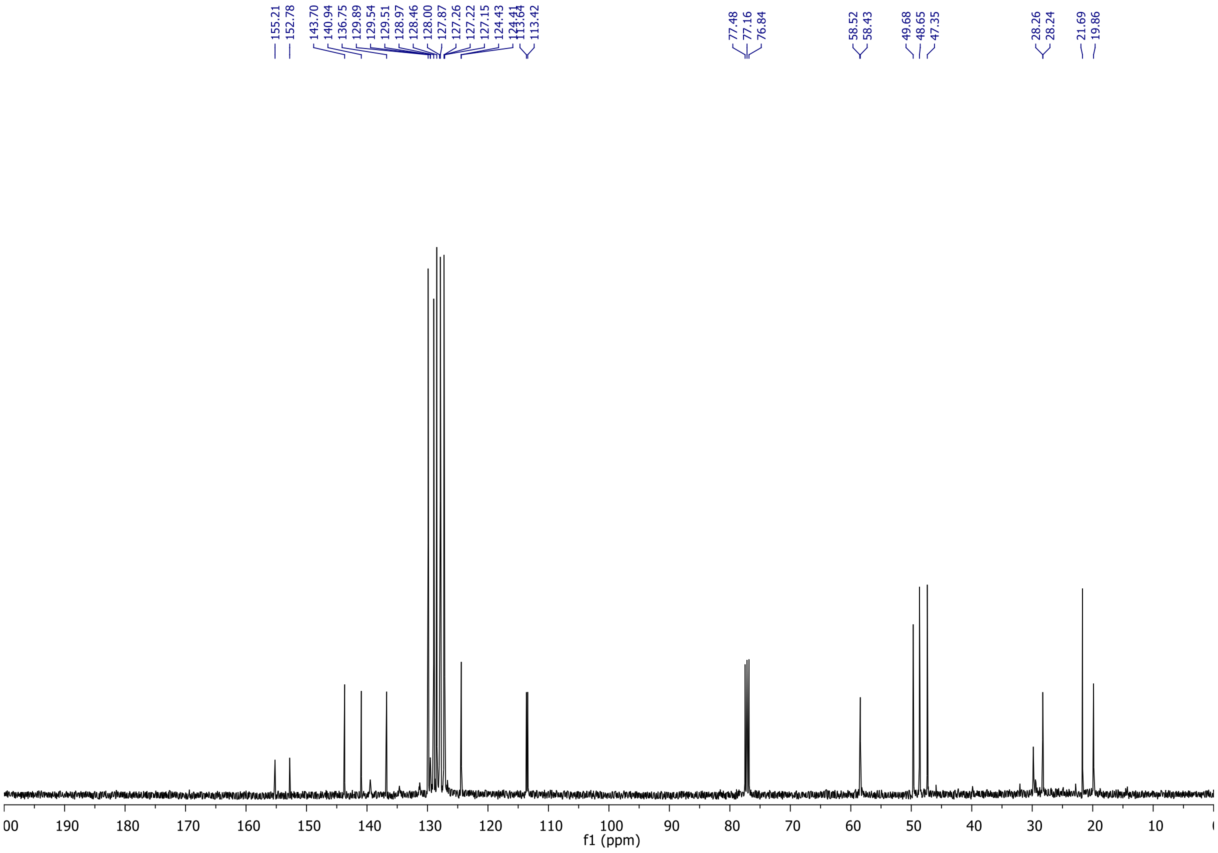

^{1}H NMR (400 MHz) and $^{13}C\{^{1}H\}$ NMR (100 MHz) spectra of **3ja** ($CDCl_3$)

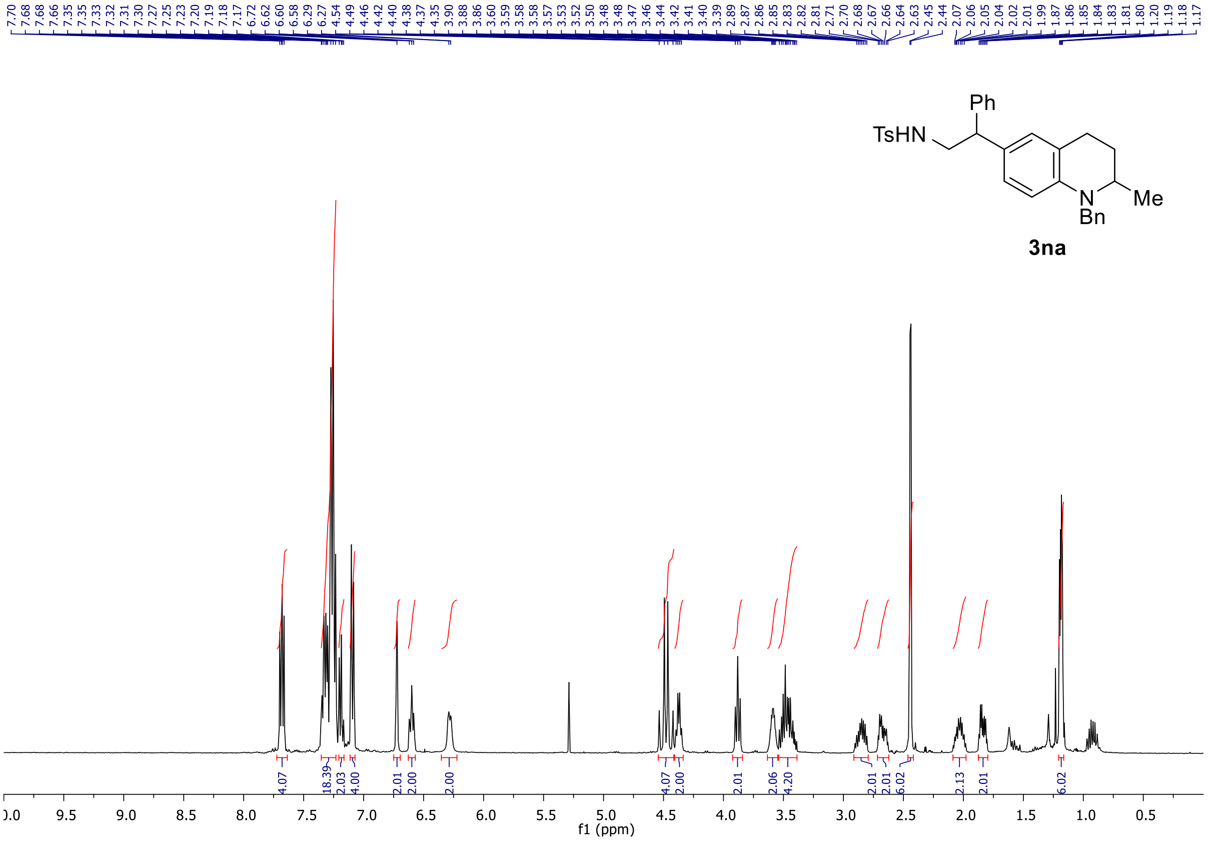

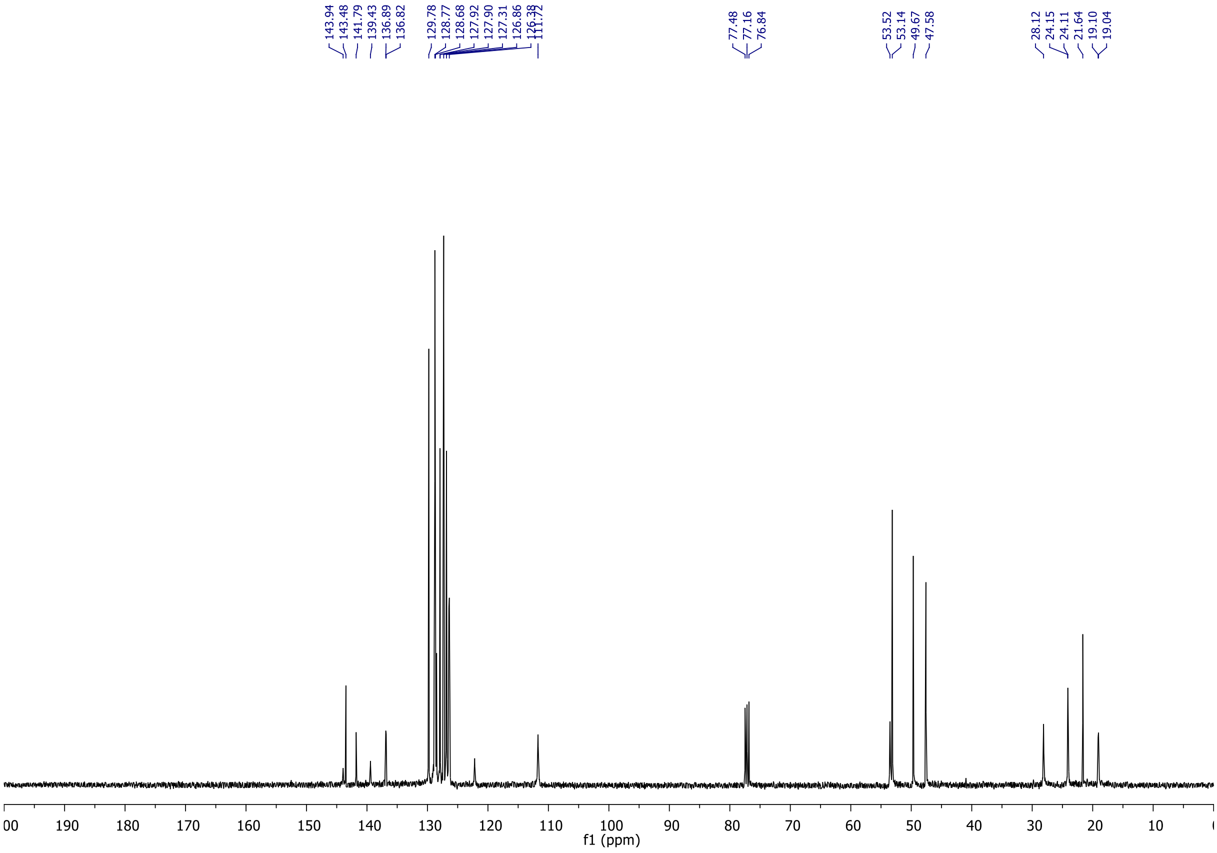

^{1}H NMR (400 MHz) and $^{13}C\{^{1}H\}$ NMR (100 MHz) spectra of **3na** ($CDCl_3$)

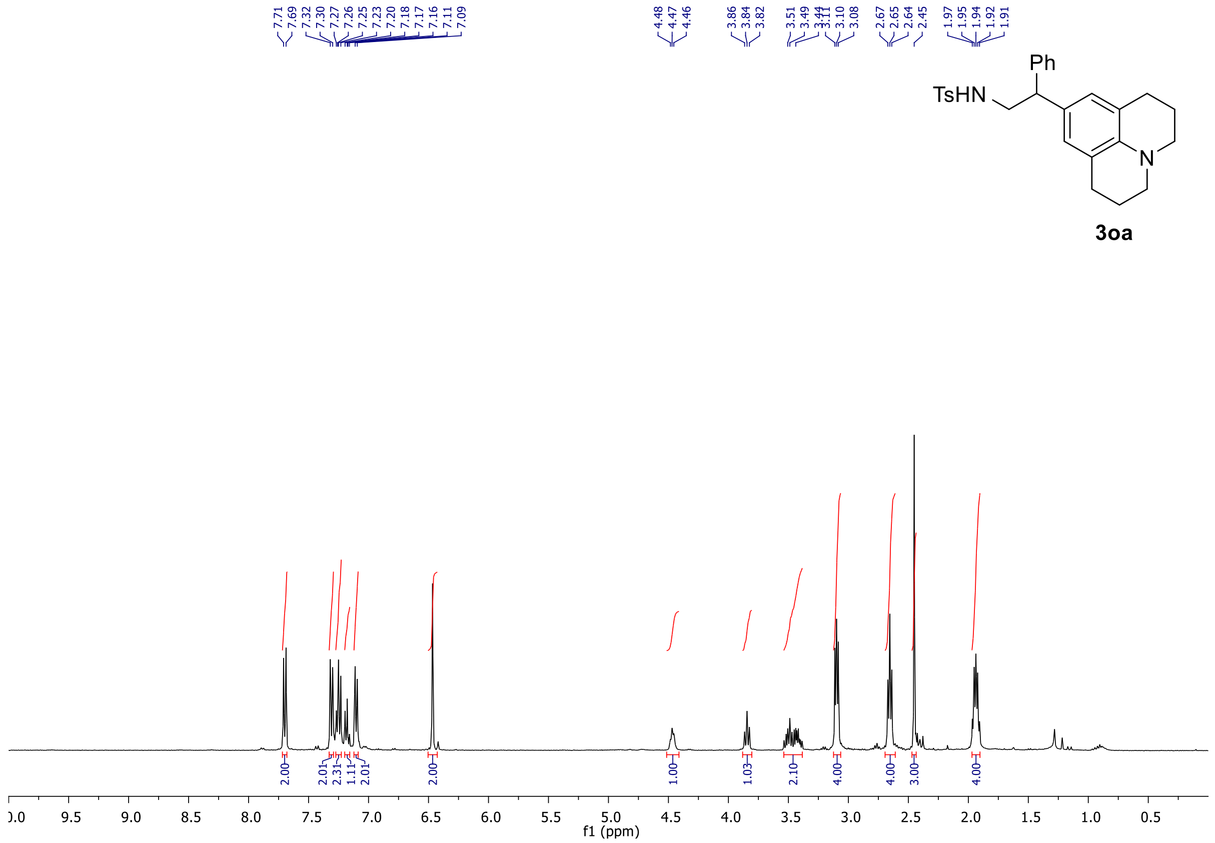

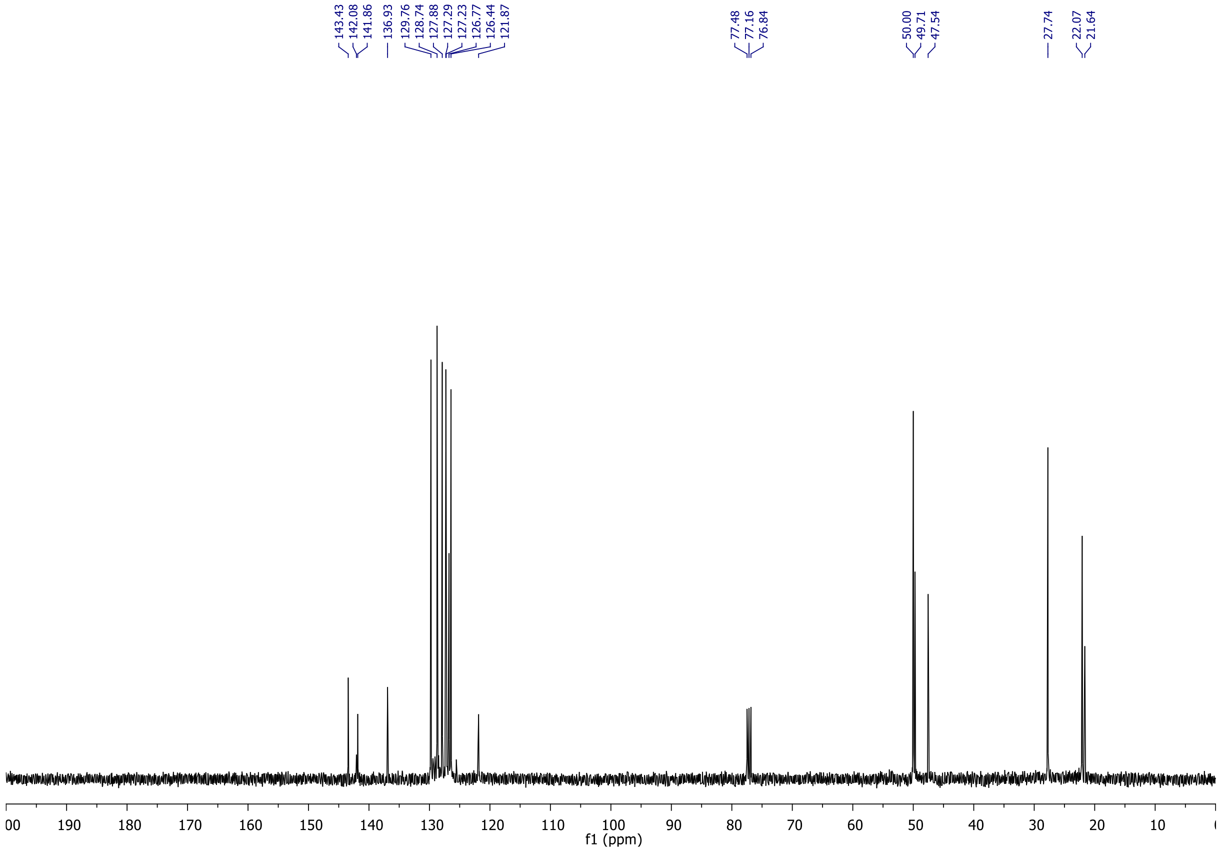

^{1}H NMR (400 MHz) and ^{13}C{^{1}H} NMR (100 MHz) spectra of **3oa** ($CDCl_3$)

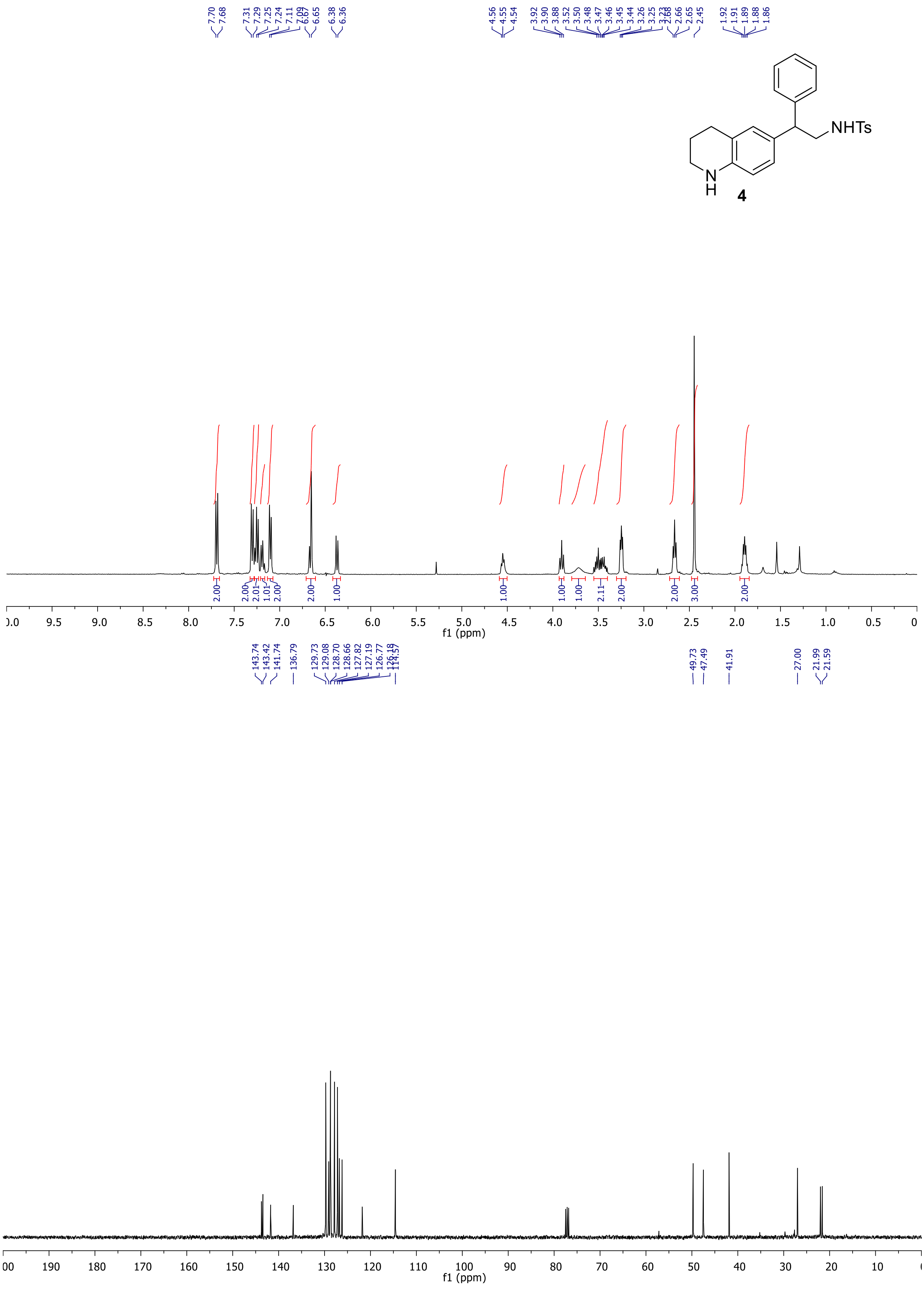

^{1}H NMR (400 MHz) and $^{13}C\{^{1}H\}$ NMR (100 MHz) spectra of **4** ($CDCl_3$)

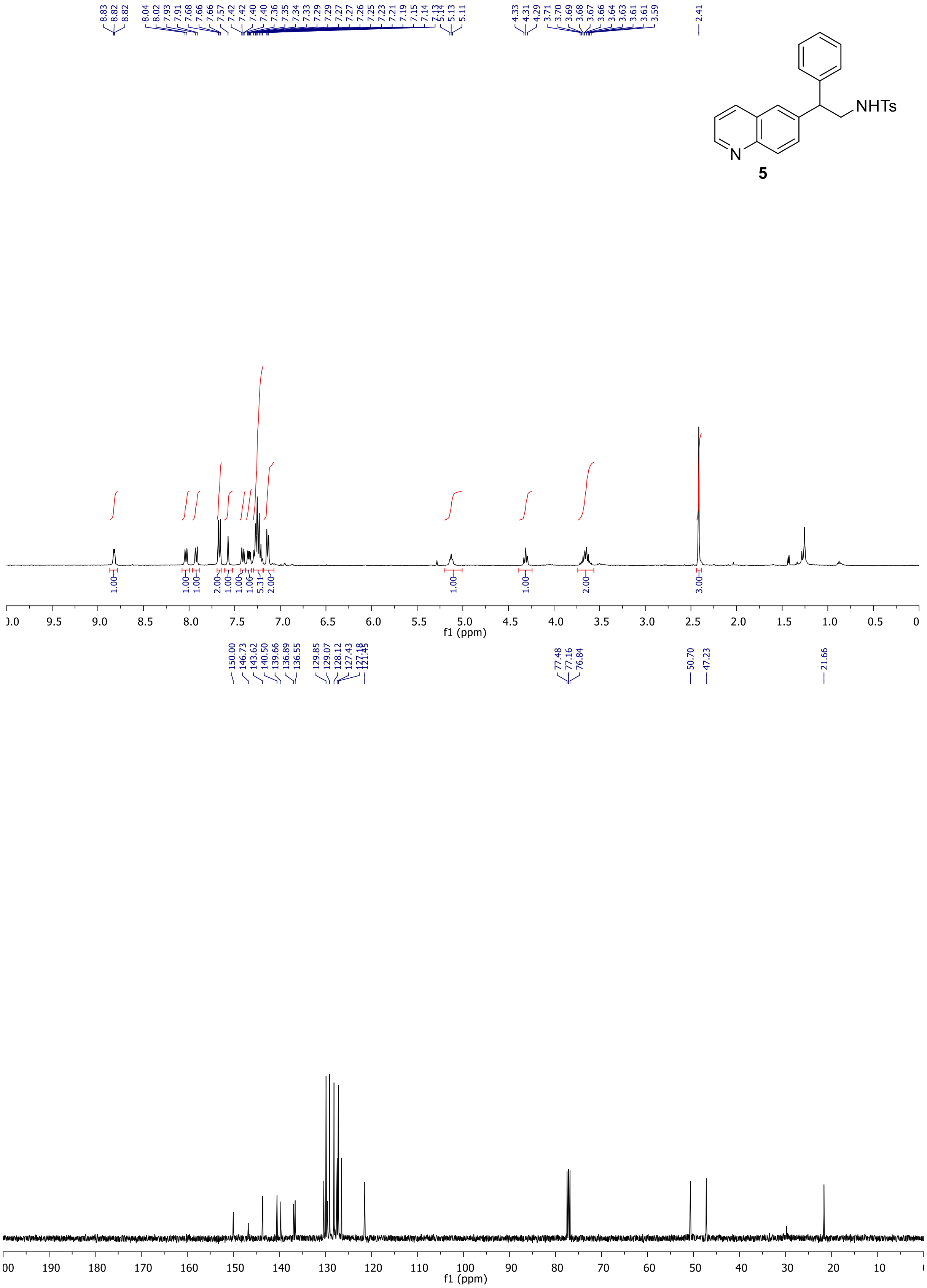

^{1}H NMR (400 MHz) and ^{13}C{^{1}H} NMR (100 MHz) spectra of **5** ($CDCl_3$)

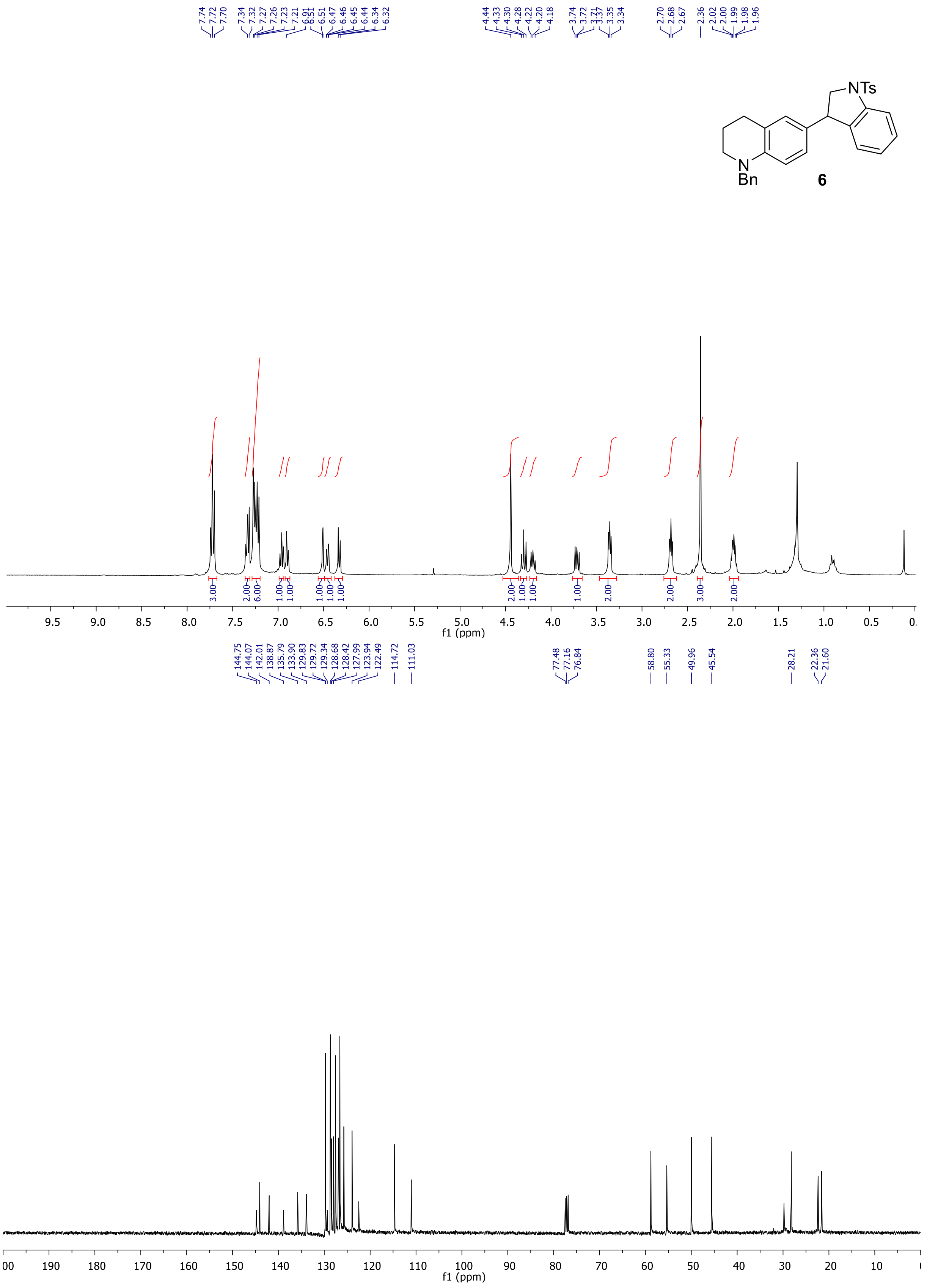

^{1}H NMR (400 MHz) and ^{13}C{^{1}H} NMR (100 MHz) spectra of **6** ($CDCl_3$)

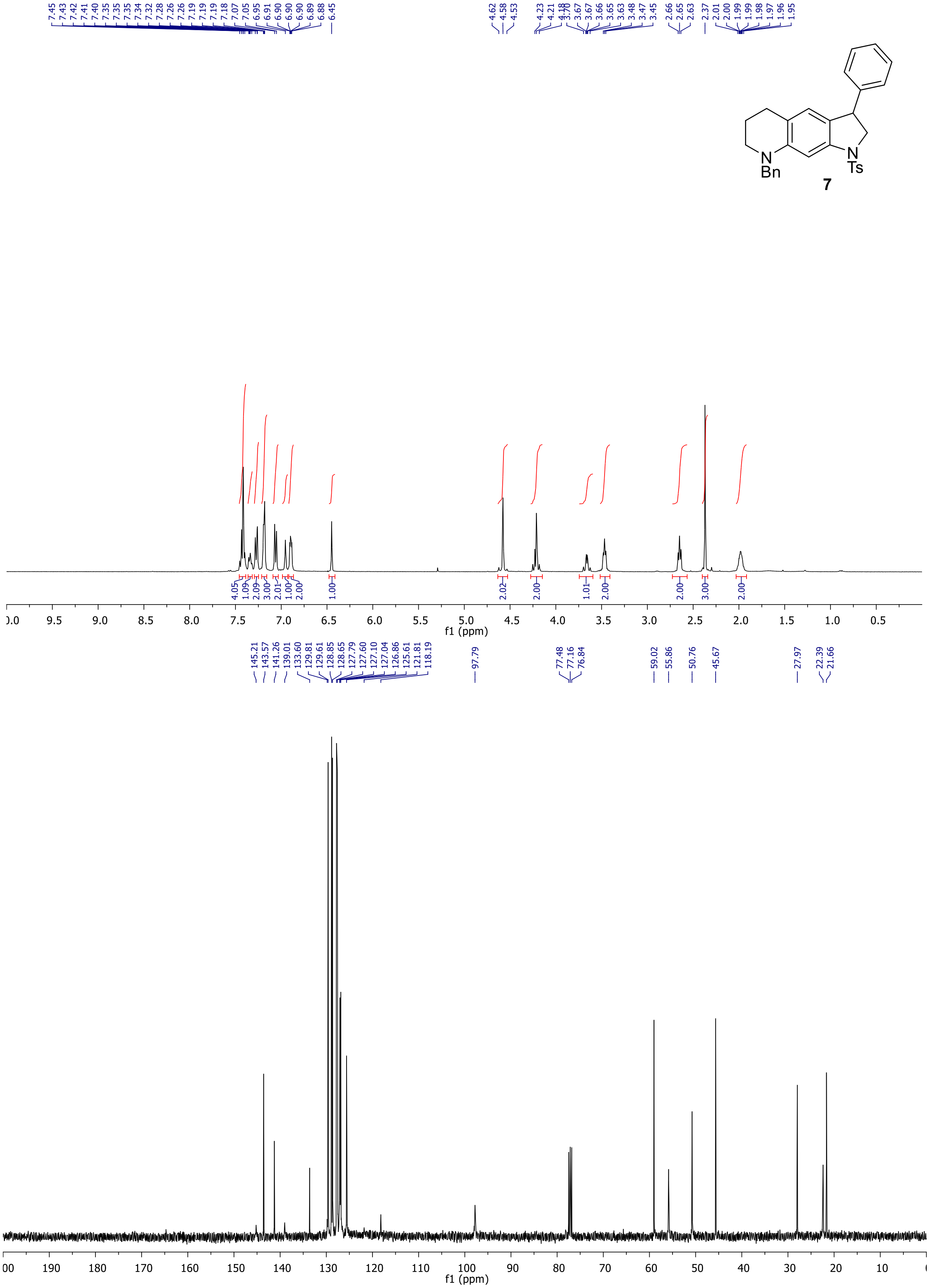

^{1}H NMR (400 MHz) and ^{13}C{^{1}H} NMR (100 MHz) spectra of **7** ($CDCl_3$)

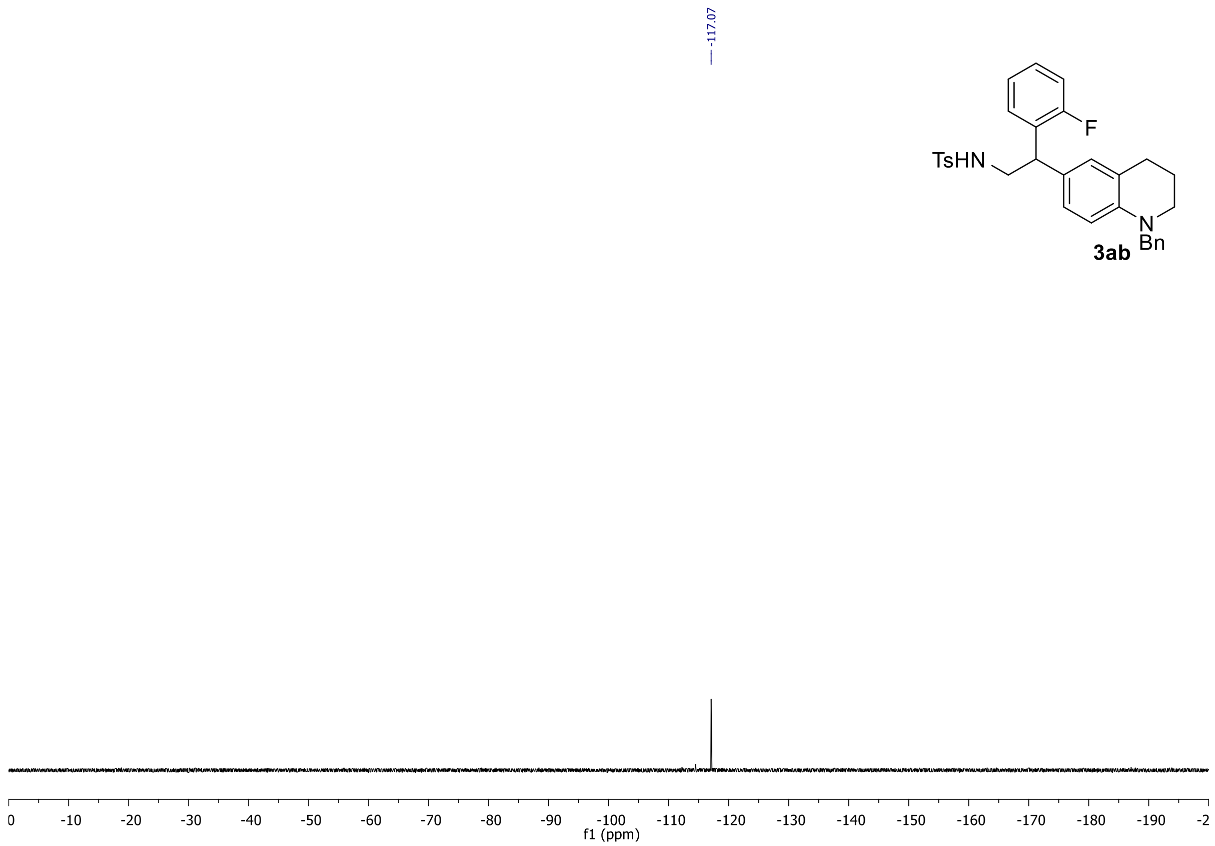

^{19}F NMR (376 MHz) spectra of **3ab** ($CDCl_3$)

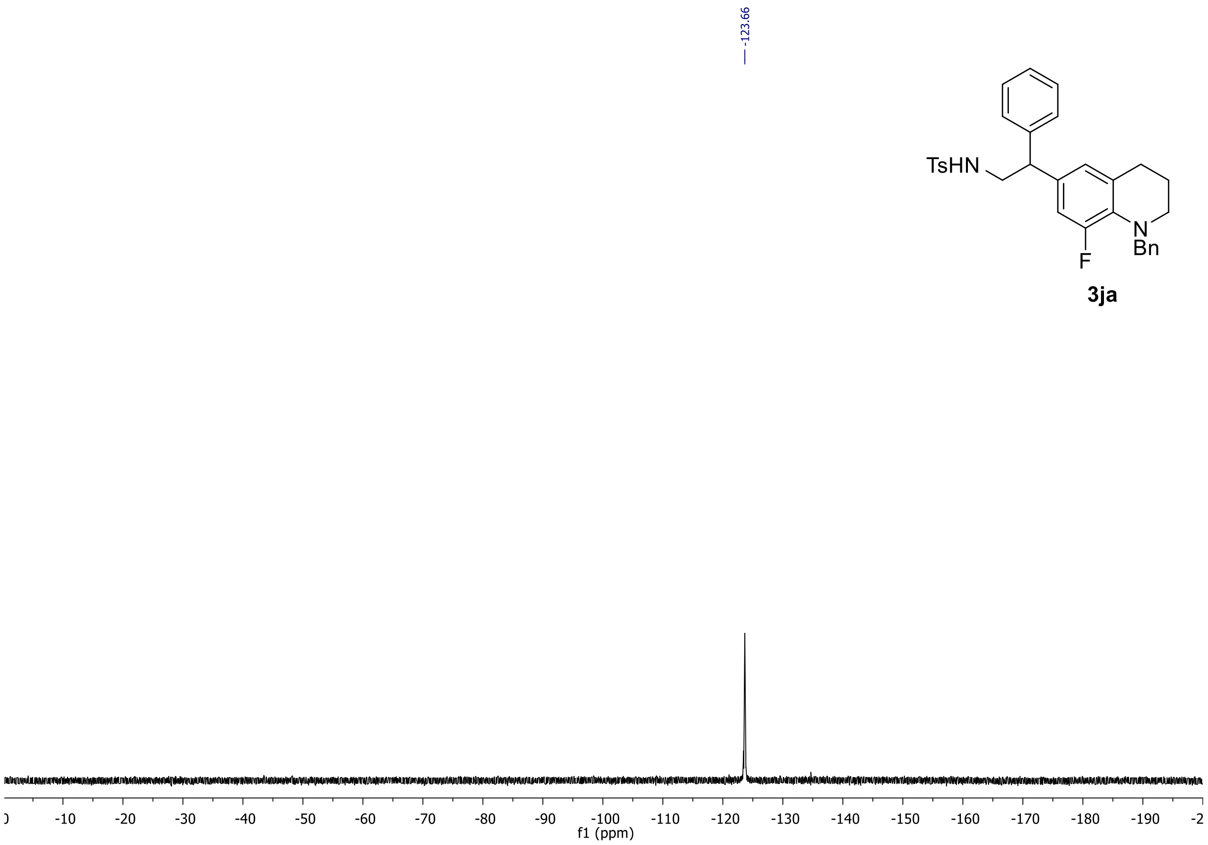

^{19}F NMR (376 MHz) spectra of **3ja** ($CDCl_3$)
